# Living GenoChemetics by hyphenating synthetic biology and synthetic chemistry in vivo

**DOI:** 10.1038/s41467-017-00194-3

**Published:** 2017-08-09

**Authors:** Sunil V. Sharma, Xiaoxue Tong, Cristina Pubill-Ulldemolins, Christopher Cartmell, Emma J. A. Bogosyan, Emma J. Rackham, Enrico Marelli, Refaat B. Hamed, Rebecca J. M. Goss

**Affiliations:** 1 School of Chemistry, University of St Andrews, North Haugh, St Andrews KY16 9ST UK; 2 BSRC, University of St Andrews, North Haugh, St Andrews KY16 9ST UK; 3 School of Chemistry, University of East, Norwich, NR4 7TJ UK; 4 Analytical Development, GSK, Cobden Street, Montrose, Angus DD10 8EA UK; 5 School of Medicine, University of East Anglia, Bob Champion Research and Education Building, James Watson Road, Norwich, NR4 7UQ UK

## Abstract

Marrying synthetic biology with synthetic chemistry provides a powerful approach toward natural product diversification, combining the best of both worlds: expediency and synthetic capability of biogenic pathways and chemical diversity enabled by organic synthesis. Biosynthetic pathway engineering can be employed to insert a chemically orthogonal tag into a complex natural scaffold affording the possibility of site-selective modification without employing protecting group strategies. Here we show that, by installing a sufficiently reactive handle (e.g., a C–Br bond) and developing compatible mild aqueous chemistries, synchronous biosynthesis of the tagged metabolite and its subsequent chemical modification in living culture can be achieved. This approach can potentially enable many new applications: for example, assay of directed evolution of enzymes catalyzing halo-metabolite biosynthesis in living cells or generating and following the fate of tagged metabolites and biomolecules in living systems. We report synthetic biological access to new-to-nature bromo-metabolites and the concomitant biorthogonal cross-coupling of halo-metabolites in living cultures.

## Abstract (web summary)

Coupling synthetic biology and chemical reactions in cells is a challenging task. The authors engineer bacteria capable of generating bromo-metabolites, develop a mild Suzuki-Miyaura cross-coupling reaction compatible with cell growth and carry out the cross-coupling chemistry in live cell cultures.

## Introduction

Bacterial natural products represent an unparalleled starting point for drug discovery^1^, and there is much interest in the generation of analogs of such compounds in order to explore modes of action, determine structure-activity-relationships and improve bioavailability and bioactivity. Natural products and their analogs may of course be accessed through total synthesis; excellent recent examples include the total synthesis of the antibiotic marinomycin^2^ and generation of rifamycin and the structurally related metabolite salinisporamycin^3, 4^. Such studies are invaluable in developing methodology, revealing potential biogenic mechanisms and providing the only access to molecules generated by rare or hard to culture organisms. However, the approach of total synthesis can be challenging, time consuming and costly. Analog generation through semi-synthesis is also limiting; with the selective chemical modification of complex molecules remaining as a longstanding challenge within chemical synthesis, and usually demanding the presence of innate chemical orthogonality within the molecule. To overcome such obstacles, Miller has pioneered the use of peptides to catalyze site-selective epoxidation^5^, acylation^6^ and halogenation^7^ of complex scaffolds, enabling access to novel antibiotic analogs; nevertheless, a range of regioisomers still results.

The enzymatic introduction of a halogen into a compound is regioselective and provides a chemically orthogonal handle to enable its further direct functionalization through cross-coupling methodologies; we have previously developed such a GenoChemetic strategy, but were limited in vivo to chlorination^8^. Analogous to our GenoChemetic approach, Miller has combined his peptide enabled selective bromination with diversification using Suzuki–Miyaura cross-coupling. This is exemplified in his regioselective functionalisation of vancomycin through a stepwise monodehalogenation/cross-coupling strategy of its native aryl chlorides^9^ and his two step derivatization of teicoplanin^10^. In a complementary approach to Miller, Lewis has engineered the halogenase RebH to enable regioselective and late-stage in vitro bromination of synthetic scaffolds^11^. The use of a membrane-partitioned reactor, to segregate a stabilized halogenase enzyme aggregate (crosslinked enzyme aggregates (CLEAs)) from a palladium catalyst, enabled the ‘almost one-pot’ C-H functionalization of aryl compounds to be achieved at 50–80 °C on a 3-indole propionate substrate^12^.

The cross-coupling of the highly reactive C-I bonds of 4-I-phenylalanine residues in modified proteins^13^ and the cross-coupling of aryltriflates in living cells has also been demonstrated^14^. In these studies, the aryl iodide and triflate substrates utilized, were synthesized separately and exogenously supplied.

Building upon our GenoChemetics approach to generate natural product analogues^8^, we wished to explore the ambitious goal of genetically engineering the biosynthesis of a metabolite tagged with a sufficiently reactive, chemically orthogonal handle. Such an approach would allow the tagged metabolite to be a good substrate for subsequent palladium-mediated modification in the presence of the living cells that produced it. While the incorporation of a C–I bond provides a highly reactive site for functionalization, so far, no enzymes capable of iodination have been characterized and therefore other halogenases must be employed. The engineering of several pathways to enable the generation of new-to-nature fluoro- and chloro-metabolites has been previously achieved^8, 15–18^. The C–F bond does not facilitate subsequent cross-coupling and the poor reactivity of C–Cl renders cross-coupling challenging as it requires harsher conditions^8^. Engineering the biosynthesis of brominated natural products represents a needed compromise between chemical reactivity and genetic install-ability. For our model system, we choose to focus toward diversification of tryptophan **1**. This amino acid is a key component in many natural products and its presence in proteins and peptides is important to their structural integrity and fluorescent properties, its modulation is therefore attractive. Mild approaches to address the functionalization of halo-tryptophans are challenging as halo-indoles are shown to be less reactive than analogous heterocyclic aryl halides^19^.

## Results

### Aqueous method for mild cross-coupling of halo-indoles

**Fig. 1:**
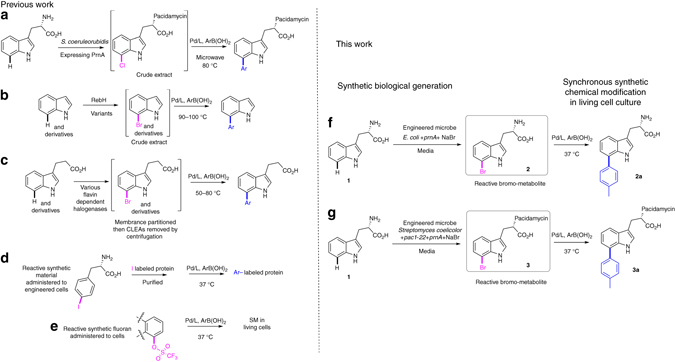
Biosynthetic halogenation enabling Suzuki–Miyaura cross-coupling. **a** in vivo generation of Cl-pacidamycin, by the natural producer, transformed with a halogenase, followed by in vitro cross-coupling of the compound as a component of cell free extract, at 80 °C (in vivo: in vitro)^8^, **b** in vitro generation of reactive bromo-aromatics by RebH variant enzymes, followed by in vitro cross-coupling of the compound as a component of the crude extract (in vitro: in vitro)^11^, **c** in vitro generation of reactive bromo-aromatics by stabilized crosslinked enzyme aggregates ((CLEAs) of a series of flavin-dependent halogenases, membrane partitioned from anaerobic, palladium-mediated cross-coupling conditions (in vitro: in vitro)^12^, **d** in vivo incorporation of reactive, synthetic iodophenylalanine into peptides, followed by protein purification and cross-coupling of the iodinated protein (in vivo: in vitro)^13^, **e** in vivo Suzuki–Miyaura modification of synthetically generated, reactive triflate fluoran (synthetic: in vitro)^14^, **f** in vivo *g*eneration of reactive 7-Br-tryptophan **2** by engineered *E. coli* RG-1500 synchronous with the in-culture Suzuki–Miyaura cross-coupling of this reactive metabolite (in vivo: in vivo); and **g** generation of reactive Br-pacidamycin D **3** by engineered *Streptomyces coelicolor* RG-1104 concomitant with the in-culture Suzuki–Miyaura cross-coupling of this reactive metabolite (in vivo: in vivo)

**Fig. 2:**
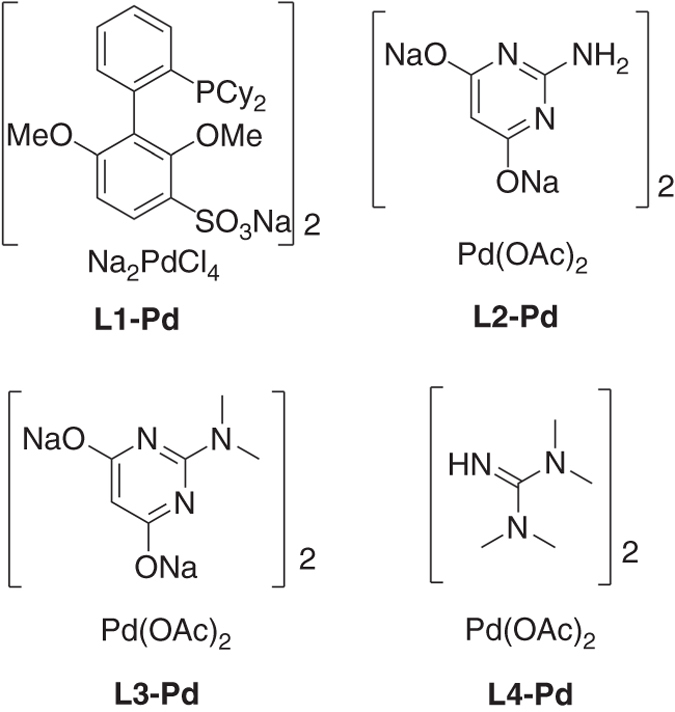
Selected water-soluble catalysts explored in this study. Catalyst stock solutions were prepared in deionized water, stored at ambient temperature and used within two weeks. **L1**-**Pd** solution were freshly prepared each day.

To work toward developing suitably mild and potentially cell compatible, aqueous and aerobic conditions for Br-tryptophan diversification (Fig. 1), preferably at 37 °C, we first explored cross-coupling of Br-indoles. The cross-coupling of 5-Cl-indole **6** at 40 °C in degassed dioxane-water using K_3_PO_4_ has precedent^20^; however, for potential biocompatibility, a more appropriate solvent and atmosphere are requisites. Starting with our model reaction of coupling of 5-I-, Br- and Cl-indole (**4**, **5** and **6**, respectively) with *p*-tolyl-boronic acid (*p*-Tol-B(OH)_2_), we screened series of sulphonated water-soluble phosphine ligand complexes (including triphenylphosphine-3,3′,3″-trisulfonic acid (TPPTS), ^*S*^SPhos [**L1-Pd**, Fig. 2], ^*S*^XPhos)^21, 22^ and pre-catalysts based on bidentate ligands^23^ such as [PdCl_2_(dppf)], [PdCl_2_(dtbpf)], [PdCl_2_(Xantphos)] and NHC-Pd^24^ for their ability to promote Suzuki–Miyaura cross-coupling in water and mild base (K_2_CO_3_, Na_2_CO_3_, K_3_PO_4_ or Cs_2_CO_3_) in the presence of air (Supplementary Fig. 1 and Supplementary Table 1). From this initial screen, the combination of the water-soluble Na_2_PdCl_4_ and sterically demanding, electron-rich ^*S*^SPhos ligand, with K_2_CO_3_ as base, showed greatest potential (Table 1). Notably, in the case of 5-I-indole **4**, we observed that its cross-coupling can be conducted in water alone, at 20 °C with a relatively low Pd-catalyst loading (2 mol%) and 1.2 equiv. *p*-Tol-B(OH)_2_, resulting in 72% nuclear magnetic resonance (NMR) yield after 8 h. Moreover, we demonstrated that addition of biologically tolerable 20% *v/v* CH_3_CN or EtOH assisted in solvating the reactants and improved the NMR yields (90 and 92% respectively at 20 °C, 8 h, Supplementary Table 1, entries 2 and 3) and that almost quantitative conversions could be achieved by increasing the temperature to 37 °C (98%, 8 h, Table 1, entry 1).

**Table 1:** Mild cross-coupling of halo-indoles


Entry	X (compound number)	Time (h)	Conversion^a^	Yield^b^
1^c^	5-I (**4**)	8	98	85 (**4a**)
2	5-Br (**5**)	12	72	—
3	5-Br (**5**)	18	99	92 (**5a**)
4	5-Cl (**6**)	19	52	—
5	5-Cl (**6**)	48	94	90 (**6a**)
6	7-Br (**7**)	20	Quant.	96 (**7a**)
7	7-Cl (**8**)	36	95	85 (**8a**)

Optimized reaction conditions for various halo-indoles

^a^Conversion was determined by ^1^H NMR of the crude reaction

^b^Isolated yields are reported after flash chromatography

^c^Reaction was carried using 2 mol% of catalyst loading and 1.2 equiv. of *p*-Tol-B(OH)_2_

Having developed very mild conditions for the cross-coupling of **4**, we investigated the cross-coupling of the less reactive and more demanding Br- and Cl-indoles. By increasing the Pd loading and boronic acid equivalents (5 mol% and 1.5 equiv. *p*-Tol-B(OH)_2_, respectively) high conversions for 5-Br- and 5-Cl-indole (**5** and **6**, respectively) were obtained at 37 °C after 18 and 48 h, respectively (Table 1, entries 3 and 5).

### Tryptophan inhibits L1-Pd catalyzed Suzuki–Miyaura coupling

**Fig. 3:**
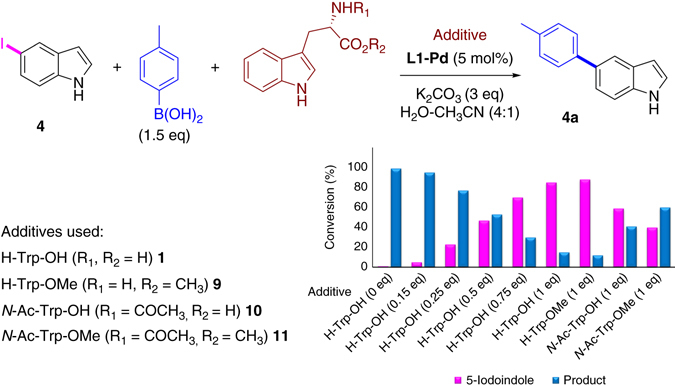
Tryptophan inhibits **L1-Pd** catalyzed cross-coupling. Inhibition of cross-coupling of 5-I-indole **4** doped with increasing concentrations of unprotected tryptophan **1** (0–1 equiv.) or selectively protected tryptophans **9–11** (1 equiv.). Conversion was determined by ^1^H NMR of the crude reaction

We next investigated the cross-coupling of halo-tryptophan using mild, aqueous conditions. We previously reported the first Suzuki–Miyaura cross-coupling reaction of unprotected Br- and Cl-tryptophans, these reactions required heating at 80 °C^25^. Under the optimal conditions for halo-indoles (described above), the cross-coupling of unprotected 5- or 7-Br-tryptophan (**13** and **2**, respectively) was unsuccessful, potentially due to their ability to coordinate to Pd and substrate could inhibit the catalysis under mild reaction conditions^10, 26^. Hence, to explore the inhibition of the cross-coupling of halo-indoles by the amino acid, we investigated the reaction of 5-I-indole **4**, doped with free tryptophan **1**, with *p*-Tol-B(OH)_2_. An almost linear drop in conversion correlated with the increase in added tryptophan **1** (Fig. 3) was observed. To explore whether the drop in conversion is directly attributable to the carboxylate or primary amine functionality, we added one equiv. of either tryptophan **1**, tryptophan methyl ester **9**, *N*-acetyl tryptophan **10** (acylated primary amine) or the *N-*acetyl tryptophan methyl ester **11** (Fig. 3). The effect of addition of tryptophan methyl ester **9** or free tryptophan **1** on the reaction were comparable (conversions reduced to 12 and 15% respectively). However, in reactions where one equiv. of *N*-acetyl tryptophan **10** was added, as a potential inhibitor, a 41% conversion resulted. This indicates that the primary amine of tryptophan plays a key role in the inhibition of the reaction; however, the carboxylate may not be considered as completely innocent as when doubly protected tryptophan **11** was added, a conversion of 59% resulted under the same reaction conditions.

It is possible, therefore, that *N-* and *O*-coordination complexes (NH_2_-Pd, COO-Pd) or *N,O-*Pd may be formed *in situ*, reducing the effective concentration of available Pd for catalysis^26–29^. Usefully, we observed that inhibition of cross-coupling by tryptophan **1** can be partially reversed by increasing the concentration of *p*-Tol-B(OH)_2_. These results indicate that chelation of Pd by the amino acid motif is not the sole possibility; but that heterodimeric complexation of amino acid with boronic acid is also present^30^. Together, these two equilibria could contribute towards inhibition of the cross-coupling reaction.

### Suzuki–Miyaura coupling of Br-tryptophans and tripeptides

**Fig. 4:**
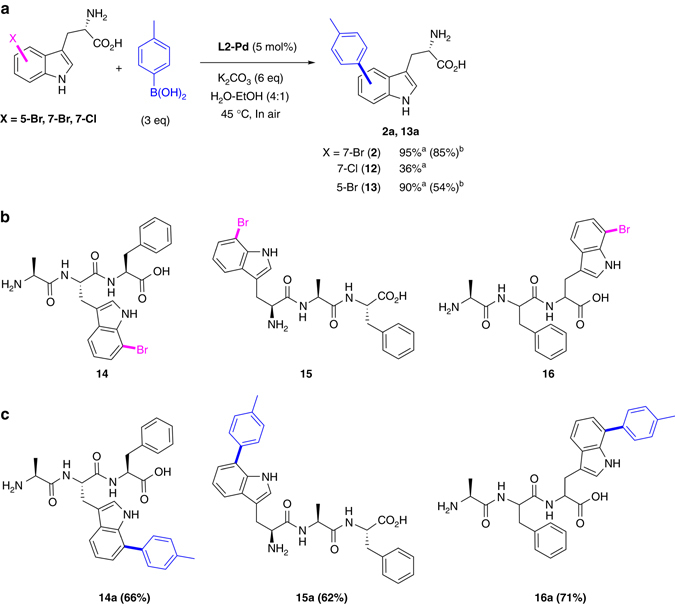
Mild cross-coupling of Br-tryptophans and tripeptides. **a** Mild cross-coupling of unprotected halo-tryptophans at 45 °C using **L2-Pd**, ^a^conversion is determined by ^1^H NMR of the crude reaction, ^b^isolated yield after purification by reversed-phase chromatography. **b** synthetic tripeptides incorporating 7-Br-tryptophan **2** used for cross-coupling studies, **c** Suzuki–Miyaura cross-coupling products derived from the corresponding tripeptides containing 7-Br-tryptophan. A mixture of Br-tryptophan or bromo-tripeptide, **L2**-**Pd** (5 mol%) catalyst, *p-*Tol-B(OH)_2_ (3 equiv.) and K_2_CO_3_ (6 equiv.) in water-EtOH (4:1) or water was stirred at 45 °C for 48 h. Isolated yields are reported after purification by reversed-phase chromatography

In the light of our observations on the inhibitory effect of the amino acid, we next set out to explore mild conditions that could be translated to cross-coupling of unprotected halo-tryptophan or halo-tryptophan containing peptide. We screened series of catalysts and ligands for any that would provide reactivity at physiological temperature (Supplementary Fig. 1). Recently, Ballet et al.^31^. reported that the palladium pre-catalyst of disodium 2-aminopyrimidine-4,6-diol^31–34^ (**L2-Pd**, Fig. 2) was effective for cross-coupling of halo-tryptophans at 80 °C under microwave irradiation. We further explored the utility of **L2-Pd** to perform cross-coupling at lower temperature. After optimizing reagent concentrations (3 equiv. *p*-Tol-B(OH)_2_ and 6 equiv. K_2_CO_3_), we successfully cross-coupled unprotected 7-Br-tryptophan **2**, at 45 °C (Fig. 4a). The reaction proceeded smoothly using 5 mol% of the **L2-Pd** catalyst and the product 7-*p-*tolyl-tryptophan **2a** was isolated in high yield (85%). For 5-Br-tryptophan **13**, a 90% NMR conversion and a lower 54% isolated yield of the product **13a**, potentially due to product solubility issues, was observed. Moreover, a modest 32% conversion was observed for the significantly more challenging and less reactive unprotected 7-Cl-tryptophan **12** (Fig. 4a).

To assess the applicability of these cross-coupling conditions for the modification of small peptides, we explored the reaction with three tripeptides containing 7-Br-tryptophan **2** placed centrally **14** or at the *N*-terminal **15** or *C*-terminal **16** (Fig. 4b)^35^ (for syntheses, see Supplementary Methods). The reactions worked well in aqueous conditions without need for organic co-solvent and at relatively low catalyst loading (**L2-Pd**, 5 mol%). The cross-coupling products **14a-16a** were isolated in good yields (62–71%, Fig. 4c). In general, this mild protocol is amenable for derivatisation of peptides and has potential to be applicable to higher polypetides/proteins possessing halo-aryl functionality.

Our ultimate and ambitious goal was to, for the first time, enable engineered production of a halogen tagged metabolite and its cross-coupling in living culture. This would be a significant breakthrough in the field. Such a combination of genetic engineering to generate tagged metabolites and both synchronous and sequential chemical modification of the tag has the exciting potential to pave the way to in vivo diversification, labeling and affinity tagging. To explore whether we could engineer biosynthetic halogenation and chemical cross-coupling in a living system, we chose to work toward two model systems of varying challenge: firstly, engineering *Escherichia coli* for biosynthesis of 7-Br-tryptophan **2**, secondly engineering streptomycetes to produce Br-pacidamycin D. Pacidamycin represents a challenging model system; its selective functionalization is inherently difficult as it contains both a peptide chain and a modified nucleic acid moiety. Pacidamycin is produced, like many natural products, in very low concentration and challenges any synthetic methodology to be efficient even at such high dilution. It also exemplifies a further demand in accessing the bromo-metabolite because the natural producer has poor tolerance to bromide, and the biosynthetic gene cluster would therefore need to be introduced into a more suitable host strain. We set out to engineer the two systems and to systematically and iteratively address the impact of the cross-coupling reagents on the two biological systems and assess how components from the fermentation media would impact upon cross-coupling efficiency. To undertake this ambitious task, we first explored and enabled the generation and cross-coupling of 7-Br-tryptophan **2** in *E. coli* culture.

### Applying in-culture GenoChemetics to tryptophan

A plasmid carrying *prnA* encoding a tryptophan 7-halogenase from pyrrolnitrin biosynthesis^36^ was constructed and introduced into *E. coli* PHL644 strain (engineered *E. coli* RG-1500), and upon supplementation of the culture with sodium bromide, resulted in production of 7-Br-tryptophan **2** (see Supplementary Figs 6–8 and Supplementary Methods for details).

We set out to establish conditions that would promote 7-Br-tryptophan **2** production and be suitable for its corresponding cross-coupling in living cultures of *E. coli*. While cross-coupling of synthetic halogenated materials has been previously reported in buffer^37^ there are no reports of reactions carried out in media that would be required to sustain growth and metabolite production. In parallel, we established the effect of Pd, ligand and boronic acid concentration, both separately and in combination, on *E. coli* viability; and explored the efficiency of cross-coupling of 7-Br-tryptophan **2** within a range of media and in the presence of individual media components. As an in vitro model reaction for this screening, we used 7-Br-tryptophan **2** (0.35 mM)^38^, *p*-Tol-B(OH)_2_ (1.2 mM, 3.4 equiv.) and **L2-Pd** catalyst (0.1 mM) (for details of the media screening see Supplementary Discussion). In summary, key observations revealed that only trace conversion ( < 2%) occurred in the presence of series of standard culture media (e.g., Luria-Bertani (LB), 2xYT) or yeast/malt extracts (Supplementary Table 2). However, we observed that cross-coupling conditions were tolerant to phosphate buffer (100 mM with 50 mM NaCl/Br, pH 8) and citrate buffer (100 mM, pH 8). Provision of both a carbon and nitrogen source is essential for bacteria growth. Nitrogen is usually supplied from amino acids and/or ammonium salts in the media. Our investigations demonstrated both the free amine and carboxylate of tryptophan **1** had a detrimental effect on the reaction (discussed in earlier section). Unsurprisingly, cross-coupling was strongly suppressed by the presence of amino acids (e.g., glycine 20 mM) as well as ammonium salts. Provision of a suitable carbon source could also prove a challenge, as poor conversions (~10%) were observed in the presence of glucose (22 mM, 0.4% *w/v*) or glycerol (217 mM, 2% *w/v*). Alternative carbon sources, that could either enter directly into the Krebs’ cycle (such as citrate) or could be slowly metabolized so that inhibitory monosaccharide and glycerol concentrations would remain low, were also explored (see Supplementary Table 2).

Following numerous iterative rounds of assessing media that would enable bacterial growth, 7-Br-tryptophan **2** production and be cross-coupling compatible, we determined that a minimal media containing potassium nitrate (20 mM) as the nitrogen source, glycerol (0.5% *w/v*, 55 mM) as the sole carbon source and supplemented with 50 mM NaBr (‘cross-coupling media’ (CCM)) could be employed. Aware that the bacteria would metabolize the media, thereby depleting the carbon and nitrogen sources initially supplied, we also considered that a dynamic living system would generate cofactors, bio-thiols and radical species with a potential to poison Pd-catalyst and be detrimental to the cross-coupling reactions. To determine the impact of such metabolites, cultures of the engineered *E. coli* RG-1500 were grown and bromination of tryptophan was performed; cells were then removed by centrifugation and the resultant cell free supernatant media containing in vivo generated 7-Br-tryptophan **2** (‘spent media’) was used for further screening of the cross-coupling reactions. Though series of metabolites that have the potential to interfere with the catalytic cycle, chelating to the Pd have been generated, encouragingly 82% conversion for the cross-coupling could be achieved, if first diluted 1:5 in phosphate buffer (Supplementary Table 4). The reactions in the spent CCM proceeded well to give high conversions; however, the reaction time was very long (60 h) upon using *p*-Tol-B(OH)_2_ (1.2 mM) and **L2-Pd** (100 µM). Thus, we again looked further toward a more active Pd catalyst system. Pd-catalysts based on tetramethylguanidine (**L4-Pd**)^39^ and 2-(dimethylamino)-pyrimidine-4,6-diol (**L3-Pd**)^40^ ligands were recently reported to enable the expeditious cross-coupling of activated aryl iodides. Importantly, both are water-soluble and their lack of toxicity is attractive for in vivo applications. We were pleased to find both ligand variants (**L3-Pd**, **L4-Pd**) afforded increased catalytic activity with ~2-fold increase in conversions (75% and 70% respectively) over **L2-Pd** catalyst (38%) using the spent CCM (Supplementary Fig. 3). Moreover, these conversions were obtained after 18 h using 100 µM Pd-catalyst and 1.2 mM boronic acid at 1:4 dilution. A time course experiment at 1:8 dilution revealed complete conversion of **2** within 4 h and high Ultra Performance Liquid Chromatography (UPLC) yields of cross-coupling product for both **L3-Pd** (76%) and **L4-Pd** (64%) were obtained for the spent CCM (Supplementary Fig. 4). These results, achieved at a low Pd-concentration (50 µM) within 4 h, represented significant advancement towards our goal.

**Fig. 5:**
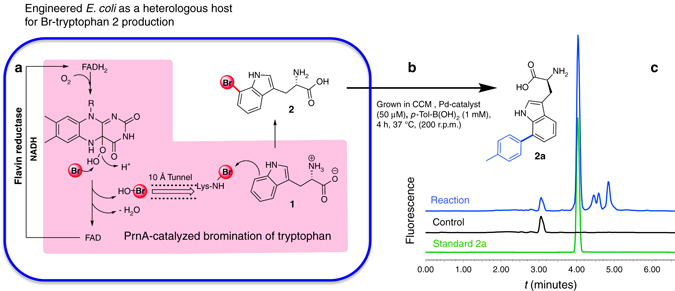
Synchronous production and cross-coupling of 7-Br-trytpophan. **a** *E. coli* PHL644 engineered to produce tryptophan 7-halogenase (prnA) (*E. coli* RG-1500). **b** Cross-coupling of the 7-Br-tryptophan **2** produced in living culture. An approximate conversion of 61% can be calculated based on the levels of the cross-coupled product **2a** observed and based on the concentration of 7-Br-tryptophan **2** at the start of cross-coupling; however, as 7-Br-tryptophan **2** can be constantly produced and metabolized by living cultures, this can only be an approximation. **c** UPLC analysis, fluorescence chromatograms of the cross-coupling reaction (*top trace*), a control culture without Pd-catalyst or boronic acid (*middle*) and a purified standard of product **2a** (*bottom trace*)

Having developed a suitable medium and improved catalytic system, we cultured *E*. *coli* RG-1500 in CCM supplemented with L-tryptophan (1.5 mM). By UPLC, we determined that optimal 7-Br-tryptophan **2** production was achieved after 24 h (see Supplementary Fig. 7–9); at this time point, the culture was mixed with phosphate buffer (10 mM, pH 8.5), followed by addition of *p-*Tol-B(OH)_2_ (1 mM) and appropriate Pd-catalyst (50 µM) and incubated for a further 4 h to obtain a conversion of 61% of the desired product **2a** (Fig. 5 and Supplementary Table 5). The slightly lower conversions of the product **2a** in a matured living culture may be due to interference from the soluble cofactors required for halogenation as catalyzed by PrnA (e.g., redox active NAD^+^/NADH system), excreted amino acids/proteins (coordinating to Pd), carbohydrates (chelation with boronic acid) as well as bio-thiols (Pd-poisoning). The viability of cells was assessed by comparing the numbers of colony forming units. The cells from the in-culture cross-coupling remained fully viable (Supplementary Fig. 12).

### Antibiotic analogue generation via in-culture GenoChemetics

The antibiotic pacidamycin is a challenging system demanding that any orthogonal derivatization chemistry developed must be nucleoside and peptide compatible. We previously demonstrated the first out of context use of a halogenase, introducing the gene encoding prnA to the wild-type pacidamycin producing strain *Streptomyces coeruleorubidis* in order to generate a strain (RG-5059) capable of producing chloropacidamycin^8^.

Generation of the more reactive bromo-metabolite would be essential if we were to achieve compound generation and synthetic modification synchronously in live culture. However, supplementation of RG5059 culture media with various bromide salts including KBr, NaBr, NH_4_Br impaired culture growth and no brominated pacidamycin could be detected; it was clear that the natural pacidamycin producer could not be utilized for the generation of brominated metabolites. We investigated the tolerance of another streptomycete, *S. coelicolor* M1154^41^, to bromide salts (from 0 to 200 mM in 50 mM increments) and determined 100 mM KBr to be well tolerated. Next, we engineered the heterologous production of pacidamycin into this strain by conjugatively transforming the strain with *S. coeruleorubidus* cosmid 2H-5^42^, which carries *pac1*-*pac22* as a 32.2 kb insert. This new synthetic strain (named RG-4242) was shown to produce pacidamycin, and its growth and production of pacidamycin not be compromised by bromide salts. Next, we installed *prnA*, encoding tryptophan 7-halogenase, into the genome (see Supplementary Methods and Supplementary Figs 13–15), resulting in engineered strain RG-1104. Cultures of RG-1104 grown in the presence of sodium bromide (50/100 mM) were demonstrated to produce Br-pacidamycin D **3** by liquid chromatography-mass spectrometry (LC-MS; Supplementary Figs 19–21).

**Fig. 6:**
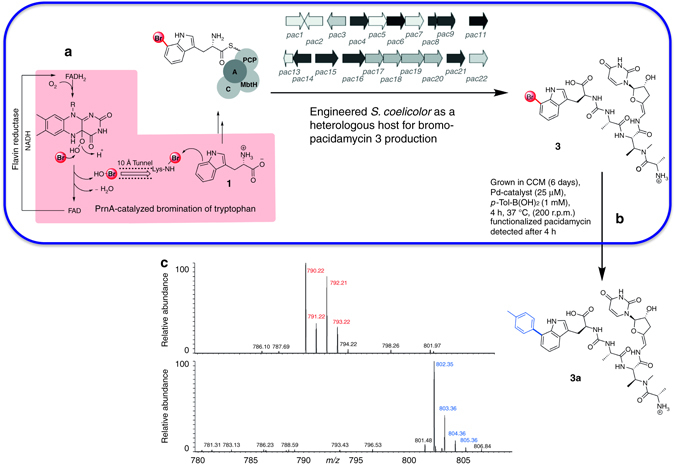
In culture biosynthesis and cross-coupling of Br-pacidamycin-D. **a** *S. coelicolor* M1154 engineered to express the tryptophan 7-halogenase gene (*prnA*) and the entire pacidamycin gene cluster (*pac1–pac22*), which encodes the non-ribosomal peptide template/thiol tethered biosynthesis of pacidamycin (*S. coelicolor* RG-1104). **b** Cross-coupling of the Br-pacidamycin D **3** produced by the living culture. **c** Extracted Ion Chromatograms of Br-pacidamycin D **3** and the cross-coupled product *p-*tolyl-pacidamycin D **3a**. This system exemplifies that this chemistry is not only compatible with less robust living cells but also that it is compatible with the very high dilutions and low titers that many natural products are present at. The engineering of bromo-metabolite production enables this exciting advance. Furthermore, the use of this model system exemplifies that the chemistry is compatible with both peptides and nucleosides

Having utilized a synthetic biological approach to generate a strain capable of producing new-to-nature bromo-metabolites, the stage was set to determine whether synchronous biosynthetic bromo-natural product production and chemical diversification in living bacterial culture could be accomplished. This system further challenged the limits of the technology with the *S. coelicolor* culture being considerably more susceptible to palladium poisoning than the *E. coli* culture and the metabolic profile being rather more complex. Exploring the media (CCM) that was developed for the cross-coupling of **2** (Fig. 5) in living cultures of *E. coli*, we observed that it was both capable of supporting the growth of *S. coelicolor* (RG-1104) and production of Br-pacidamycin D **3** (Fig. 6). To implement the cross-coupling in the presence of the living cells, a mature (6 day old) culture grown in CCM was mixed with phosphate buffer, followed by addition of *p-*Tol-B(OH)_2_ and appropriate **L3-Pd/L4-Pd** solutions and incubated at 37 °C, 25 μM Palladium and 1 mM boronic acid proved to be optimal. The reaction mixture was analyzed after 4 h by LC-MS, revealing the complete consumption of Br-pacidamycin-D **3** and presence of the desired product *p*-tolyl-pacidamycin D **3a** (Fig. 6 and Supplementary Figs 23–25). Again, we explored the viability of the engineered cells following the reaction and quenching with DTT at 4 h, little impact on the cell viability could be observed for **L4-Pd** ( > 96% viability Supplementary Fig. 26).

## Discussion

In summary, we have developed, in two different systems, the first synthetic biological generation of new-to-nature bromo-metabolites and their synchronous cross-coupling within a living aerated microbial fermentation, even at high dilution. As many natural products are generated at low concentration, this is essential. This pioneering approach and exceptionally mild conditions for Suzuki–Miyaura cross-coupling of challenging halo-metabolites have the potential to be broadly used for the GenoChemetic diversification of halogenated natural products and new-to-nature halo-natural products. This approach is powerful as it enables the generation and diversification of a natural product in the presence of the cells that produce it. Being able to perform this chemistry in the presence of living cells at 28 or 37 °C, including on as challenging a system as free halo-tryptophan, opens up new possibilities, for example: assessing and screening, in vivo, the directed evolution of the biosynthetic enzymes like halogenases or multifunctional enzymes for example polyketide synthases (PKSs) and nonribosomal peptide synthetases (NRPSs). In such directed evolution studies, being able to assay in vivo is time efficient and maintaining the viability of the cells removes need for duplicate plating—a time consuming process when handling the large numbers of colonies required for such systems. Recently, Sewald reported the use of the fluorescence modulating cross-coupling chemistry, which we have previously developed^25^, as a screen for the directed evolution of a thermophilic halogenase^43^. The cell compatible chemistry developed here has the potential to significantly open up this area, making this approach applicable to aryl halogenases that do not originate from thermophiles. One might also envisage utilizing the combination of synthetic biology and synthetic chemistry, which results in a change in fluorescence to enable cell sorting of particularly productive clones^25^. A further advantage of carrying out the chemistry in the presence of the living cells is that, with the provision of additional media, the cells can continuously produce their halo-metabolites, such an approach could potentially be applied to biofilms in flow, or possibly have dual utility in diversification and increasing metabolite flux^44^. From a technical viewpoint the ability to carry out the diversification without an additional purification step, in the presence of a continuously producing biosynthetic system is attractive. Furthermore, we find that the enhanced lipophilicity that the cross-coupling diversification confers, significantly simplifies the otherwise challenging extraction and purification from other media components.

Being able to use synthetic biology to generate new-to-nature halo-natural products and carry out in-culture diversification of these metabolites also paves the way for chemical biological studies in which the interactions of the diversified natural product can be explored in the producing organisms, affinity or fluorescence labeling of microbial natural products and their subsequent tracking. For example, it would be interesting to see if this could be applied to determine the origin and distribution of halogenated signaling molecules. In the light of the potential power and applicability of this approach, it is our aim to expand the toolkit of potential orthogonal chemistries that can be translated into the living GenoChemetic context in order to provide new tools to monitor and better understand biological processes or manipulating natural product targets in a precise manner.

## Methods

### General information

All chemical reagents were used as received from commercial sources without further purification or prepared as described in the literature. The fully synthetic reactions were monitored by thin-layer chromatography and the products were purified using flash chromatography and characterized using NMR and mass spectrometry. For experimental details and characterization of all new compounds (^1^H/^13^C NMR and MS, see Supplementary Methods and Supplementary Figs 27–50). The reactions carried out in cell culture and involving the synthetic-biological/synthetic-chemical generation of materials in 1 µM-500 µM quantities were followed by UPLC and liquid chromatography-tandem mass spectrometry and compared to known standards. Additional methods for all synthetic/biosynthetic preparations and associated references are available in the Supplementary Methods.

### Cross-coupling of halo-indoles using L1-Pd catalyst

In a screw cap glass vial, appropriate halo-indole (0.1 mmol), *p-*Tol-B(OH)_2_ (20 mg, 0.15 mmol), potassium carbonate (42 mg, 0.3 mmol) were suspended in water-CH_3_CN mixture (4:1, 1.8 ml). A solution of **L1-Pd** in water (5 mol%, 0.2 ml) was added. The vial was closed and stirred at 37 °C until reaction completion. Reaction was diluted with brine (2 ml) and extracted with ethyl acetate (3 × 3–4 ml). Combined organic extract was dried (MgSO_4_), filtered and solvent removed under reduced pressure. The desired product was purified by flash column chromatography.

### Cross-coupling of halo-tryptophans and tripeptides using L2-Pd catalyst

In a screw cap glass vial, appropriate 5- or 7-Br-tryptophan (**13** and **2**, respectively, 14 mg, 0.05 mmol), *p-*Tol-B(OH)_2_ (20 mg, 0.15 mmol) and potassium carbonate (41 mg, 0.3 mmol) were suspended in water-EtOH mixture (4:1, 1.0 ml). A solution of **L2-Pd** (5 mol%) in water was added. The vial was closed and stirred at 45 °C for 48 h. The reaction was diluted with water (2 ml) and extracted with diethyl ether (3 × 2 ml). The aqueous layer was acidified (pH ~2–3) using 0.1 M HCl. Solvent was removed under reduced pressure. The desired product was obtained by purification using gradient reversed phase chromatography (C-18, 12 g) eluting with water-MeOH (5–95% gradient). Reactions for tripeptides were conducted using only water as solvent.

### Cross-coupling of 7-Br-tryptophan or Br-pacidamycin D in living cultures

Appropriate culture of engineered *E. coli* RG-1500 or *S. coelicolor* RG-1104 containing either 7-Br-tryptophan **2** or Br-pacidamycin D **3** (5 ml) was diluted with phosphate buffer (45 ml, 10 mM, pH 8.5) in a sterile 250 ml flask. Filter sterilized solutions of *p-*tol-B(OH)_2_ (final concentration 1 mM, 0.5 ml of 100 mM stock in 90% aqueous ethanol) and appropriate Pd-catalyst (final concentration 50 or 25 µM, 250 or 125 µl of 10 mM stock in water of **L3-Pd** or **L4-Pd**) were added. Culture flasks were incubated at 37 °C (200 r.p.m., incubator throw 19 mm) for 4 h. Cross-coupling was quenched by addition of DL-dithiothreitol (DTT, 25 µl of 1 M stock in water). An aliquot (1 ml) was collected, cells were lysed by 5 freeze-thaw cycles and debris removed by centrifugation (13,000 r.p.m., 16,060 × *g*, 5 min). Clear supernatant was collected and analyzed by UPLC/LC-MS.

### Fluorescence quantification of 7-(*p-*tolyl)-tryptophan 2a (UPLC method 1)

UPLC analysis was performed on Phenomenex Kinetex phenyl-hexyl column (2.1 μm, 2.1 × 75 mm) column eluting with 0.1% trifluoroacetic acid (TFA) in water (solvent A) and methanol (solvent B). The following gradient was used: 0–0.2 min (5% B), 0.2–5.0 min (5–95% B), 5.0–5.5 min (95% B), 5.5–5.6 (95–5% B), 5.6–6.6 (5% B). The flow rate was set to 500 μl min^−1^ and the column temperature was maintained at 50 °C. Detection was achieved by UV absorbance (Photodiode Array Detector (PDA) 200–400 nm, UV 280 nm). Fluorescence emission was detected using an excitation wavelength of 295 nm and emission wavelength of 370 nm (gain × 1). The calibration curve for 7-(*p*-tolyl)-tryptophan **2a**, linear in the 0.78–12.5 µM concentration range, was used for quantification (Supplementary Fig. 2).

### Quantification of 7-Br-tryptophan (UPLC method 2)

UPLC analysis was performed on Acquity UPLC BEH C18 (1.7 µm, 2.1 × 50 mm) column eluting with 0.1% TFA in water (solvent A) and acetonitrile (solvent B). Following gradient was used: 0–0.5 min (5% B), 0.5–1.5 min (5% to 15% B), 1.5–9.0 min (15% B), 9.0–10.0 min (15% to 95% B), 10.0–11.0 min (95% B), 11.0–12.0 (95–5% B), 12.0–16.0 (5% B). The flow rate was set to 260 μl min^−1^ and the column temperature was maintained at 40 °C. Detection was achieved by UV detection (PDA 200–400 nm, UV 280 nm). The calibration curve for 7-Br-tryptophan was linear in 15.6–500 µM concentration range (Supplementary Fig. 9) and was used for quantification.

### LC-HRMS analysis

Liquid chromatography-high resolution mass spectrometry (LC-HRMS) analysis of the cross-coupling reactions was conducted on a Thermo Scientific Dionex Ultimate 3000 Rapid Separation LC system using XBridge BEH C18 column (130 Å, 3.5 µm, 2.1 × 100 mm). The flow rate was set to 0.35 ml min^−1^ and the column temperature was maintained at 40 °C. A generic binary gradient elution was carried out using different ratios of eluents A (water containing 0.1% formic acid) and B (acetonitrile). Following gradient was used: 0–0.5 min (5% B), 0.5–9.5 min (5% to 95% B), 9.5–11.5 min (95% B), 11.5–12.0 min (95% to 5% B) and 12.0–15.0 min (5% B). The mass spectrometric analysis was performed on an Orbitrap Velos Pro™ mass spectrometer system equipped with a Thermo Scientific Ion MAX API source housing. The MS conditions were as follows: heated electrospray ionization (HESI-II) probe, positive ionization mode, spray voltage 3.5 kV, capillary temperature 350 °C, normalized collision energy 35% for collision induced dissociation, and sheath gas and auxiliary gas flow rates of 35 and 10 arbitrary units, respectively. Full scan MS spectra (from *m/z* 100–1000) were acquired in the orbitrap with resolution *R* = 60 K.

### Production of 7-Br-tryptophan 2 by engineered *E. coli* RG-1500

Construction of recombinant plasmid of pSG36 for expression of the inserted halogenase system composed of the tryptophan 7-halogenase gene (*prnA*) and the flavin reductase gene (*ssuE*) is given in Supplementary Fig. 6. A single colony of the *E. coli* K-12 PHL644 harboring plasmid pSG36 designated as RG-1500, from the LB agar plates was inoculated in 5 ml of LB broth supplemented with 100 μg ml^−1^ ampicillin and 50 μg ml^−1^ kanamycin. This culture was incubated for 12 h (37 °C, 200 r.p.m., incubator throw of 19 mm) and was subcultured (1:100) into 200 ml LB broth supplemented with 100 μg ml^−1^ ampicillin and 50 μg ml^−1^ kanamycin in 2 litre baffled flasks. These cultures were grown to an OD_600_ of 0.6 (37 °C, 200 r.p.m., incubator throw 19 mm). The growth temperature was then reduced to 16 °C over 30 min. After cooling, the cell cultures were aliquoted into 100 ml in 2 litre baffled flasks. Sodium propionate was then added, as inducer, at a final concentration of 20 mM (from 2 M stock in water) for inducing expression of the inserted halogenase system. A 100 ml culture, without the addition of sodium propionate, was used as a control of the induction process. The cultures were incubated for a further 24 h (16 °C, 200 r.p.m., incubator throw 19 mm). Following this incubation period the cultures were transferred aseptically into sterile 750 ml polypropylene centrifuge bottles (Beckman Coulter UK Ltd.). Cells from each 100 ml culture, were pelleted to remove LB growth media by centrifugation (8000 r.p.m., 15,970 × *g*, 10 min, 16 °C), washed twice with 100 ml of 10 mM phosphate buffer (pH 8.5) and re-suspended in 45 ml CCM. Each 45 ml of cell suspension was split into 15 ml aliquots, and 1.5 mM of L-tryptophan was added (i.e., addition of 957 μl of a 25 mM stock in 10 mM phosphate buffer (pH 8.5) to each 15 ml of cell suspension). The cultures were incubated for 24 h (28 °C, 200 r.p.m., incubator throw 19 mm) to enable the generation of 7-Br-tryptophan **2**.

### Br-pacidamycin production by engineered *S. coelicolor* RG-1104

PCR-targeting^45^ was used to replace the kanamycin resistance gene on cosmid 2H-5^42^ containing the pacidamycin biosynthetic genes (*pac1-pac22*) with a cassette for conjugal transfer and site-specific integration into Streptomyces. The integration cosmid was introduced into *S. coelicolor* M1154 by conjugal transfer from *E. coli* ET12567/pUZ8002 to generate strain RG-4242, which had the expected apramycin-resistant, kanamycin-sensitive phenotype consistent with integration of the pacidamycin genes into the genome. Finally, plasmid pSG19, containing the halogenase gene (*prnA*), was introduced into *E. coli* ET12567 carrying pUZ8002 before transfer to RG-4242 by conjugation, as previously described for *S*. *coeruleorubidus* ^8^. The resulting strain, designated as RG-1104, was used for production of brominated pacidamycins (for details, see Supplementary Methods and Supplementary Figs 13–20).

### Streptomycetes culturing for metabolite production

Spore stocks of the streptomycetes to be cultured (40 µl) were used to inoculate starter culture (5 ml) containing appropriate antibiotics. The cultures were incubated for 20 h (28 °C, 180–220 r.p.m., incubator throw 2.54 cm) before 10-fold dilution into fresh starter media containing antibiotics. The cultures were incubated for a further 20 h (28 °C, 180–220 r.p.m., incubator throw 2.54 cm). The starter cultures were then used to inoculate the main culture (plus antibiotics, 20-fold dilution) which were incubated for 5–8 days (28 °C, 180–220 r.p.m., incubator throw 19 mm). Starter cultures were grown in sterile baffled flasks (250 ml) whilst main cultures were grown in sterile 250 ml conical flasks (for 30 ml cultures) or sterile 2 litre conical flasks (for 0.5 litre cultures). Sterile springs were placed inside the flasks to prevent clumping of the mycelium.

### Cell viability analysis for cultures after cross-coupling reaction

Samples from cross-coupling reactions of *E. coli* RG-1500 or *S*. *coelicolor* RG-1104 cultures, containing 7-Br-tryptophan **2** or Br-pacidamycin D **3**, treated with *p-*Tol-B(OH)_2_ (1 mM) and appropriate Pd-catalyst (50 or 25 µM, **L3-Pd** or **L4-Pd**) were incubated for 4 h (37 °C, 200 r.p.m., incubator throw 19 mm). The cross-coupling reaction for *S. coelicolor* RG-1104 cultures was quenched by addition of DTT (25 µl of 1 M stock). An aliquot (1 ml) was used for cell viability assay. The reaction culture (30 µl) was transferred to a sterile 96-well plate containing 270 μl of sterile phosphate buffer (10 mM, pH 8.5) obtaining a 10-fold diluted cell solution (1 × 10^−1^ dilution). In this fashion, a serial dilution was performed to give the final concentration 1 × 10^−6^; 100 μl of the final dilution were spread on LB-agar plates containing 50 µg ml^−1^ kanamycin or ISP2 agar plates, respectively. The plates were incubated in a static incubator at 37 °C overnight and colony forming units were counted. In parallel, this process was repeated for a control reaction culture, without treatment with both the Pd-catalyst and *p-*Tol-B(OH)_2._ The experiments were performed in triplicate (Supplementary Figs 12 and 26).

### Data availability

Data supporting the findings in this study are available within the article (and its supplementary information files) and from the corresponding author upon reasonable request.

## Electronic supplementary material

Supplementary Information
