# Supplementary material for: Living GenoChemetics by hyphenating synthetic biology and synthetic chemistry in vivo
Source: Nat Commun. 2017 Aug 9;8:229. doi: 10.1038/s41467-017-00194-3 (PMC5550429; doi:10.1038/s41467-017-00194-3)
Supplement: Supplementary file 1 — Supplementary Information [file 41467_2017_194_MOESM1_ESM.pdf]

SI GUIDE

File Name: Supplementary Information
Description: Supplementary Figures, Supplementary Tables, Supplementary Discussion, Supplementary Methods and Supplementary Methods.

SUPPLEMENTARY FIGURES

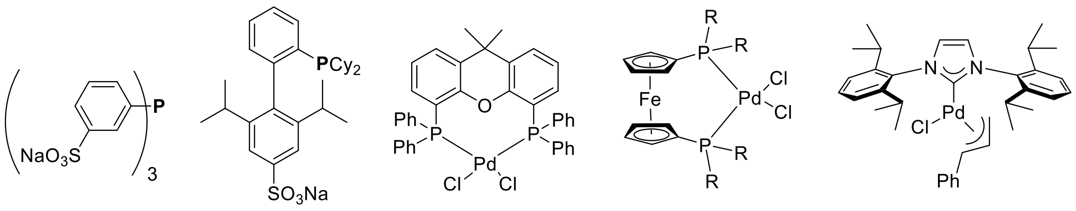

TPPTS | **^{S}XPhos** | **Xantphos.PdCl$_2$** | **PdCl$_2$(dppf) (R = Ph)** **PdCl$_2$(dtbpf) (R = ^{t}Bu)** | **[Pd(IPr)(cin)Cl]**

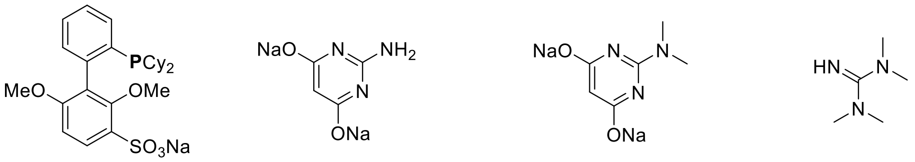

^{S}SPhos (L1) | **2-Aminopyrimidine -4,6-diol (L2)** | **2-(Dimethylamino)-pyrimidine-4,6-diol (L3)** | **1,1,3,3-Tetramethyl guanidine (L4)**

Supplementary Figure 1. Selected ligands and pre-catalysts explored in this study.

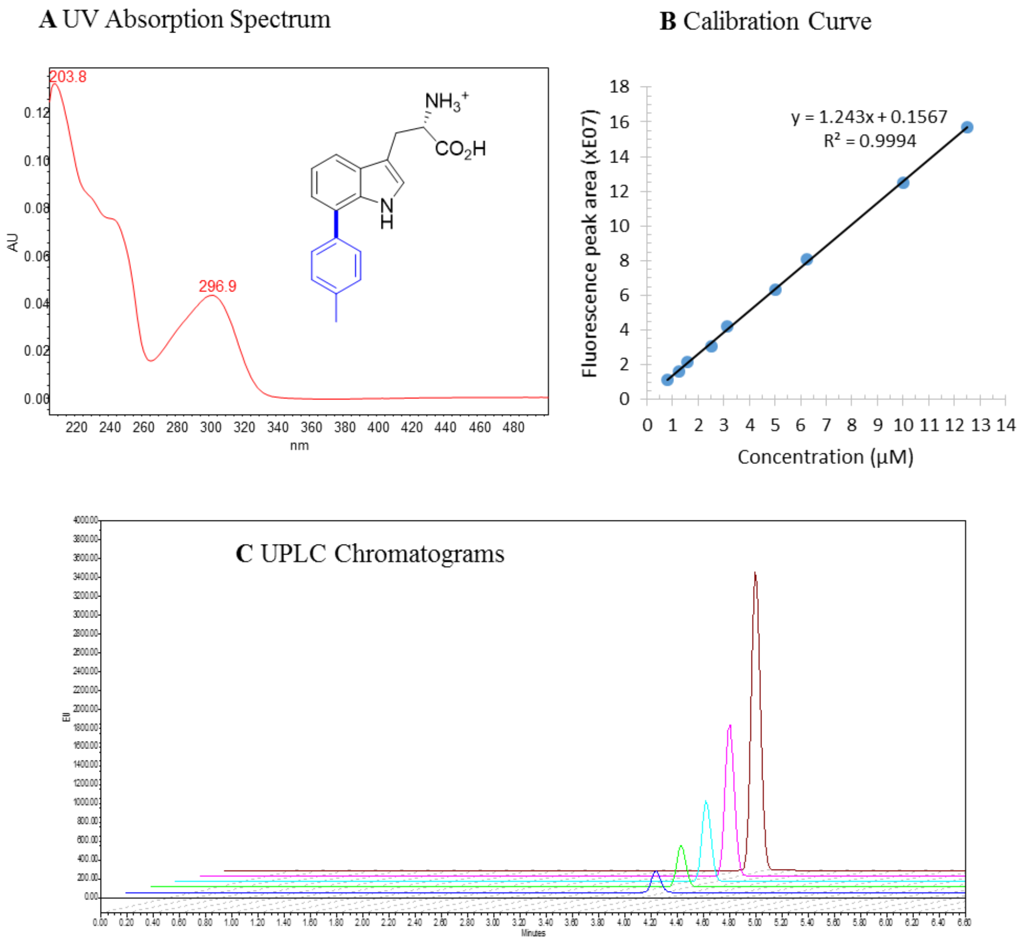

Supplementary Figure 2. UPLC analysis (method 1) of purified 7-(*p*-tolyl)-tryptophan 2a. (**A**) UV absorption spectrum of 7-(*p*-tolyl)-tryptophan **2a** (PDA detection); (**B**) Calibration curve of fluorescence peak areas measured at excitation wavelength (λ_{Ex}) 295 nm and emission wavelength (λ_{Em}) 370 nm, versus the concentration of the standard solutions ranging from 0.75 µM to 12.5 µM. This standard curve was used for determination of the concentration of the cross-coupling product from 7-Br-tryptophan **2**; (**C**) Overlay of UPLC fluorescence chromatograms for 7-(*p*-tolyl)-tryptophan **2a** with increasing concentrations (λ_{Ex} 295 nm, λ_{Em} 370 nm, retention time 4.0 min).

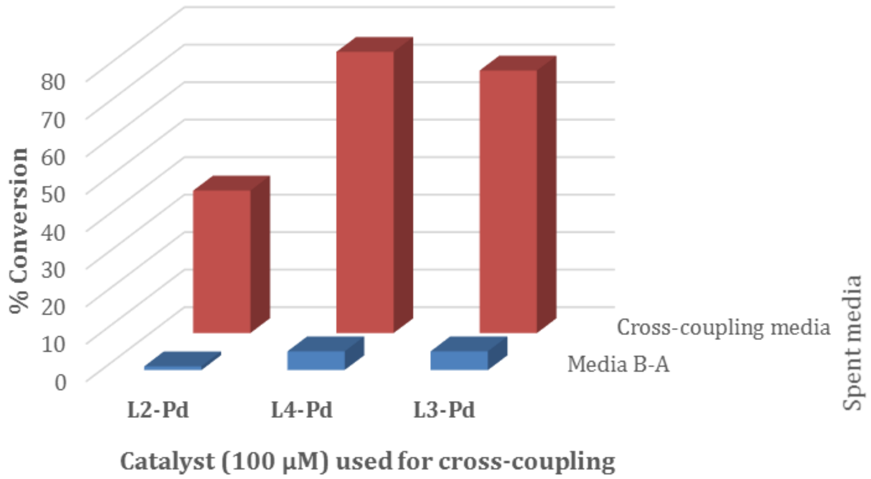

Supplementary Figure 3. Effect of different Pd-catalysts on cross-coupling of 7-Br-tryptophan 2 (*in vivo* generated by bromination of L-tryptophan 1) from spent culture media at 37 °C after 18 h. Conditions: Cell free spent media containing 7-Br-tryptophan **2** (0.5 ml), appropriate Pd-catalyst (100 µM), *p*-Tol-B$(OH)_2$ (1.2 mM) in phosphate buffer (total volume 2 ml) was stirred at 37 °C for 18 h; 7-Br-tryptophan **2** concentration in media B-A and CCM were 0.25 mM and 0.28 mM respectively. Conversion was determined by UPLC (UPLC method 1) using the product **2a** fluorescence peak area.

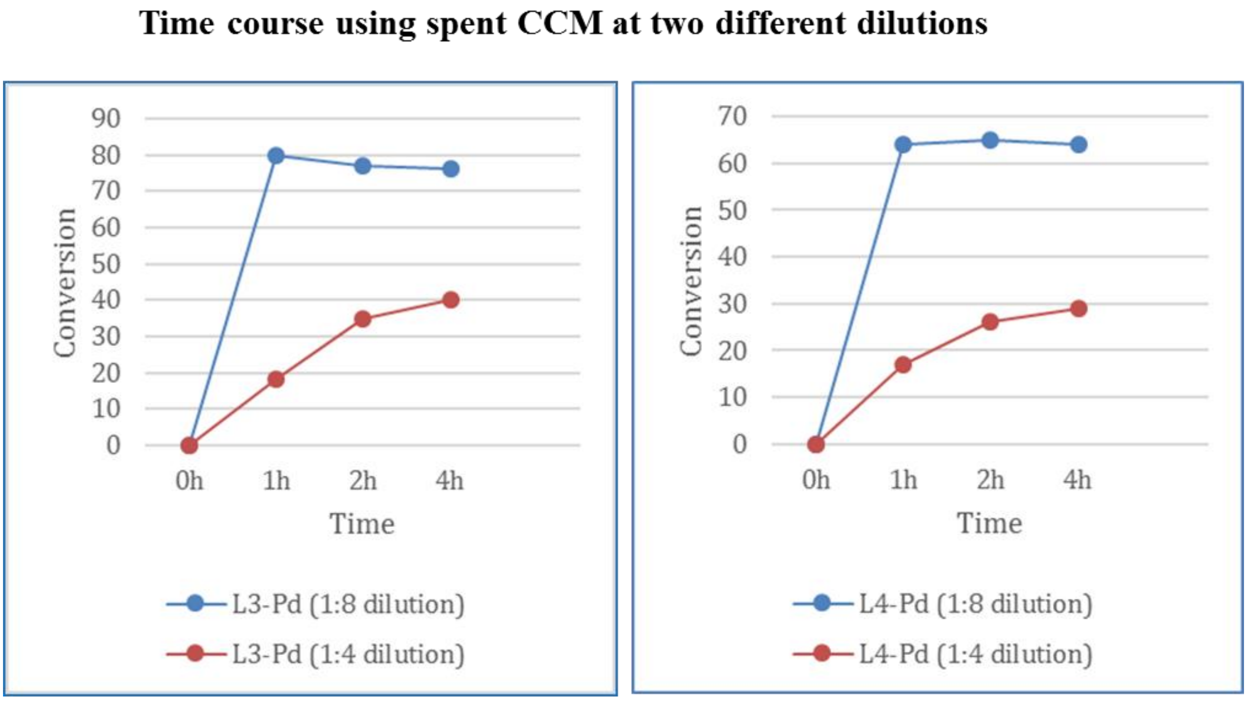

Supplementary Figure 4. Time course for the cross-coupling of 7-Br-tryptophan 2 (*in vivo* generated by *E. coli*) using spent CCM at 1:4 and 1:8 dilution with phosphate buffer. Conditions: Appropriate volume of spent culture broth in CCM containing 7-Br-tryptophan **2** generated *in vivo* (280 µM, 0.5 ml or 0.25 ml), Pd-catalyst (50 µM **L3-Pd** or **L4-Pd**), *p*-Tol-B$(OH)_2$ (1.2 mM) in phosphate buffer (total volume 2 ml, 10 mM, pH 8.5) was stirred at 37 °C. At various time points, a sample was analysed by UPLC (UPLC method 1).

L-Tryptophan (**1**)

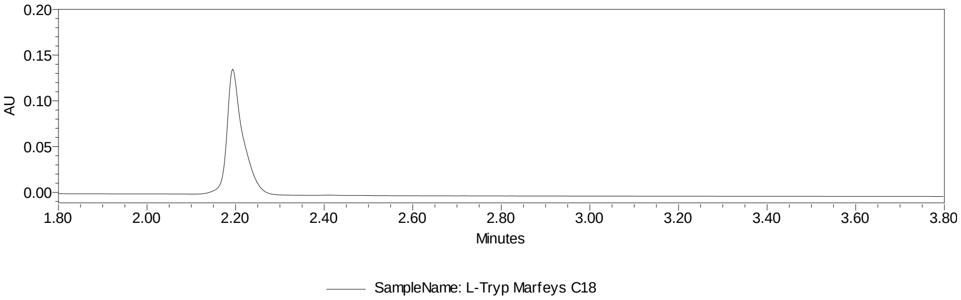

D-Tryptophan

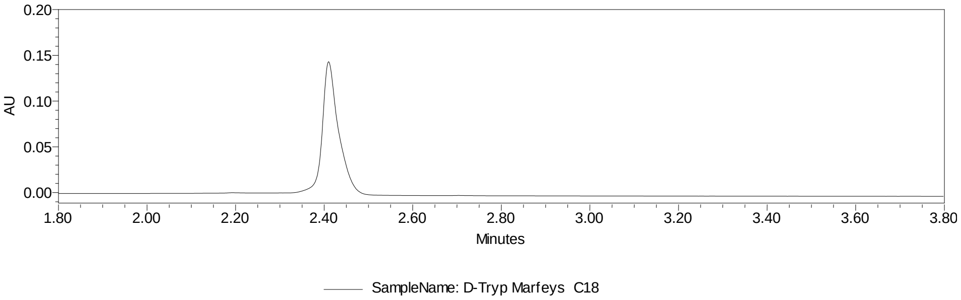

L+D-Tryptophan (2:1)

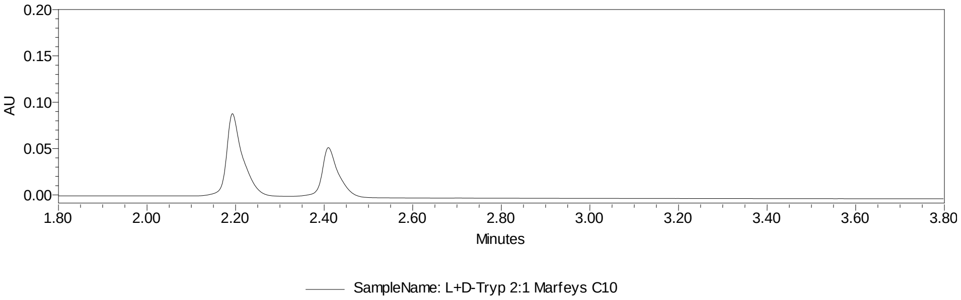

L-7-Br-tryptophan (**2**)

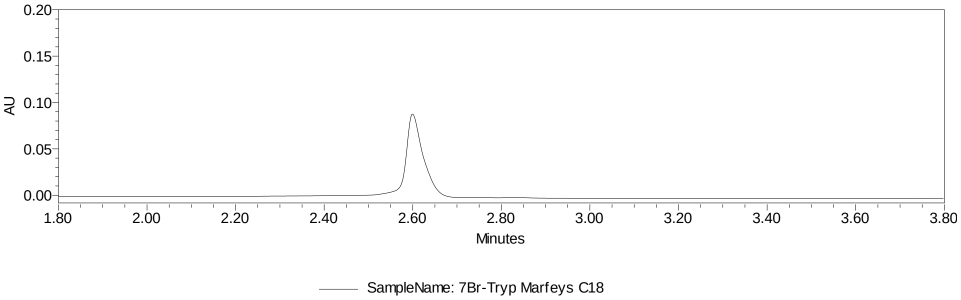

7-(*p*-tolyl)-tryptophan (**2a**)

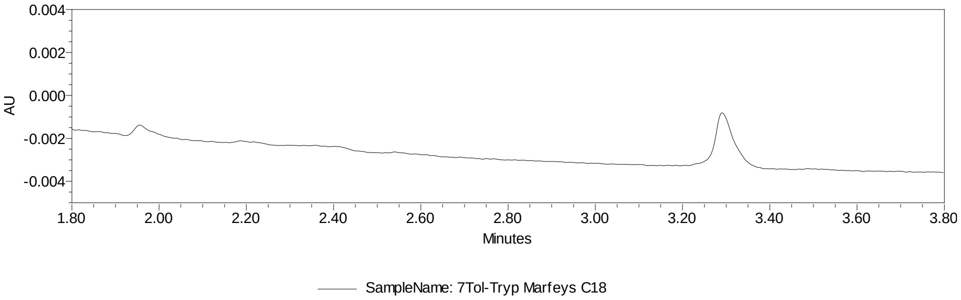

Supplementary Figure 5. UPLC chromatogram for enantiopurity determination after derivatisation with Marfey's reagent. UV detection at 340 nm.

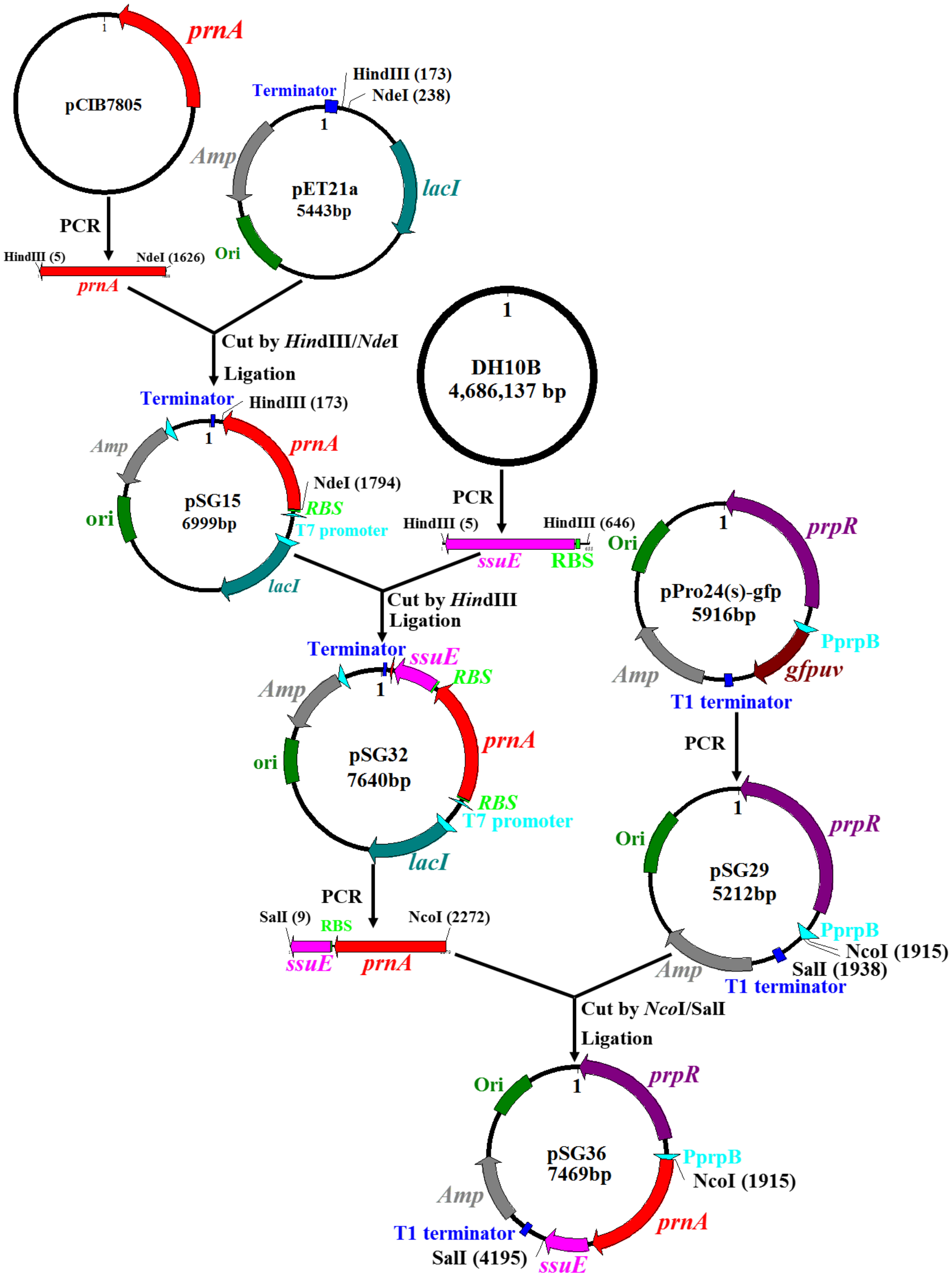

Supplementary Figure 6. Construction of recombinant plasmid of pSG36 for expression of the inserted halogenase system composed of the tryptophan 7-halogenase gene (*prnA*) and the flavin reductase gene (*ssuE*). RBS, ribosome-binding site sequence; Ori, pBR322 origin; P*prpB*, *Salmonella enterica prpBCDE* promoter; *Amp*, ampicillin resistance gene; *prpR*, a transcriptional activator gene; *lacI*, a regulatory gene for lac operon.

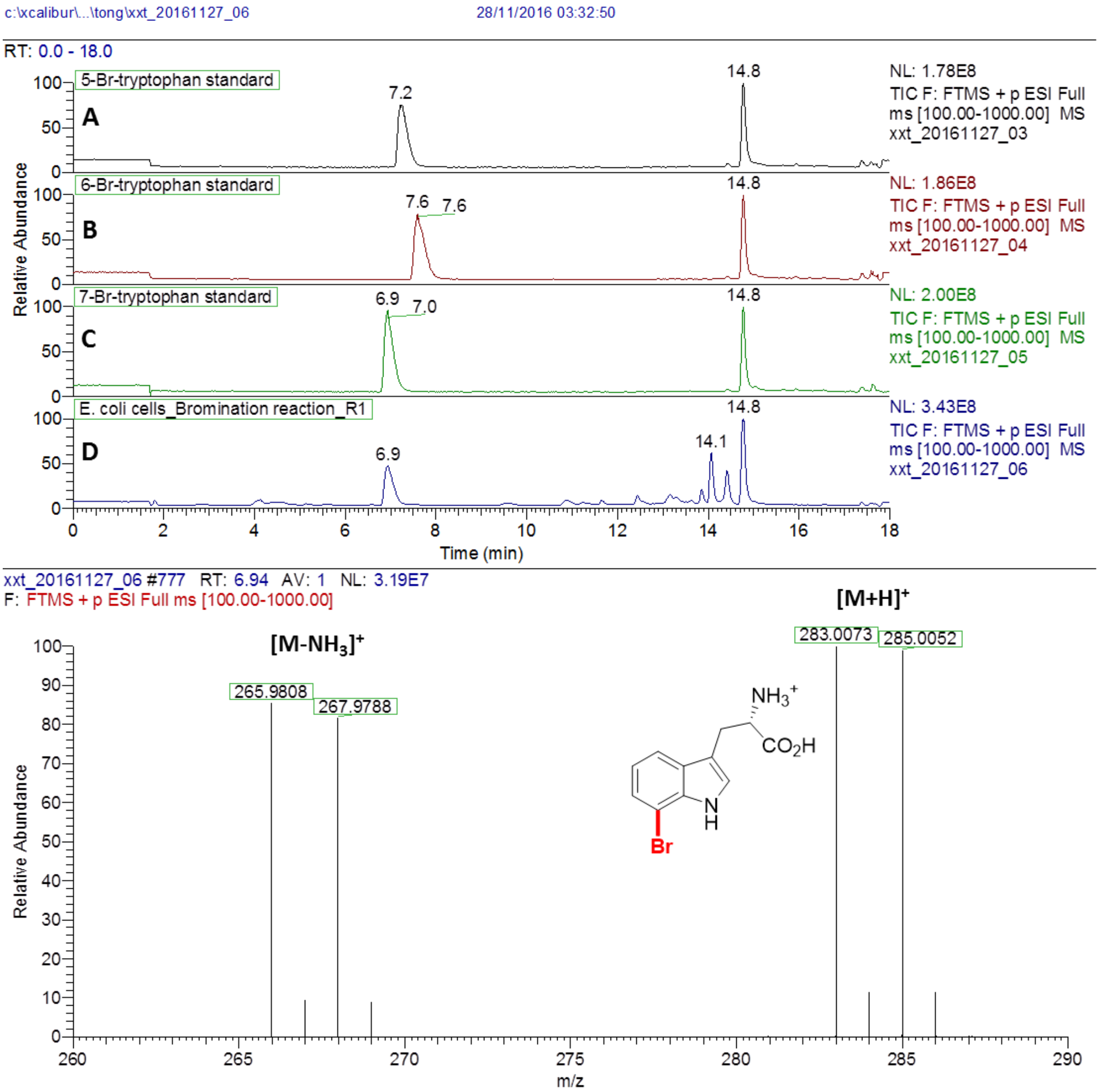

Supplementary Figure 7. LC-HRMS chromatograms and spectrum demonstrating the *in vivo* production of 7-Br-tryptophan 2 by *E. coli* RG-1500. Top: Total ion current (TIC) chromatograms of (**A**) 5-Br-tryptophan **13** standard (retention time 7.2 min), (**B**) 6-Br-tryptophan standard (retention time 7.6 min), (**C**) 7-Br-tryptophan **2** standard (retention time 6.9 min) and (**D**) a culture reaction sample with a product (**2**) peak eluting at 6.9 min. Bottom: Positive ion mode accurate mass spectrum at retention time of 6.9 min corresponding to 7-Br-tryptophan **2** peak in the chromatogram D. Masses corresponding to 7-Br-tryptophan **2** ($[M+H]^+$ *m/z* 283.0073, 285.0052 for ^{79}Br, ^{81}Br respectively) and deaminated fragment of 7-Br-tryptophan **2** ($[M+H]^+$ *m/z* 265.9808, 267.9788 for ^{79}Br, ^{81}Br respectively) were observed, data was in agreement with calculated masses and isotope pattern for bromination corresponding to expected product.

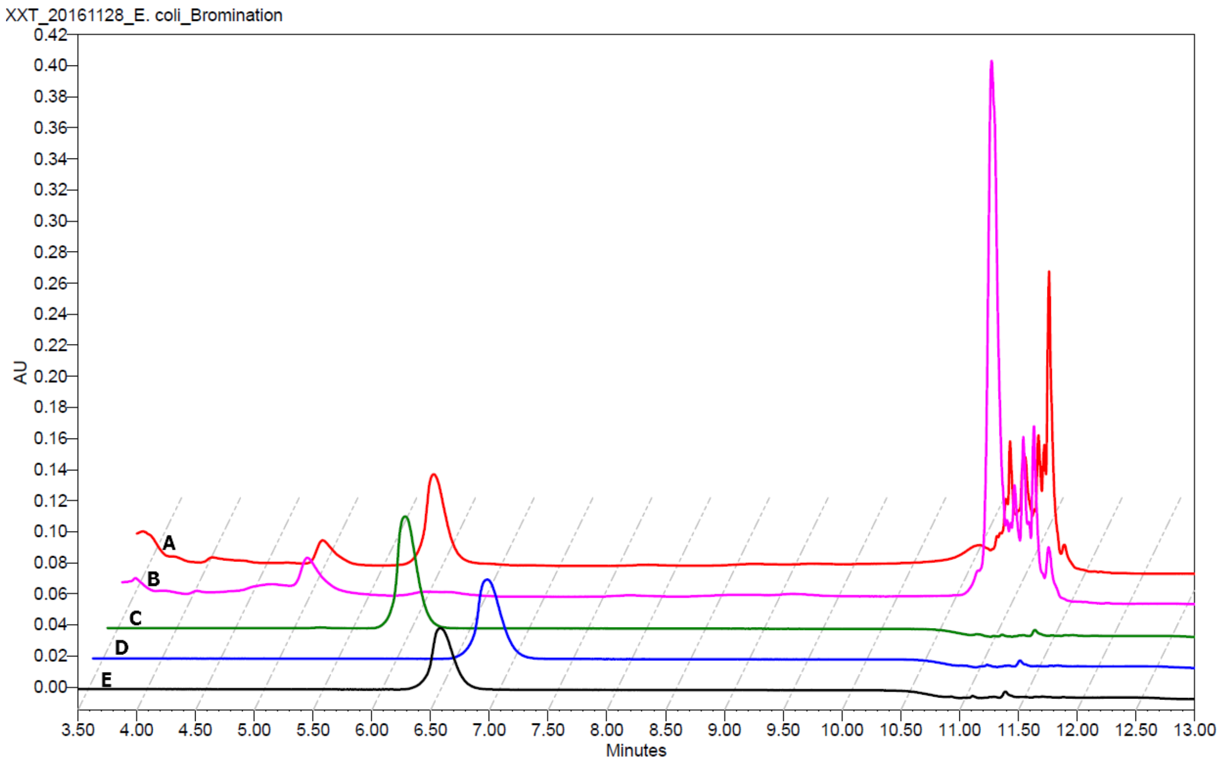

Supplementary Figure 8. UPLC chromatograms demonstrating the *in vivo* production of 7-Br-tryptophan 2 by *E. coli* RG-1500 cultures. UPLC chromatograms from (**A**) a culture sample of bromination reaction mediated by *E. coli* RG-1500 culture grown in the presence of inducer, (**B**) a control sample of bromination reaction mediated by *E. coli* culture grown without the inducer, (**C**) 7-Br-tryptophan **2** standard (retention time 5.9 min), (**D**) 6-Br-tryptophan (retention time 6.8 min) and (**E**) 5-Br-tryptophan **13** standard (retention time 6.5 min). For the reaction culture A, a peak corresponding to the 7-Br-tryptophan **2** eluting at 5.9 min was observed. In contrast, for un-induced control **B**, no peak corresponding to the 7-Br-tryptophan **2** was observed. The analysis also confirmed regioselectivity of *in vivo* bromination.

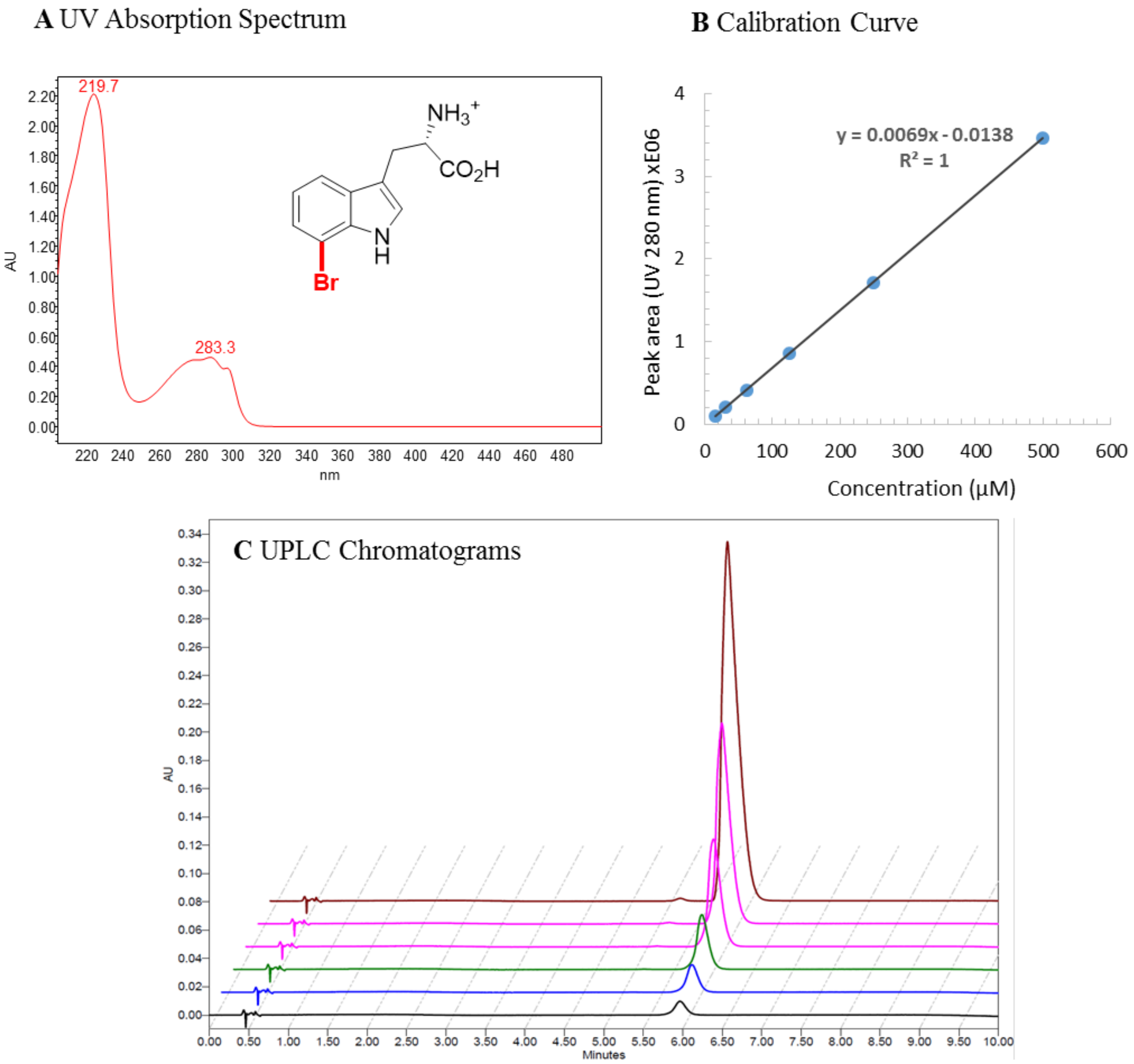

Supplementary Figure 9. UPLC analysis of pure 7-Br-tryptophan 2 standard. (**A**) UV absorption spectrum of 7-Br-tryptophan **2**; (**B**) Calibration curve of UPLC peak areas measured at 280 nm versus the concentration of the 7-Br-tryptophan **2** standard solutions ranging from 15.6 µM to 500 µM, used for determination of the concentration of the unknown; (**C**) Overlaid UPLC chromatograms for 7-Br-tryptophan **2** with increasing concentrations (15.6, 31.3, 62.5, 125, 250 and 500 µM). The peaks were monitored at UV 280 nm.

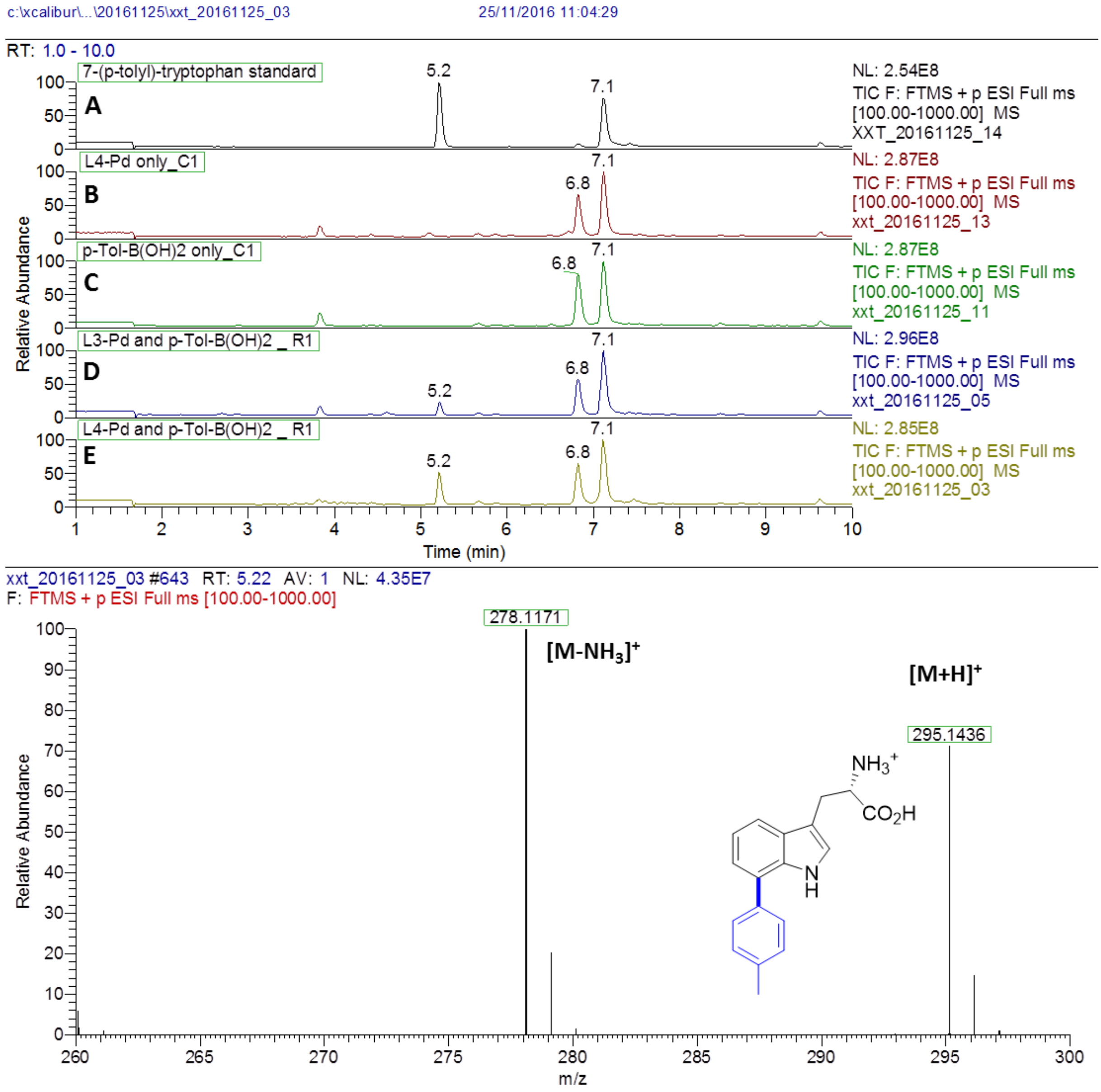

Supplementary Figure 10. LC-HRMS analysis showing the production of 7-(*p*-tolyl)-tryptophan 2a from cross-coupling in living culture of *E. coli* RG-1500. Top: Total ion current (TIC) chromatograms from (**A**) 7-(*p*-tolyl)-tryptophan **2a** standard (retention time 5.2 min), (**B**) a control sample withdrawn from cross-coupling with only 50 µM of **L4-Pd** added, (**C**) a control sample withdrawn from cross-coupling with only 1 mM of *p*-Tol-B$(OH)_2$ added, (**D**) a reaction sample withdrawn from cross-coupling with 50 µM of **L3-Pd** and 1 mM of *p*-Tol-B$(OH)_2$ added, and (**E**) a reaction sample withdrawn from cross-coupling with 50 µM of **L4-Pd** and 1 mM of *p*-Tol-B$(OH)_2$ added. Bottom: Positive ion mode accurate mass spectrum at retention time of 5.2 min corresponding to 7-(*p*-tolyl)-tryptophan **2a** peak shown in the chromatogram E. Masses corresponding to the product 7-(*p*-tolyl)-tryptophan **2a** ($[M+H]^+$ *m/z* 295.1436) and corresponding deaminated fragment of 7-(*p*-tolyl)-tryptophan **2a** ($[M+H]^+$ *m/z* 278.1171) were observed.

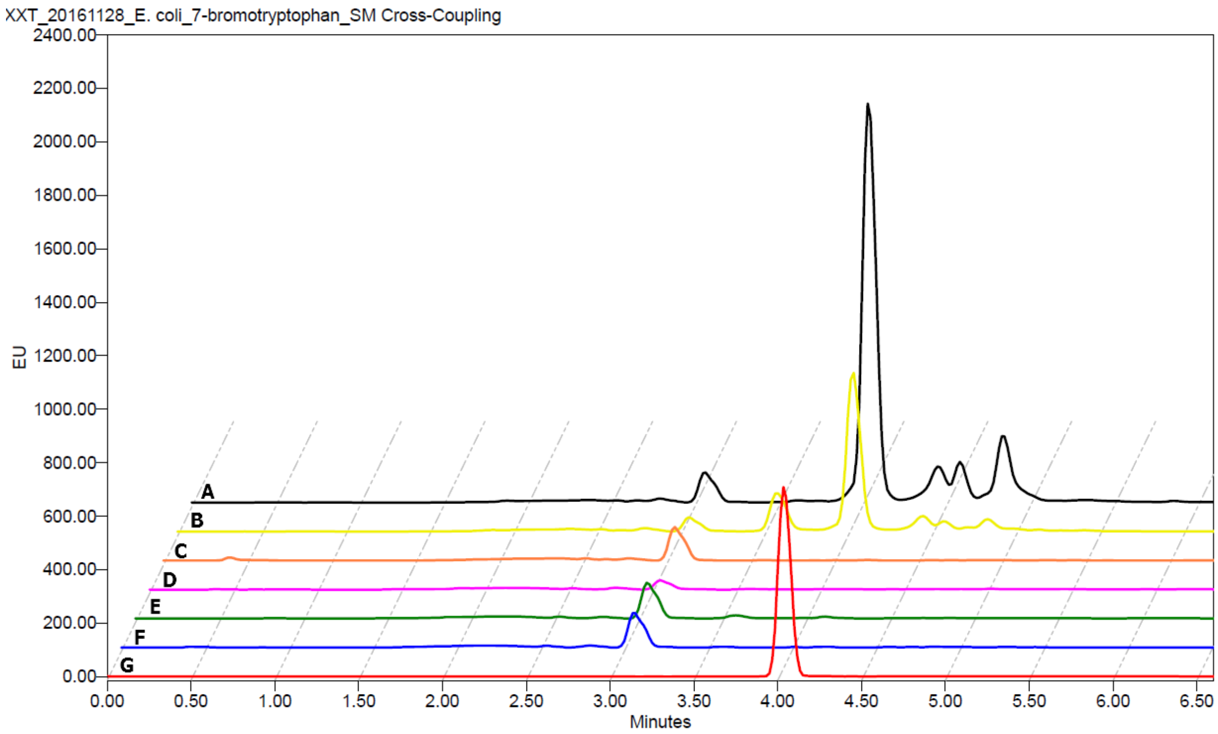

Supplementary Figure 11: UPLC analysis (UPLC method 1) of the formation of 7-(*p*-tolyl)-tryptophan 2a from cross-coupling in living culture of *E. coli* RG-1500. UPLC chromatograms from (**A**) a reaction sample withdrawn from cross-coupling with 50 µM of **L4-Pd** and 1 mM of *p*-Tol-B$(OH)_2$ added, (**B**) a reaction sample withdrawn from cross-coupling with 50 µM of **L3-Pd** and 1 mM of *p*-Tol-B$(OH)_2$ added, (**C**) a control sample withdrawn from cross-coupling with only 50 µM of **L4-Pd** added, (**D**) a control sample withdrawn from cross-coupling with only 50 µM of **L3-Pd** added, (**E**) a control sample withdrawn from cross-coupling with only 1 mM of *p*-Tol-B$(OH)_2$ added, (**F**) a control sample withdrawn from cross-coupling with no addition of Pd-catalyst and *p*-Tol-B$(OH)_2$, and (**G**) 7-(*p*-tolyl)-tryptophan **2a** standard (retention time 4.0 min). For the reaction sample, a peak corresponding to the 7-(*p*-tolyl)-tryptophan product **2a** eluting at 4.0 min was observed. In contrast, no peak corresponding to the 7-(*p*-tolyl)-tryptophan **2a** was present in the control samples C-F.

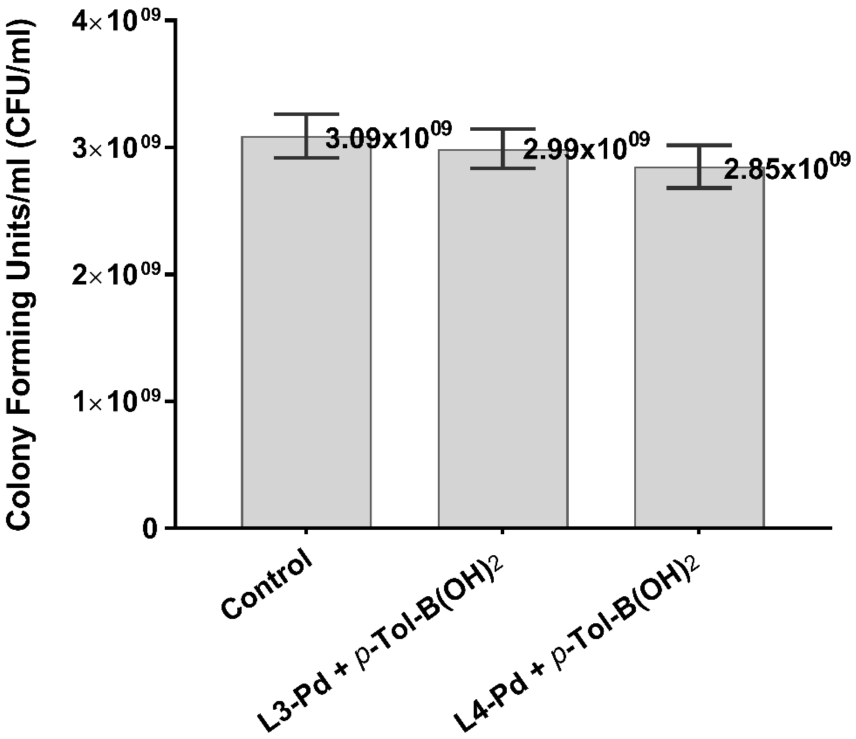

Supplementary Figure 12. Determination of the toxicity of cross-coupling conditions on *E. coli* RG-1500 by CFU method. 50 µM of Pd-catalyst (**L3-Pd** or **L4-Pd**) and 1 mM of *p*-Tol-$B(OH)_2$ were present in the reaction sample. The control was *E. coli* RG-1500 culture without treatment with both the Pd-catalyst and *p*-Tol-$B(OH)_2$. The number of *E. coli* RG-1500 cells per ml of the original cell solution was calculated using the following solution: number of CFU/ml of original cell solution = [number of CFU/volume plated (ml)] × total dilution used. The data was presented as mean ± standard deviation.

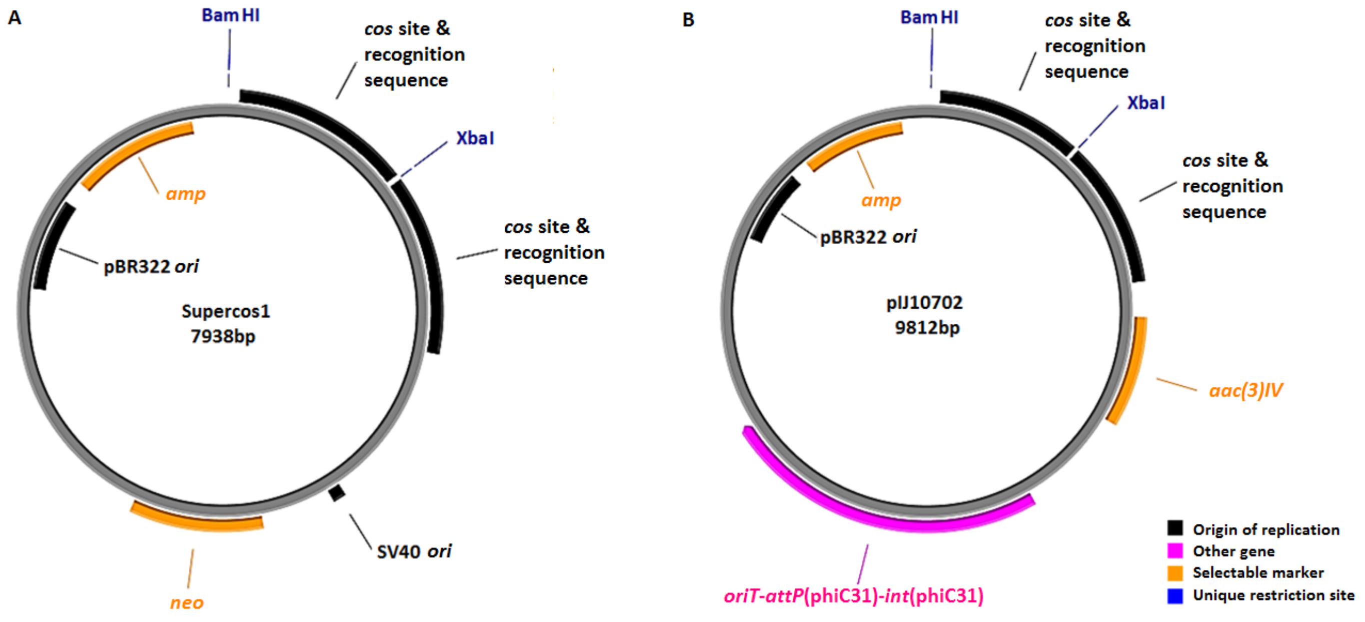

Supplementary Figure 13. An integration cassette for heterologous expression of the pacidamycin genes. (**A**) Supercos I vector (Stratagene) and (**B**) the pIJ10702-derivative harbouring a cassette for conjugal transfer and site specific integration in the *Streptomyces* genome (provided by M. Bibb, John Innes Centre). The integration cassette contains the apramycin-resistance gene for selection (*aac(3)IV*), an origin of transfer for conjugation and a site-specific attachment site and integrase (*attP*(ΦC31)-*int*(ΦC31)) for genomic insertion. The *cos* sites (*cos*), resistance genes (*amp*, *neo*, *aac(3)IV*), origin of replication (*ori*) and promoter (pSV40) are also shown. Original images were created using the PlasMapper tool and edited.[1]

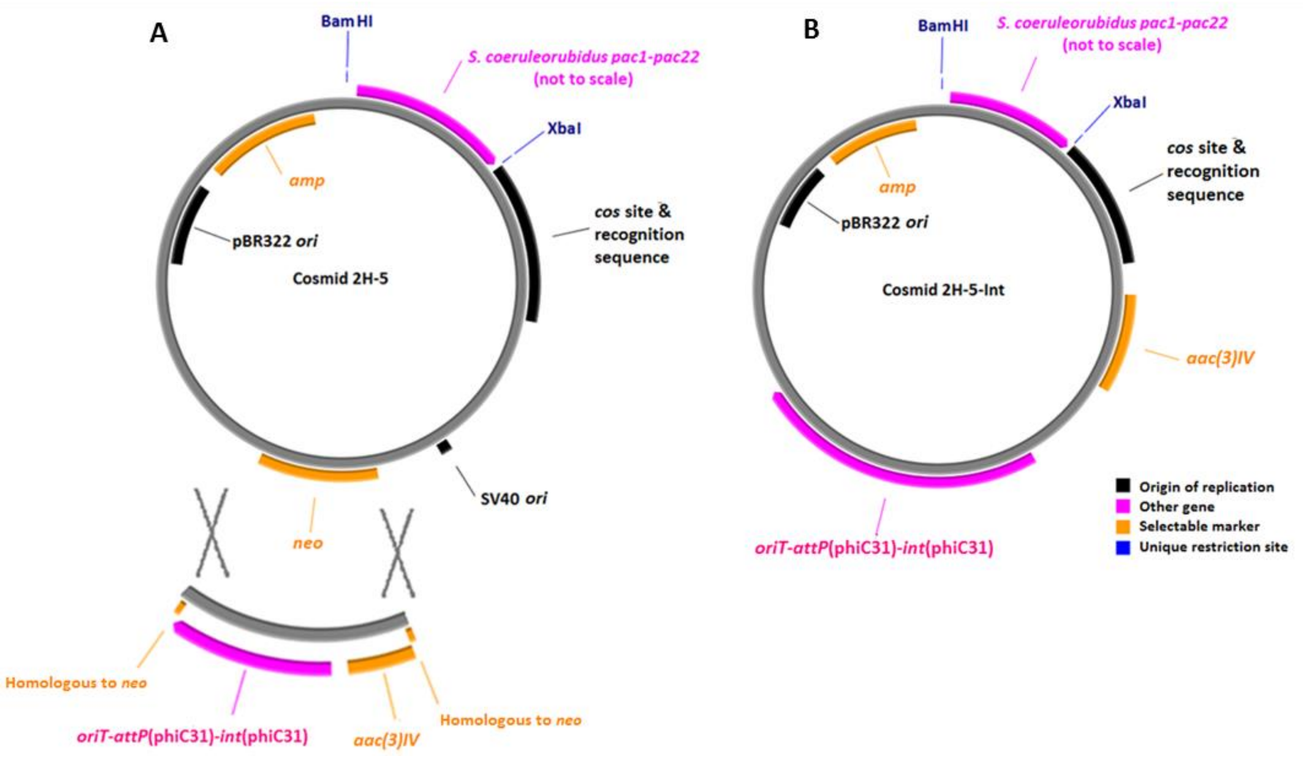

Supplementary Figure 14. Generation of *S. coeruleorubidus* cosmid for integration into heterologous streptomyces host strains using the PCR-targeting technology.[2] (**A**) The pIJ10702-derived integration cassette is amplified with PCR primers with 5'-regions homologous to regions flanking the *neo* gene of Supercos 1 (shown in orange). λ red-mediated recombination between the PCR-targeted integration cassette and the *neo* gene of the cosmid yields 2H-5-Int. (**B**) Cosmid 2H-5-Int containing the *aac*(3)*IV*-*oriT*-*att*P(Φ31)-*Int*(Φ31) integration cassette for introduction and site-specific integration into heterologous *S. coelicolor* host.

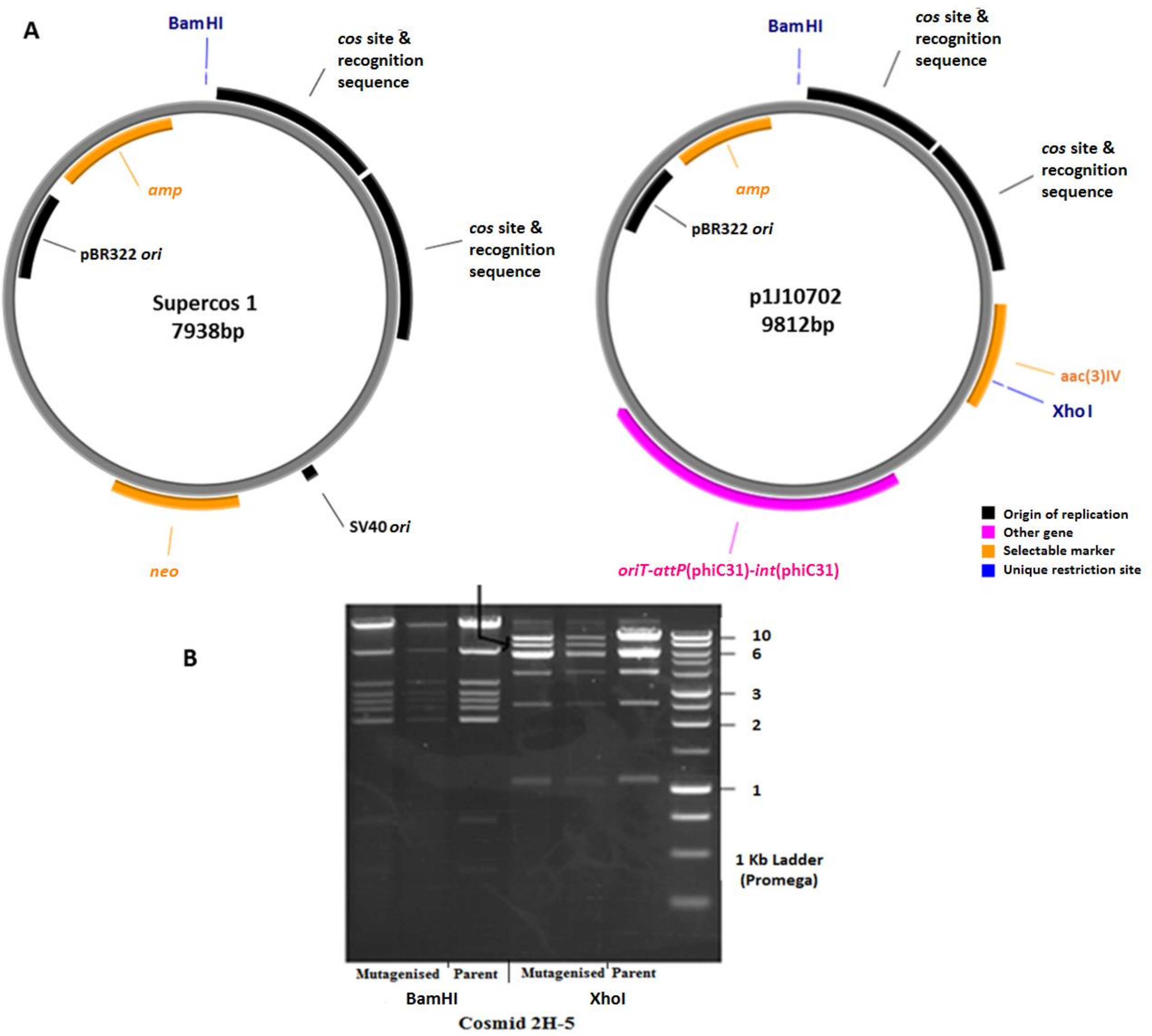

Supplementary Figure 15. Mutagenesis of *S. coeruleorubidus* cosmid 2H-5 by the introduction of an integration cassette enabling heterologous expression by streptomycetes. (**A**) Parent cosmid backbone and integration cosmid backbone, the *S. coeruleorubidus* cosmid 2H-5 also contains the pacidamycin genes within the BamHI and XhoI restriction sites. (**B**) Restriction digest of parent and integration cosmids. The arrow indicates the new DNA band observed from digestion of the integration cosmid.

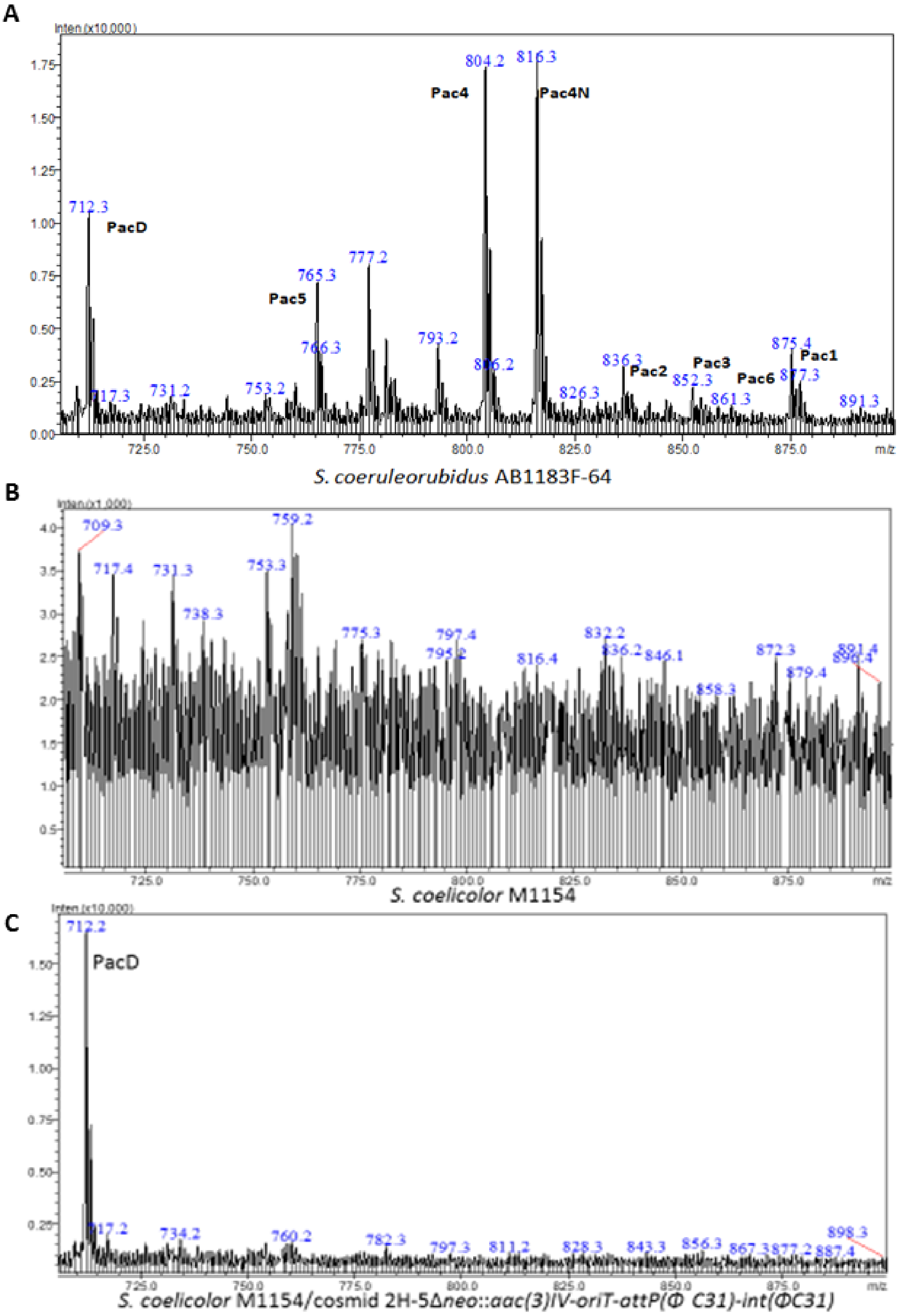

Supplementary Figure 16. Initial heterologous expression of pacidamycins in *S. coelicolor* strains. Mass spectra (*m/z* 700-900, at the University of East Anglia, prior to Goss group move to St Andrews) illustrating pacidamycins produced by (**A**) *S. coeruleorubidus*, (**B**) *S. coelicolor* M1154 wild-type strains (indicating absence of any pacidamycins) and (**C**) strains harbouring the pacidamycin genes (showing production of pacidamycin D). The presence of pacidamycins in culture extracts was deduced by the detection of characteristic masses (*m/z* 712, 875, 836, 852, 804, 765, 861, 816) corresponding to reported pacidamycins at the established retention time (3.8-5 minutes). The proposed bicyclic derivatives of pacidamycins 5 and 5T (*m/z* 777 and 793) were also observed.

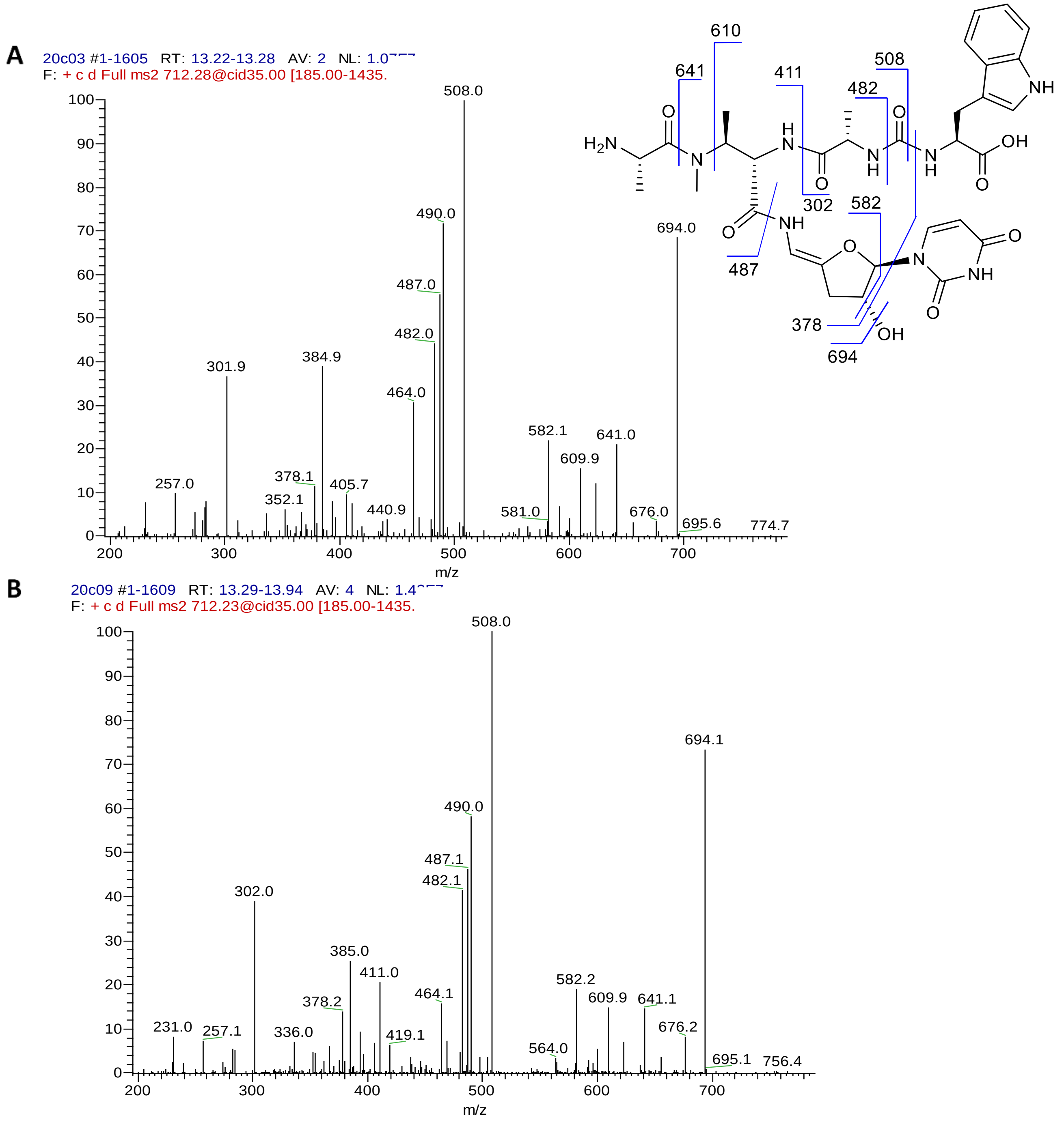

Supplementary Figure 17. Confirmation of pacidamycin D production by an *S. coelicolor* heterologous expression strain harbouring the pacidamycin biosynthetic genes on cosmid 2H-5-Int. (**A**) MS2 fragmentation analysis of pacidamycin D produced by *S. coeruleorubidus* and (**B**) MS2 fragmentation of pacidamycin D produced by *S. coelicolor* M1154 harbouring the pacidamycin genes on cosmid 2H-5-Int. Performed at the University of East Anglia. The inset indicates a possible fragmentation of pacidamycin D.

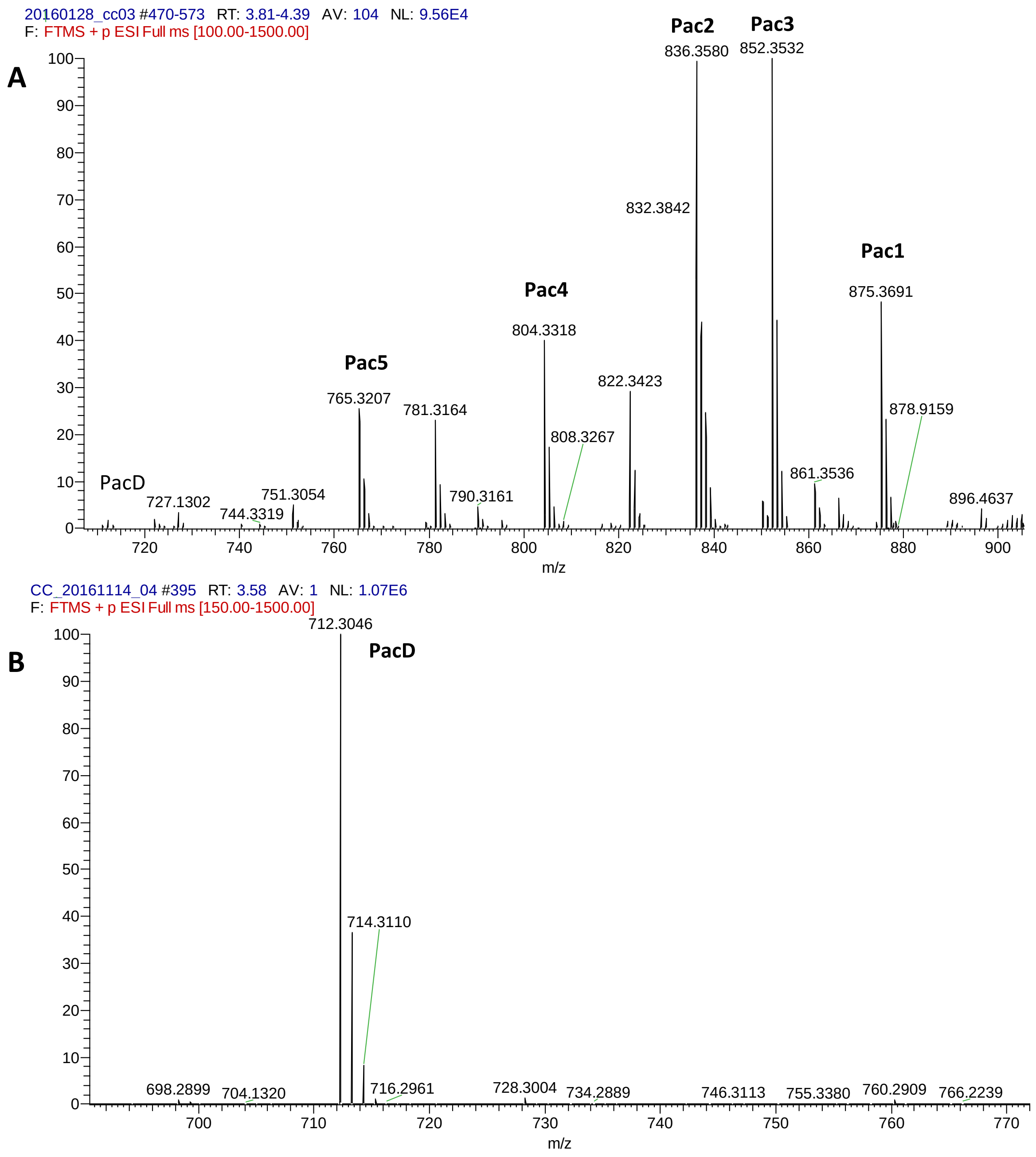

Supplementary Figure 18. Subsequent production of pacidamycins by *S. coelicolor* RG-4242 harbouring the pacidamycin biosynthetic genes. Mass spectra illustrating pacidamycin production by (**A**) Natural producer: *S. coeruleorubidus*, (**B**) *S. coelicolor* M1154 harbouring the minimal pacidamycin gene cluster and renamed *S. coelicolor* RG-4242, producing pacidamycin D. This is confirmation of the initial screening experiments performed at the University of East Anglia with heterologous expression strains.

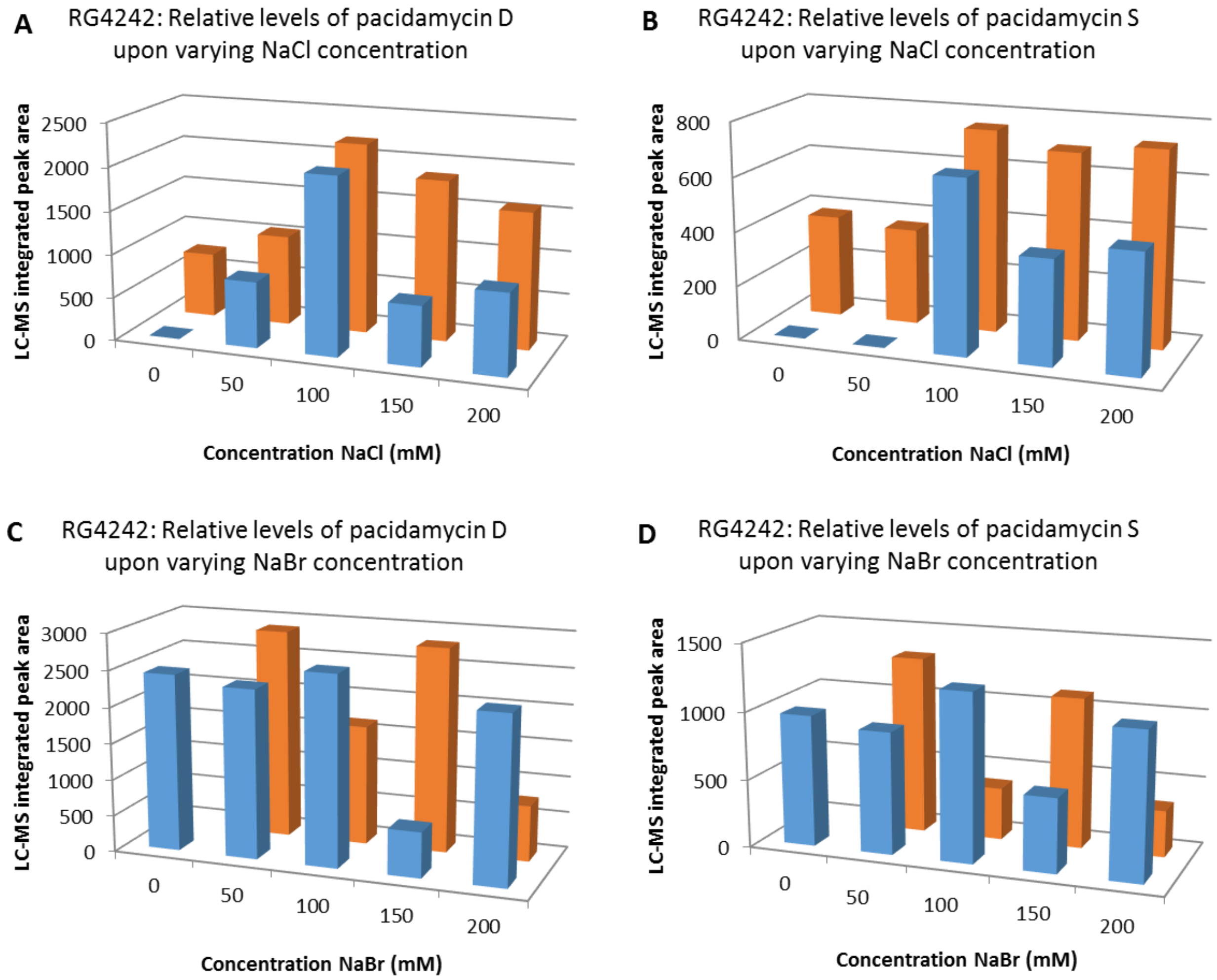

Supplementary Figure 19. Production of pacidamycin D (A, C) and pacidamycin S (B, D) from RG-4242 cultures supplemented with varying concentrations of NaCl or NaBr. (**A and B**) Pacidamycin production was observed with concentrations of up to 200 mM of NaCl. The highest levels of pacidamycin production were observed at a concentration 100 mM of NaCl. (**C and D**) Pacidamycin production from cultures supplemented with NaBr (50-100 mM) was sligtly higher than with cultures supplemented with NaCl.

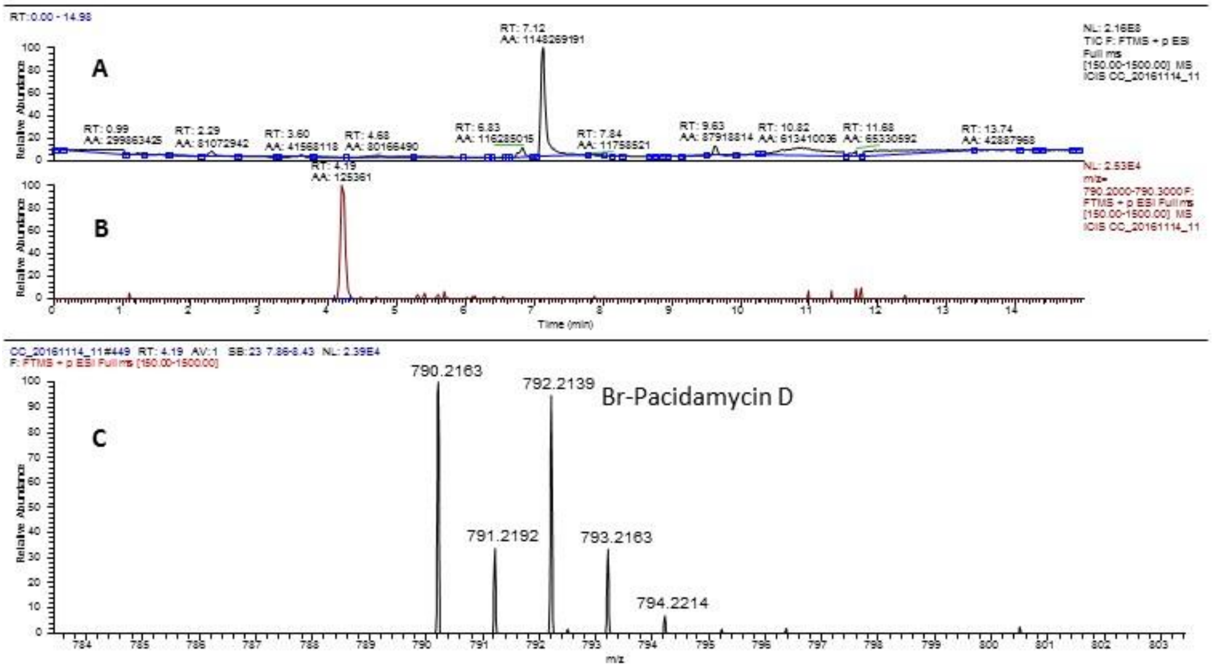

Supplementary Figure 20. Mass spectrometry analysis of RG-1104 culture extracts. (**A**) TIC of *S. coelicolor* RG-1104 culture extract, post XAD-16 fractionation, (**B**) EIC for Br-pacidamycin D **3** (*m/z*= 790, 792 for ^{79}Br, ^{81}Br respectively), (**C**) Extracted mass spectrum at showing the presence of Br-pacidamycin D **3**.

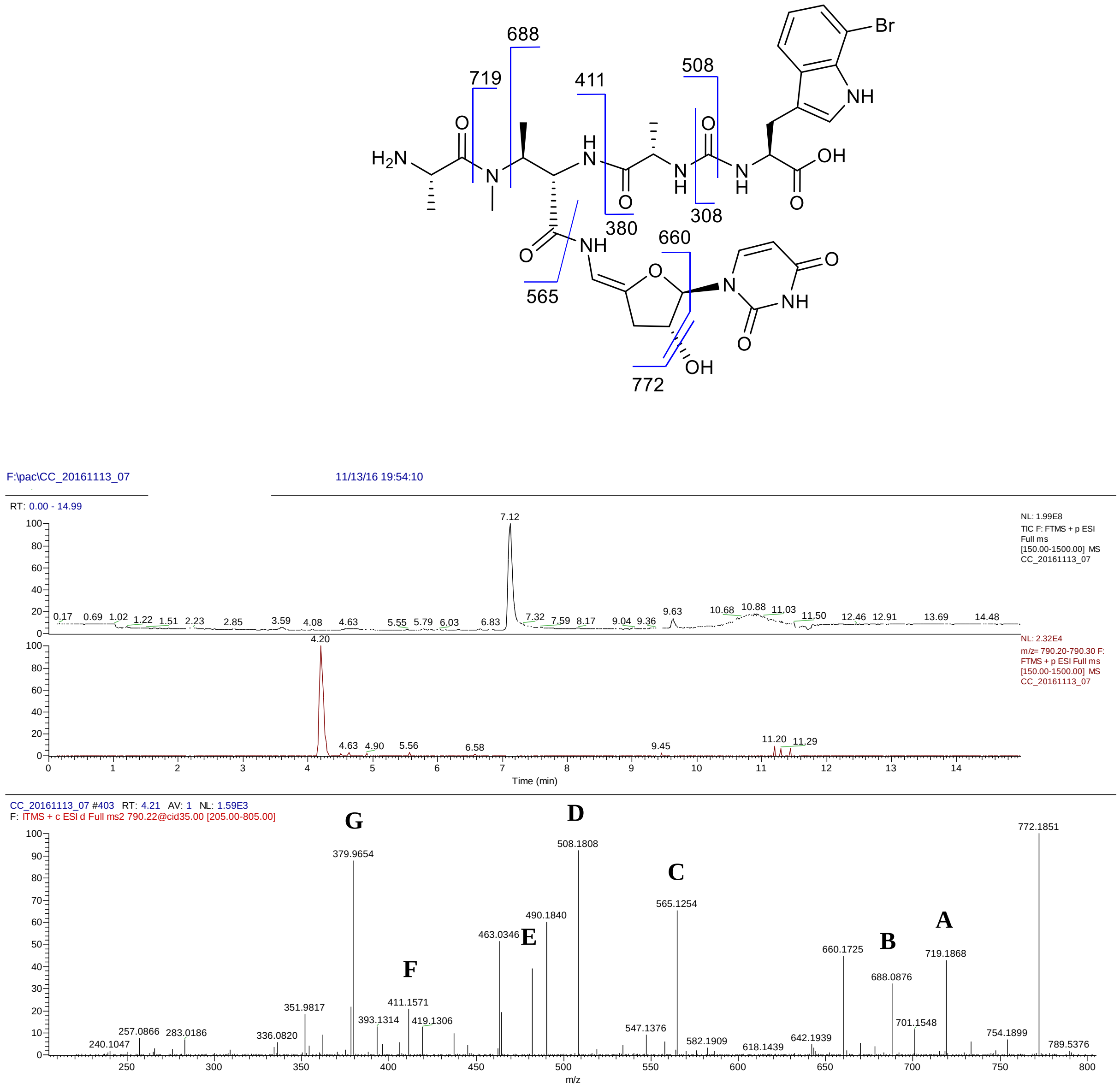

Supplementary Figure 21. MS2 fragmentation of Br-pacidamycin D 3 for *m/z* 790 (for ^{79}Br). Details of the predicted fragmentations and the structures corresponding to the annotated masses is presented in Supplementary Fig. 27.

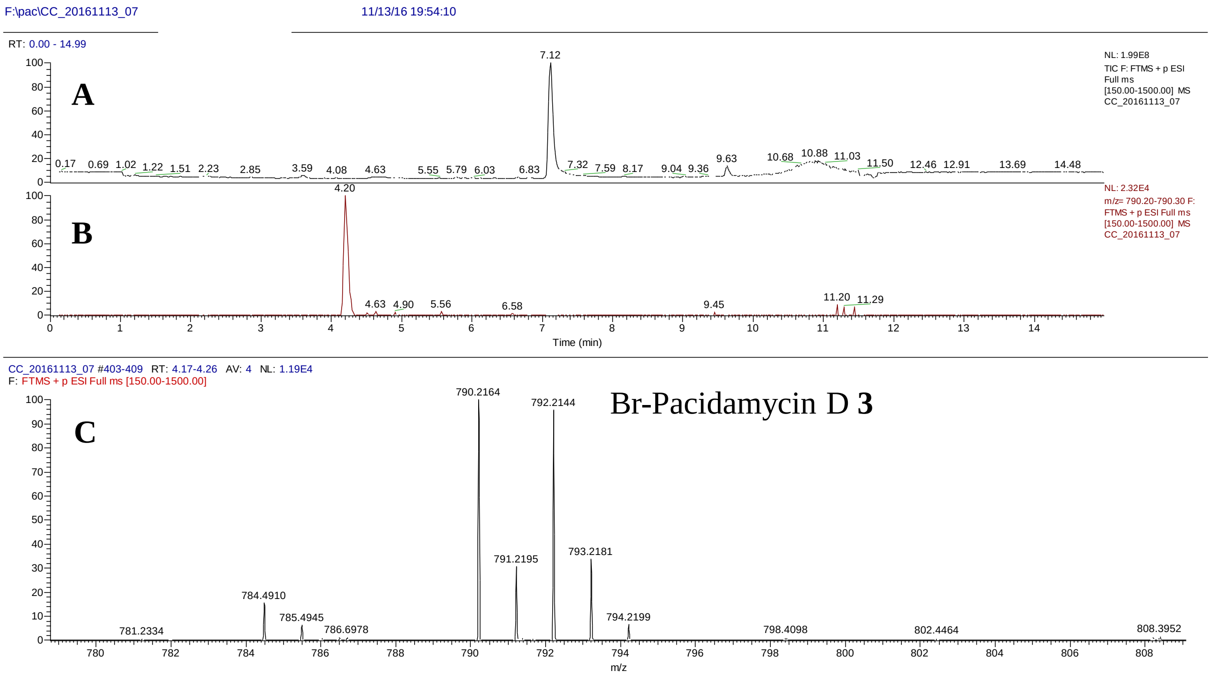

Supplementary Figure 22. Mass spectrometry analysis of RG-1104 culture extracts grown in CCM. (**A**) TIC of *S. coelicolor* RG-1104 culture, (**B**) EIC for Br-pacidamycin D **3** (*m/z*= 790, 792 for ^{79}Br, ^{81}Br respectively), (**C**) Extracted mass spectrum at showing the presence of Br-pacidamycin D **3**.

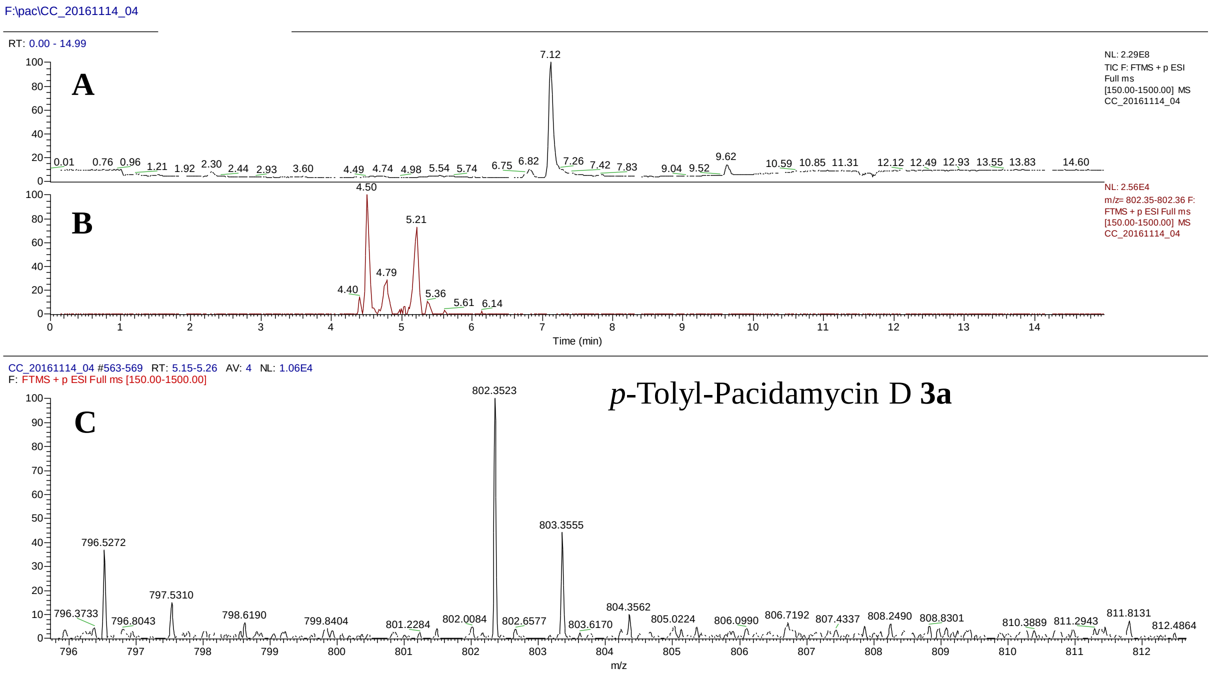

Supplementary Figure 23. LCMS chromatogram showing presence of *p*-tolyl pacidamycin D 3a (retention 5.21 min, *m/z* 802.3523) produced within CCM. (**A**) TIC of *S. coelicolor* RG-1104 culture after cross-coupling, (**B**) EIC for *p*-tolyl pacidamycin D **3a** (*m/z*= 802), (**C**) Extracted mass spectrum at showing the presence of *p*-tolyl pacidamycin D **3a**.

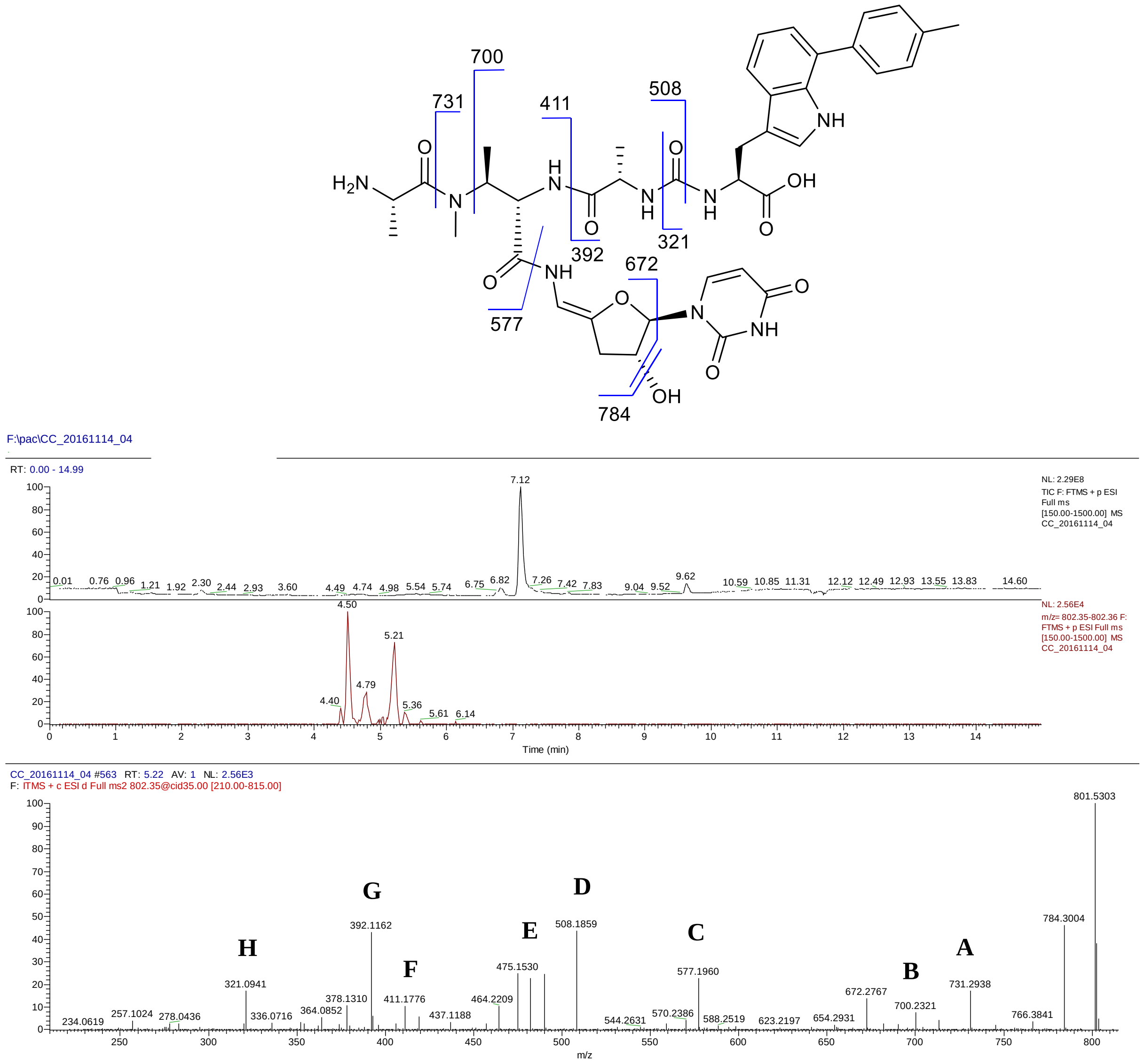

Supplementary Figure 24. MS2 fragmentation of *p*-tolyl-pacidamycin D 3a for *m/z* 802.35. Details of the predicted fragmentations and the structures corresponding to the annotated masses is given in Supplementary Fig. 28.

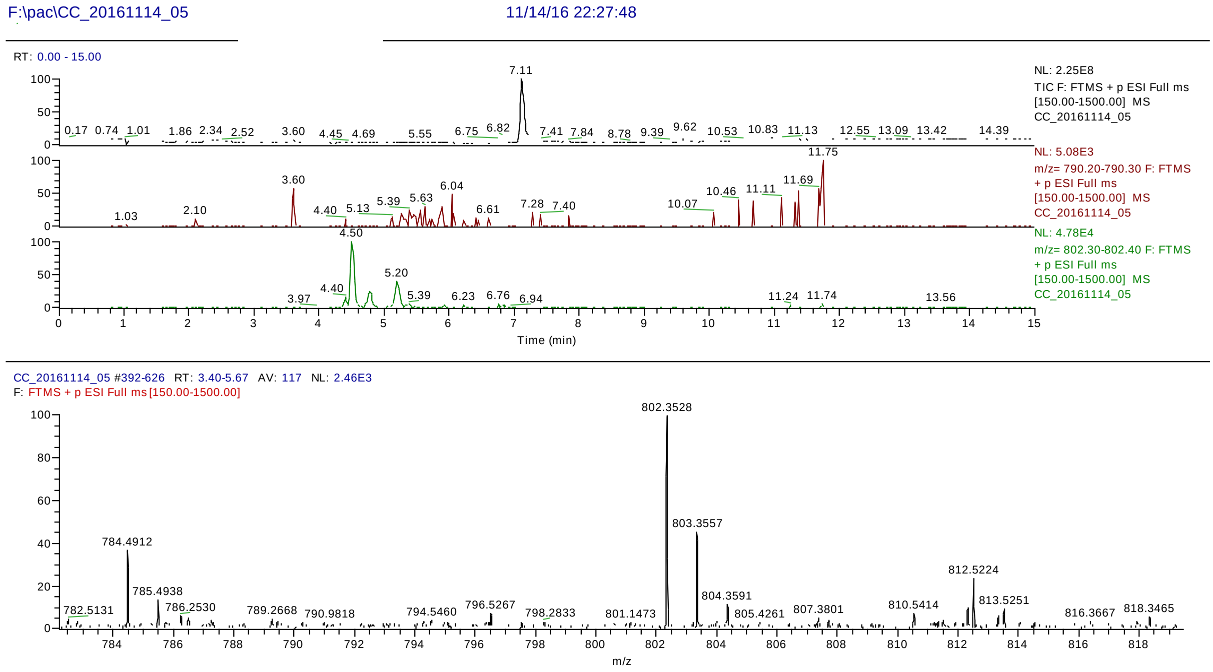

Supplementary Figure 25. Complete consumption of Br-pacidamycin D 3 was observed *via* LCMS. A mass range of 784-818 *m/z* was used. Both the LC and MS chromatograms indicated that no Br-pacidamycin D **3** remained. (The peak with retention time at 4.2 mins, and with masses of 790 and 792, for ^{79}Br, ^{81}Br respectively, could no longer be observed), this is indicative of the complete consumption of Br-pacidamycin D **3**.

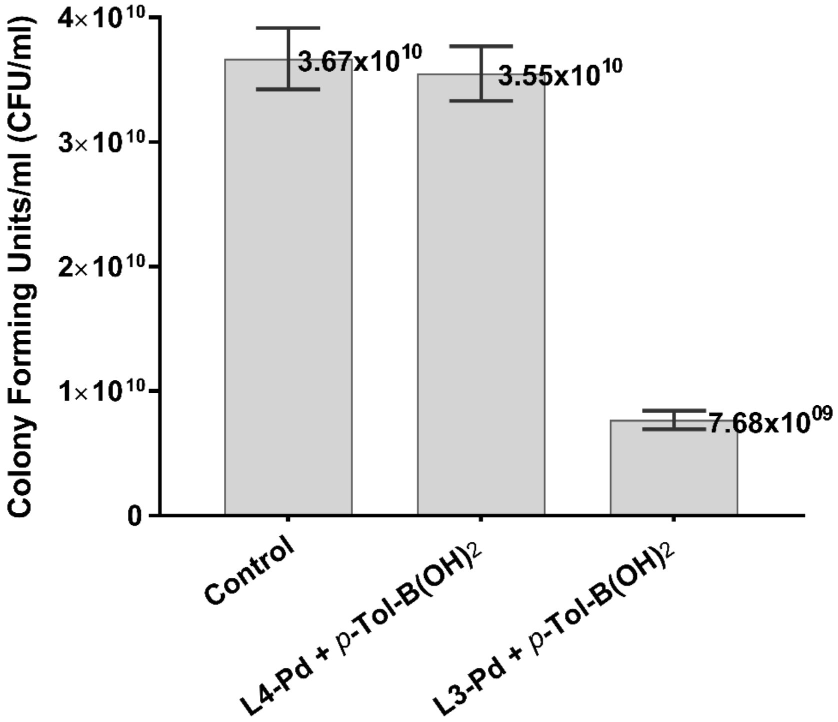

Supplementary Figure 26. Determination of cell viability of *S. coelicolor* cultures by CFU counting method.

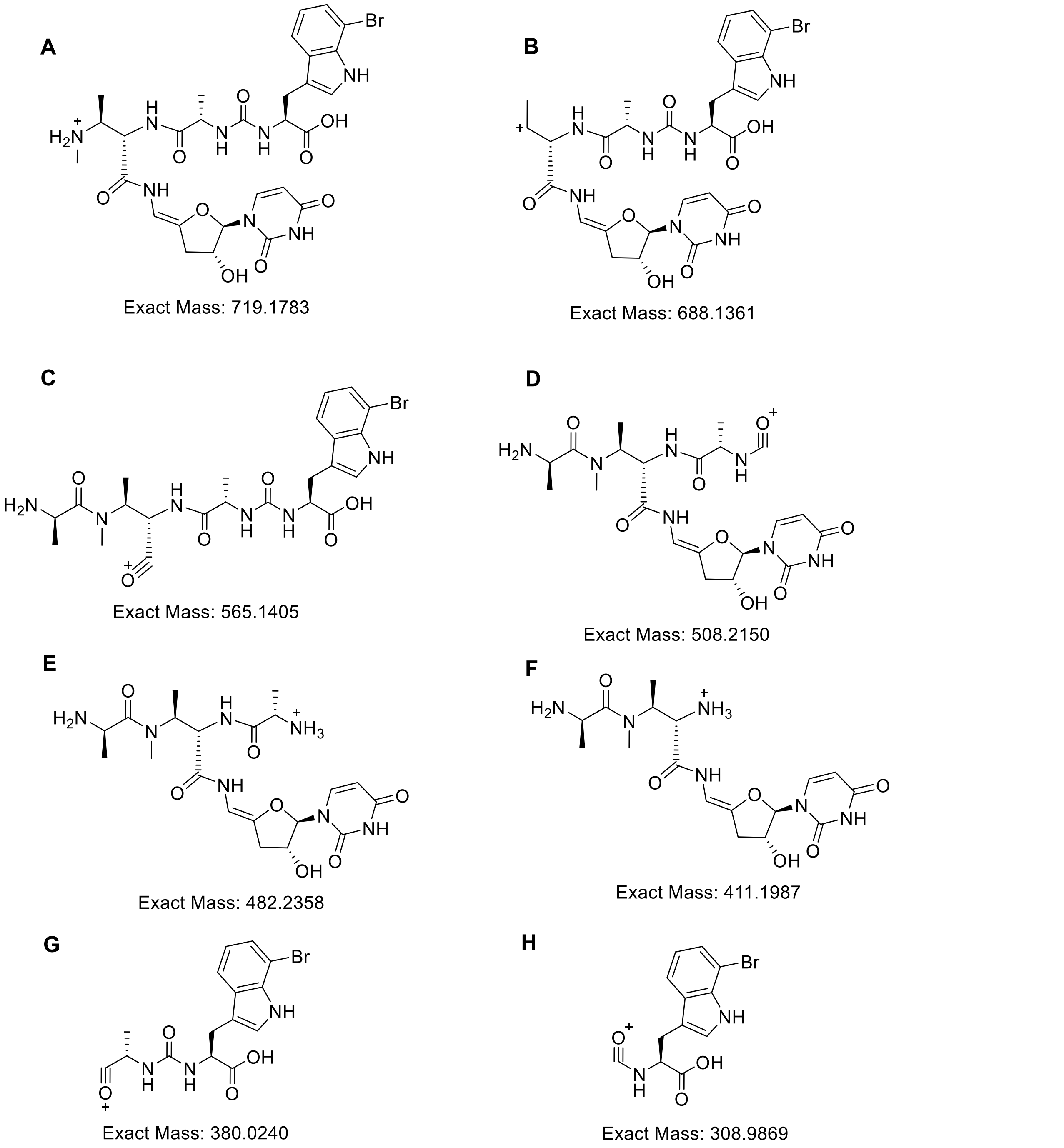

Supplementary Figure 27. Predicted fragmentation of Br-pacidamycin D 3 (*m/z* 790 for ^{79}Br, $[M+H]^{+}$) used for MS2 analysis.

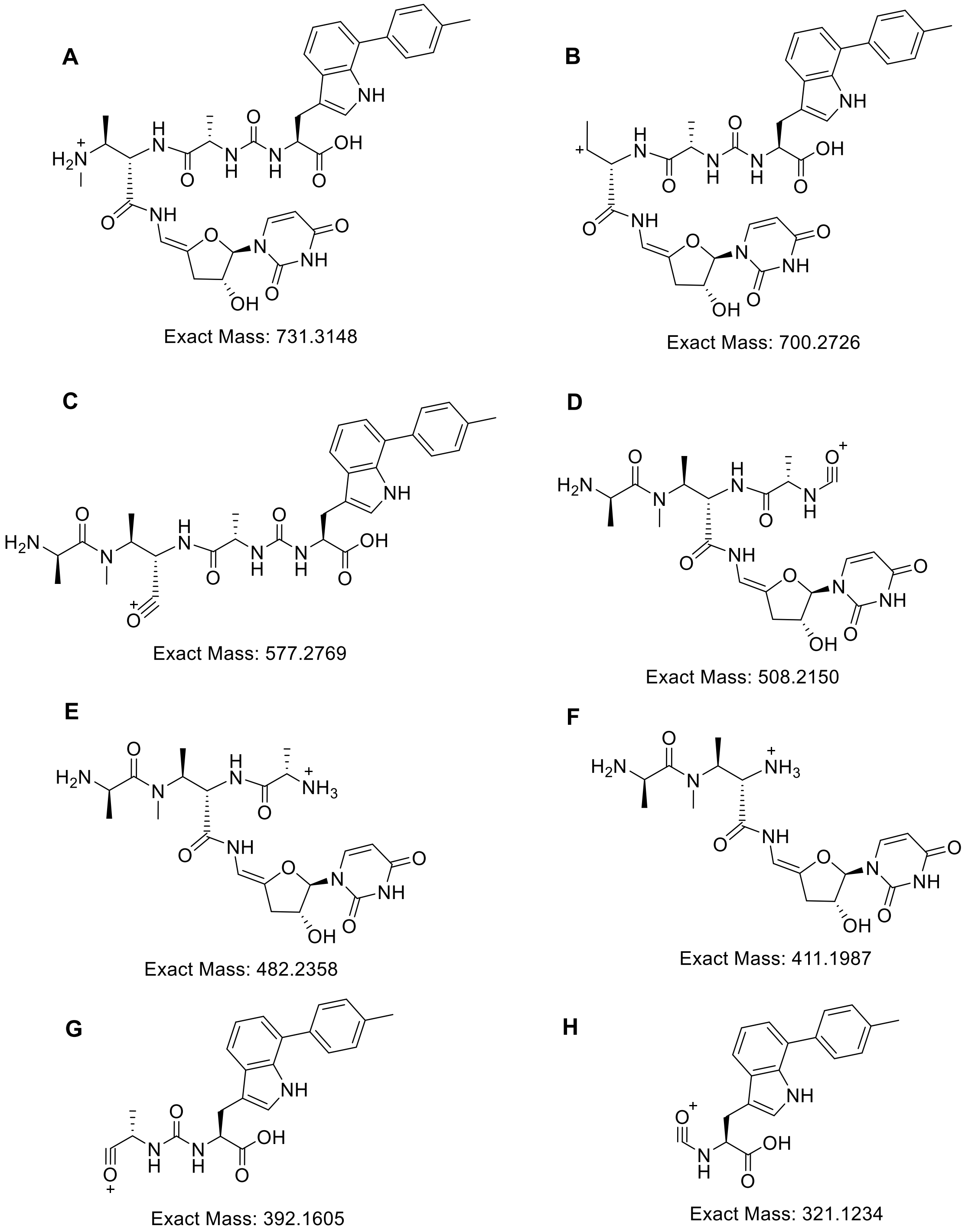

Supplementary Figure 28. Predicted fragmentation of *p*-tolyl-pacidamycin D 3a (*m/z* 802 $[M+H]^+$) used for MS2 analysis.

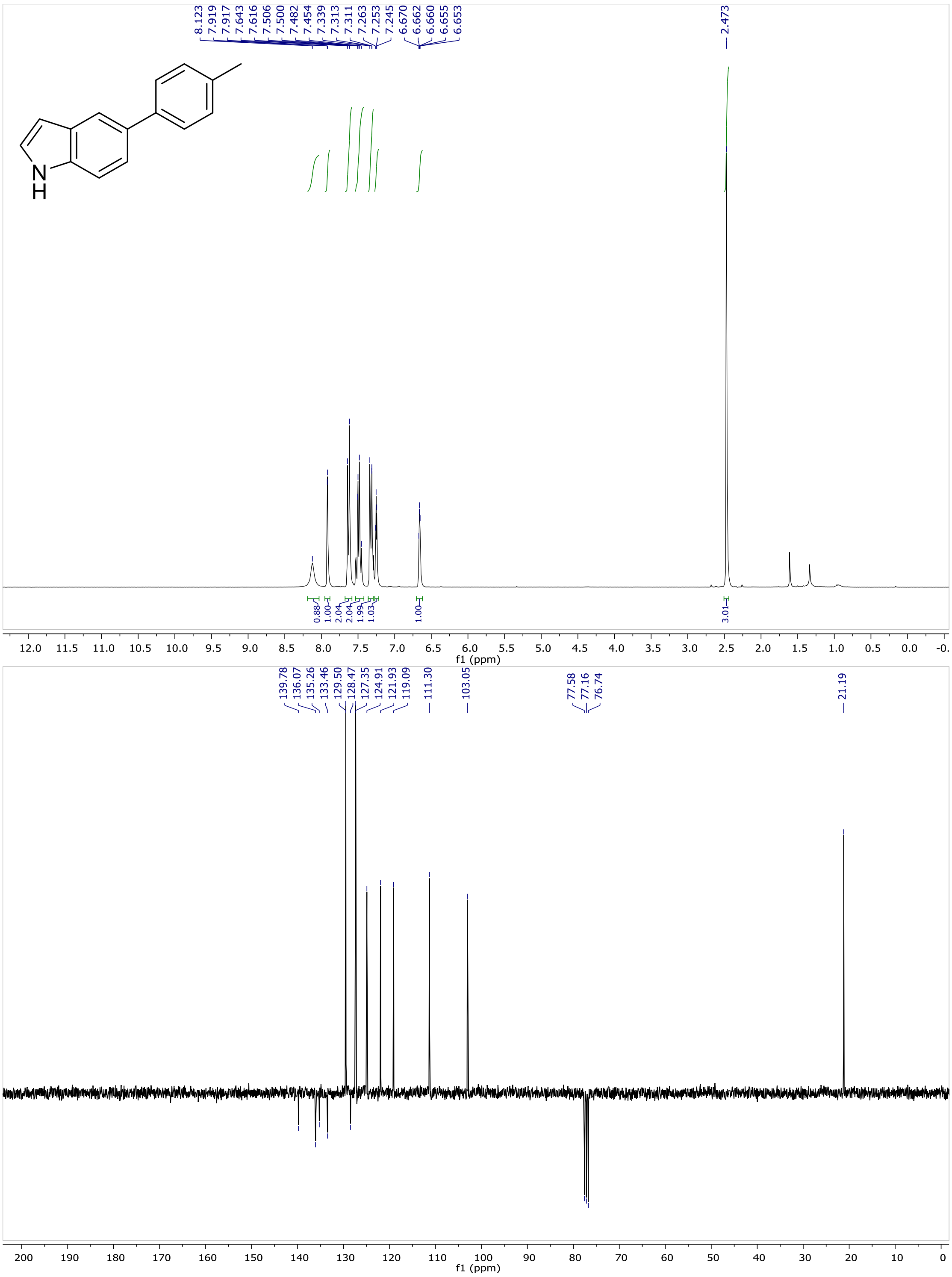

Supplementary Figure 29. ^{1}H and ^{13}C NMR of 5-(*p*-tolyl)-1H-indole 5a.

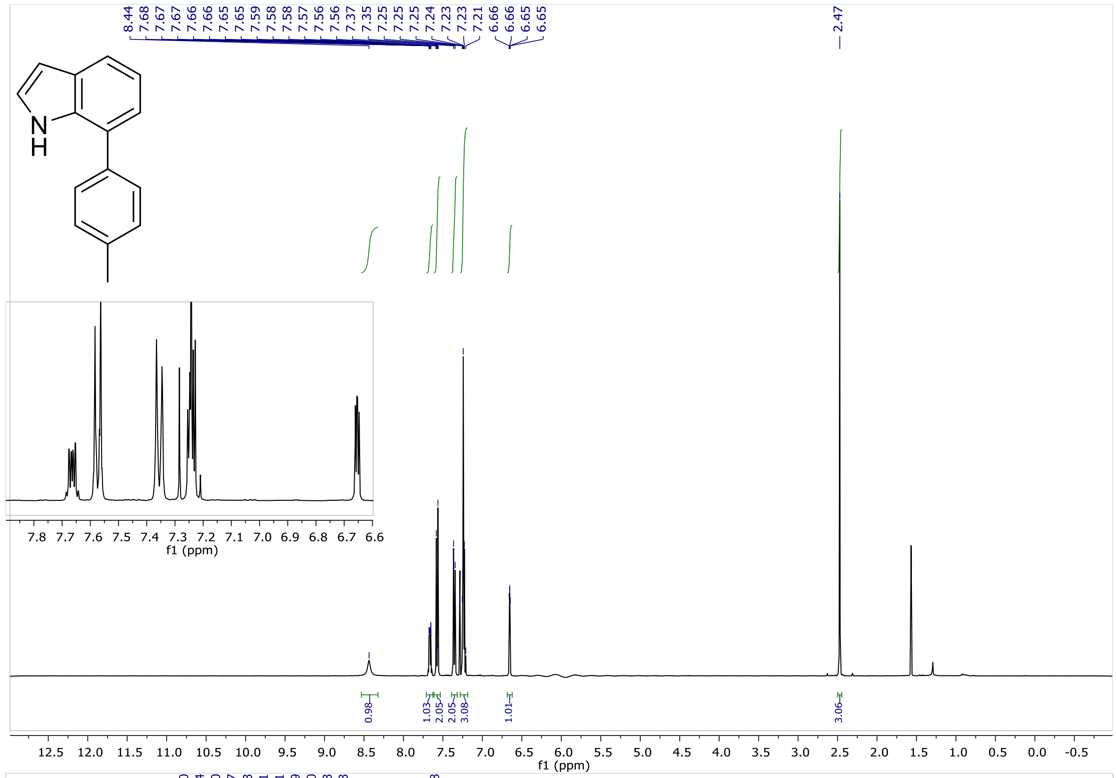

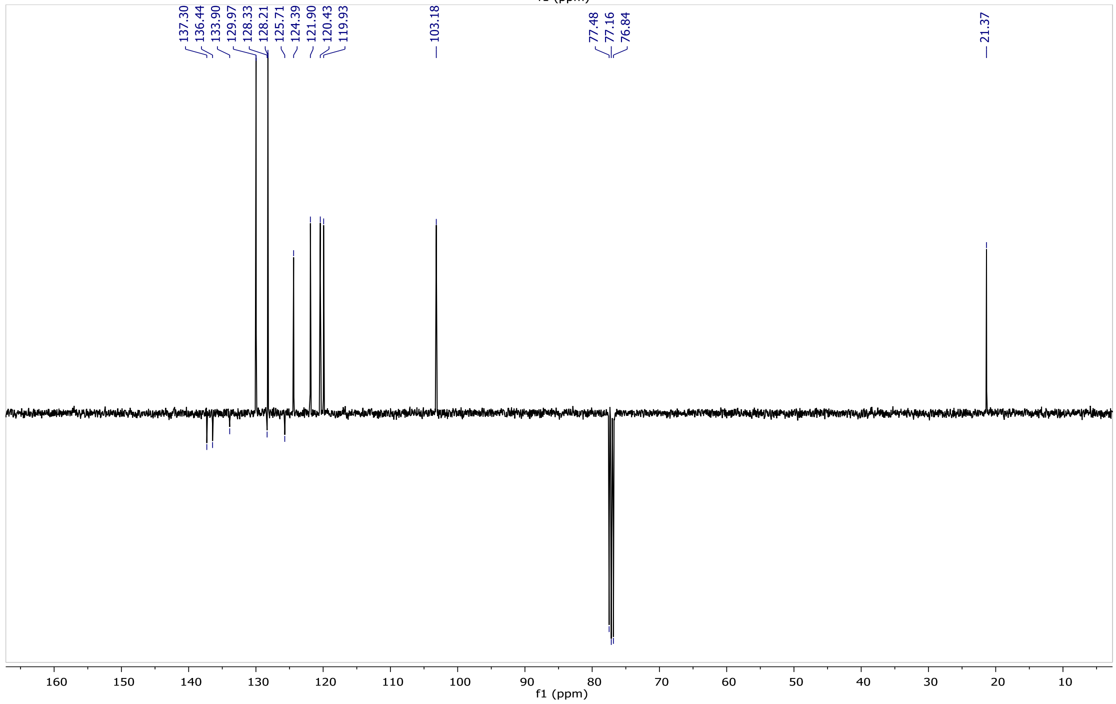

Supplementary Figure 30. ^{1}**H and** ^{13}**C NMR of 7-(*p*-tolyl)-1H-indole 7a.**

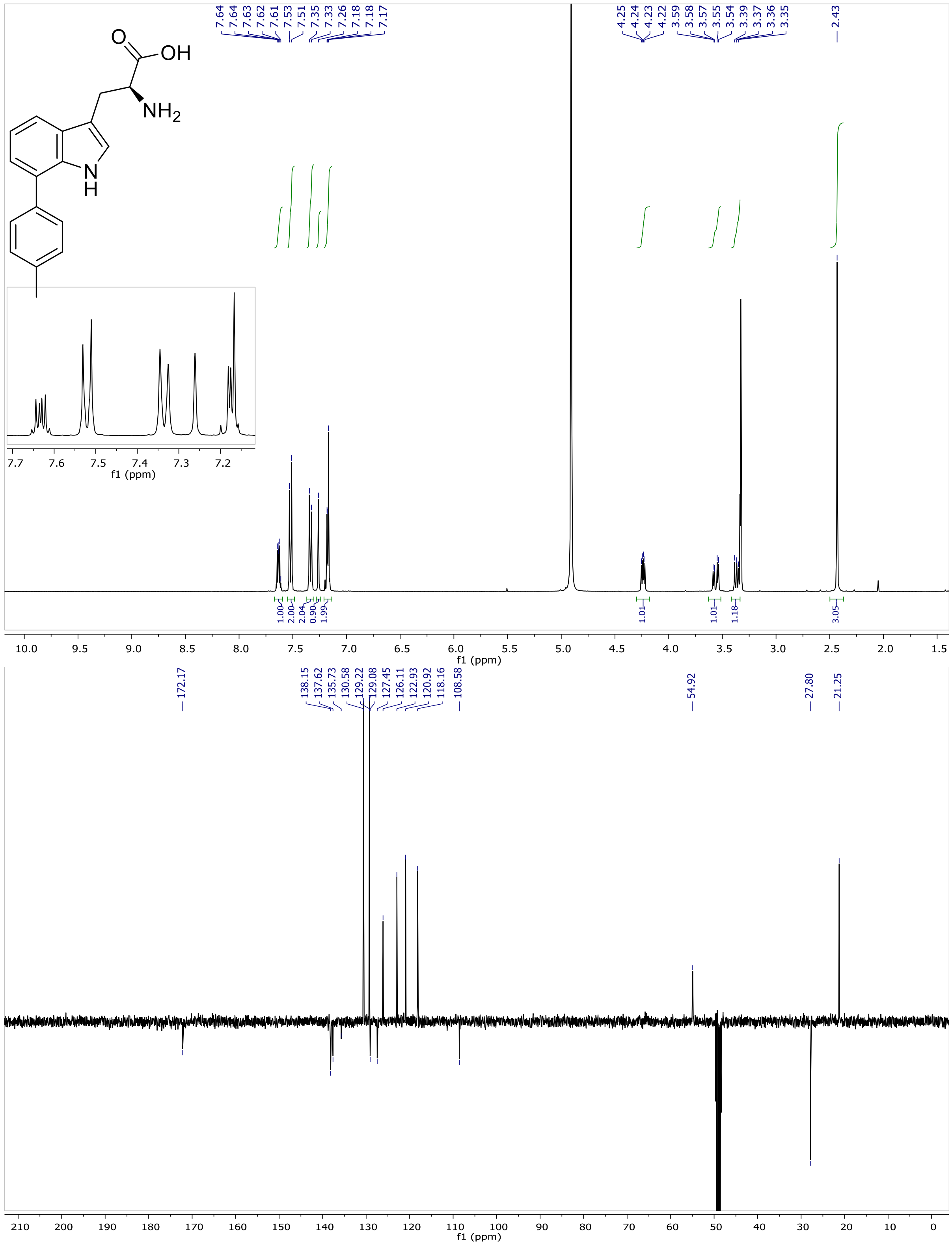

Supplementary Figure 31. ^{1}H and ^{13}C NMR of 7-(*p*-tolyl)-tryptophan 2a.

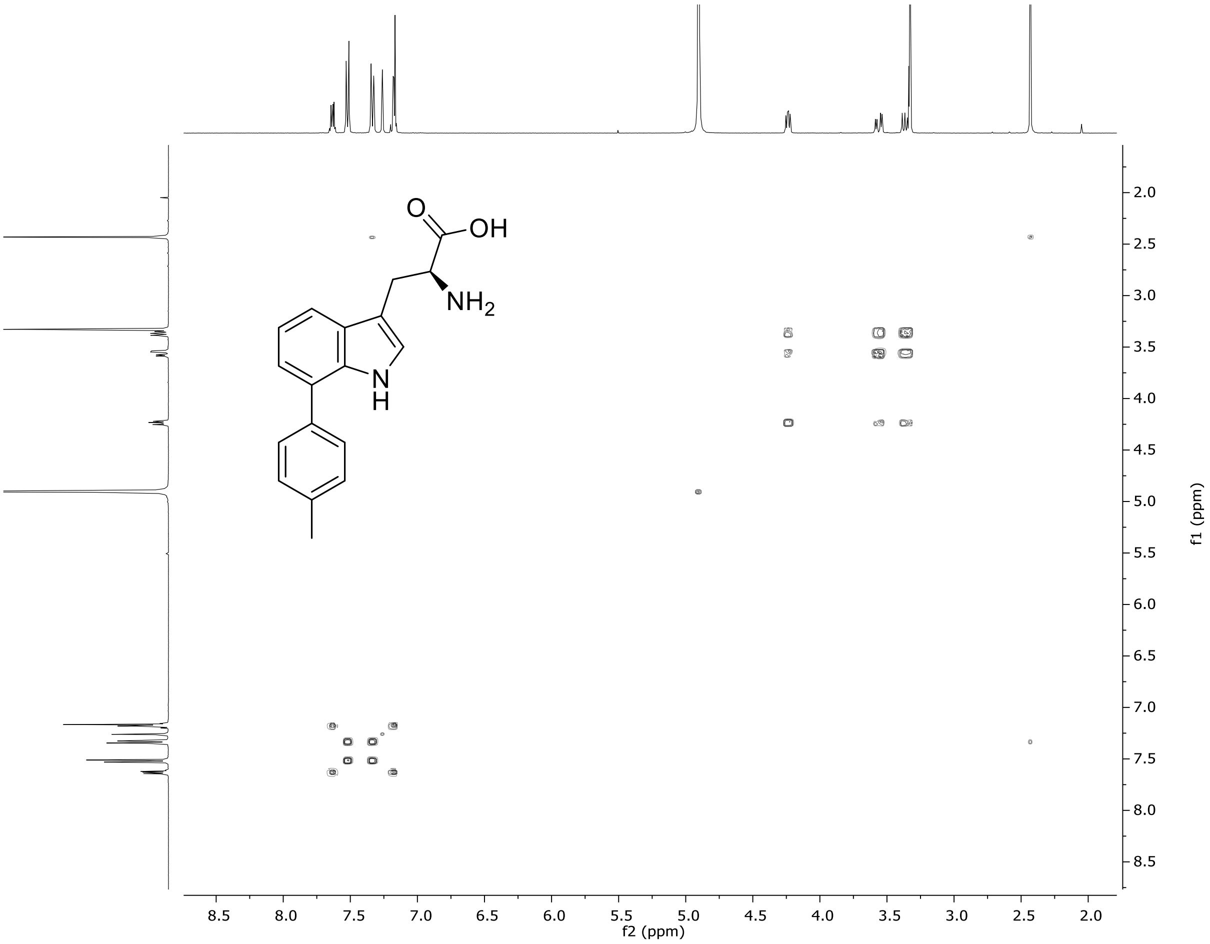

Supplementary Figure 32. COSY NMR of 7-(*p*-tolyl)-tryptophan 2a.

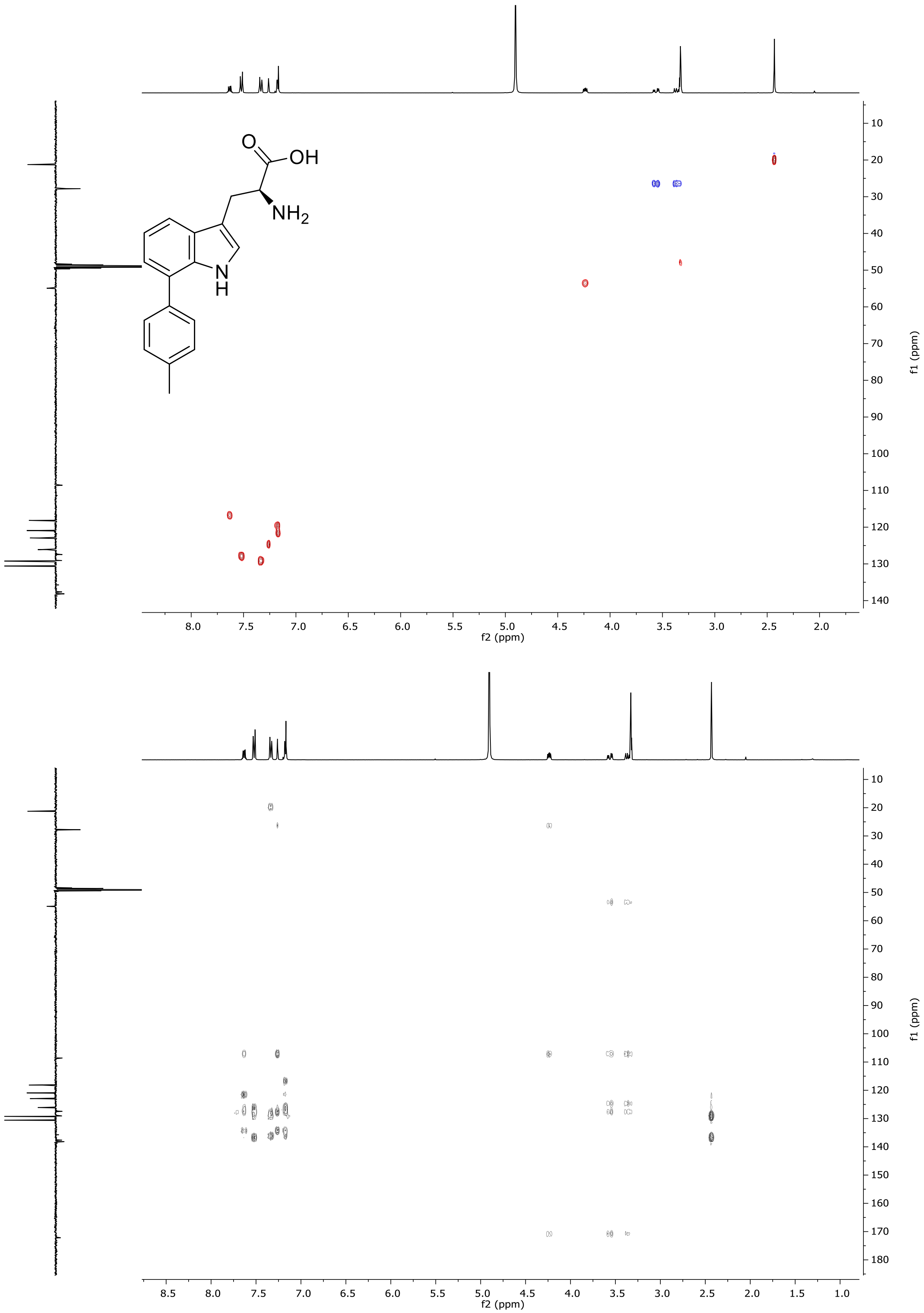

Supplementary Figure 33. HSQC and HMBC NMR of 7-(*p*-tolyl)-tryptophan 2a

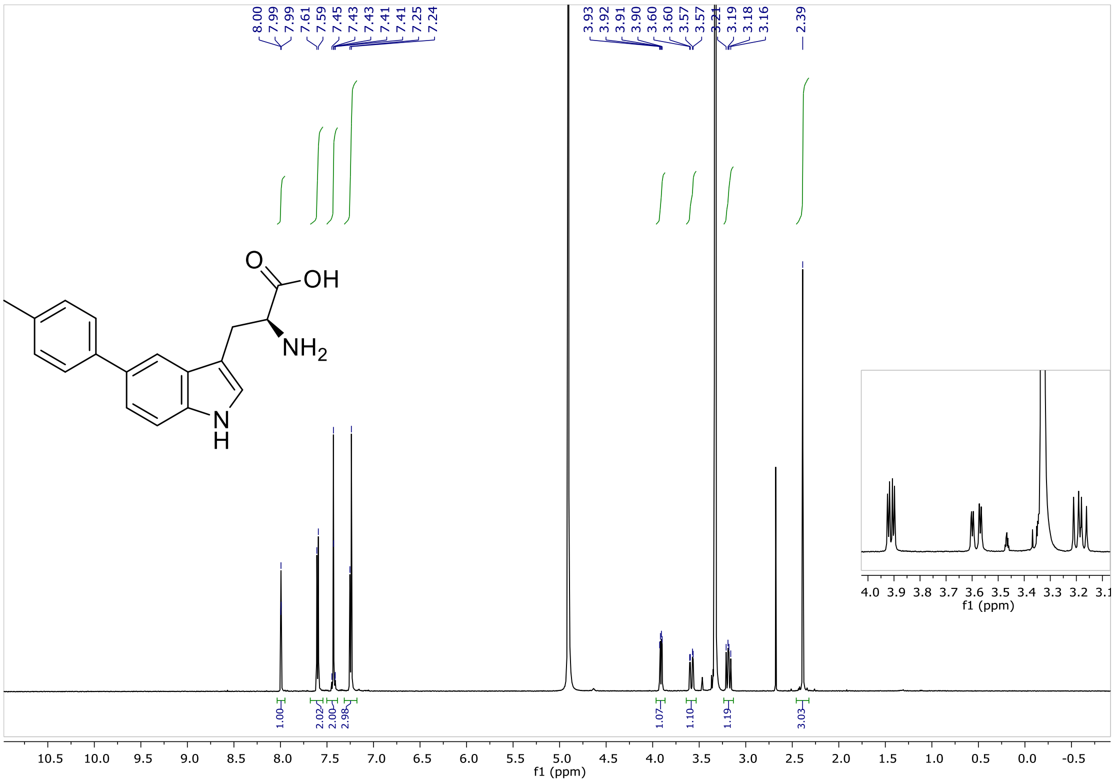

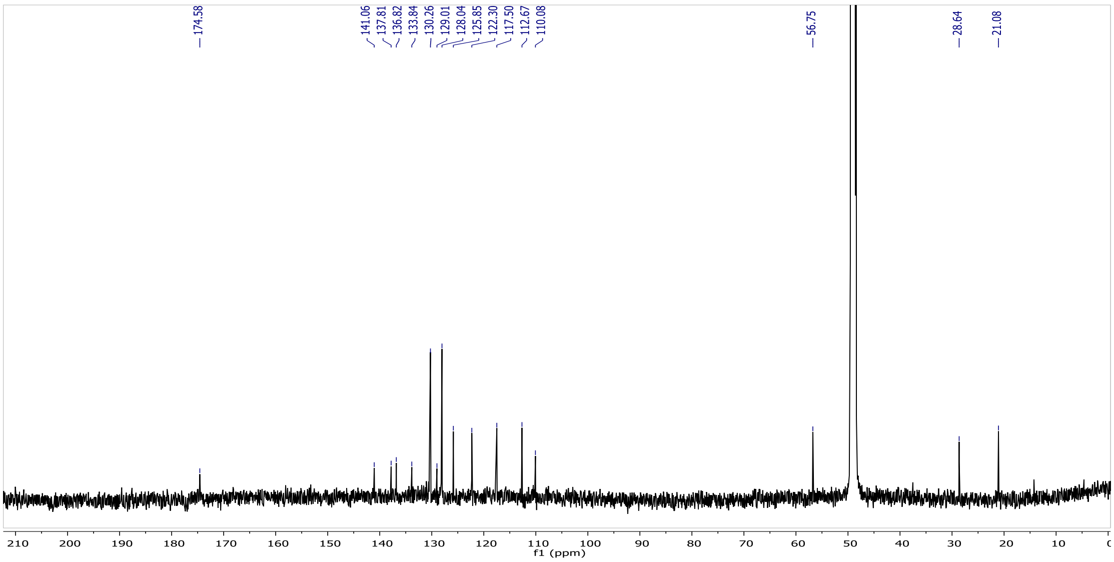

Supplementary Figure 34. ^{1}H and ^{13}C NMR of 5-(*p*-tolyl)-tryptophan 13a

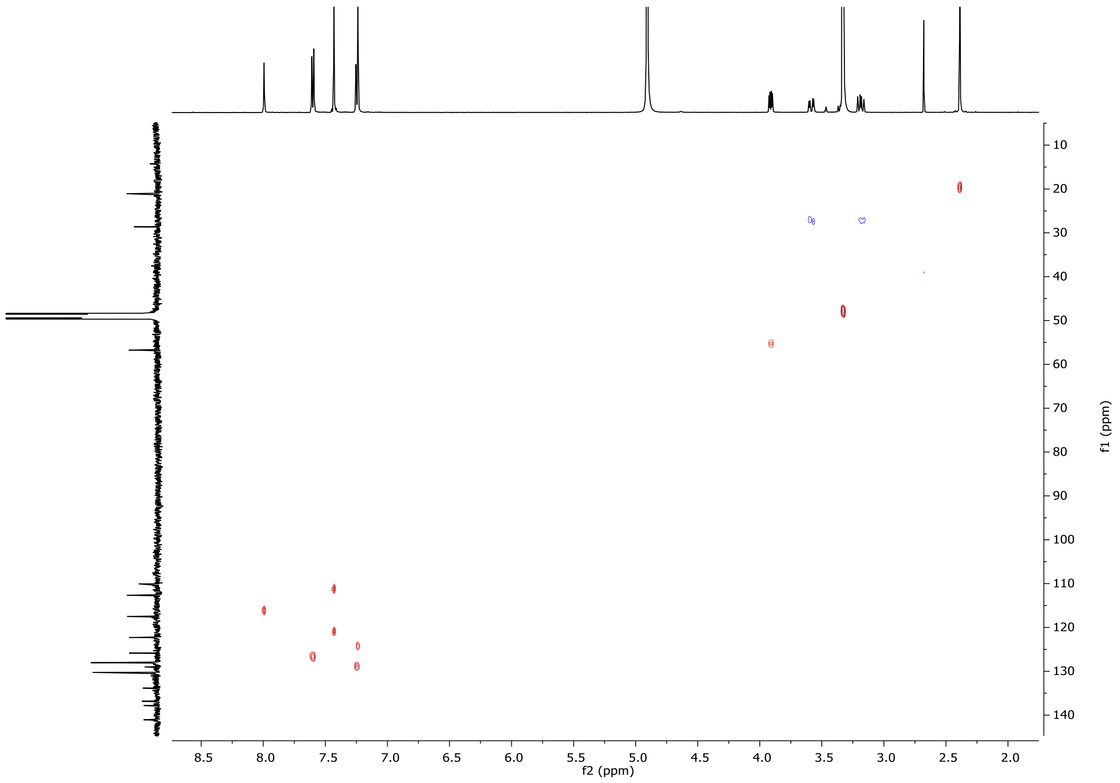

Supplementary Figure 35. HSQC NMR of 5-(*p*-tolyl)-tryptophan 13a.

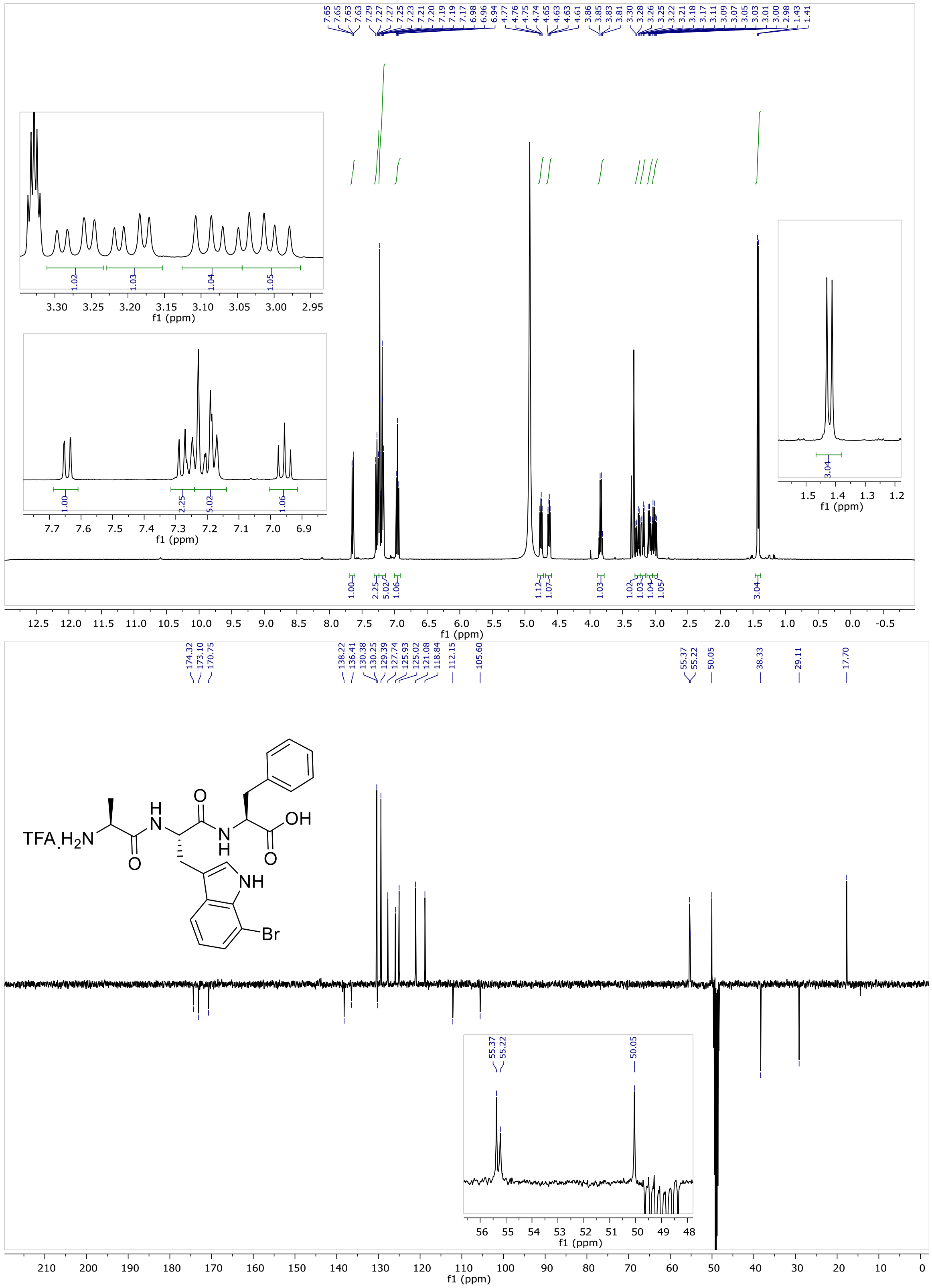

Supplementary Figure 36. ^{1}H and ^{13}C NMR of H-Ala-Trp-(7-Br)-Phe-OH 14.

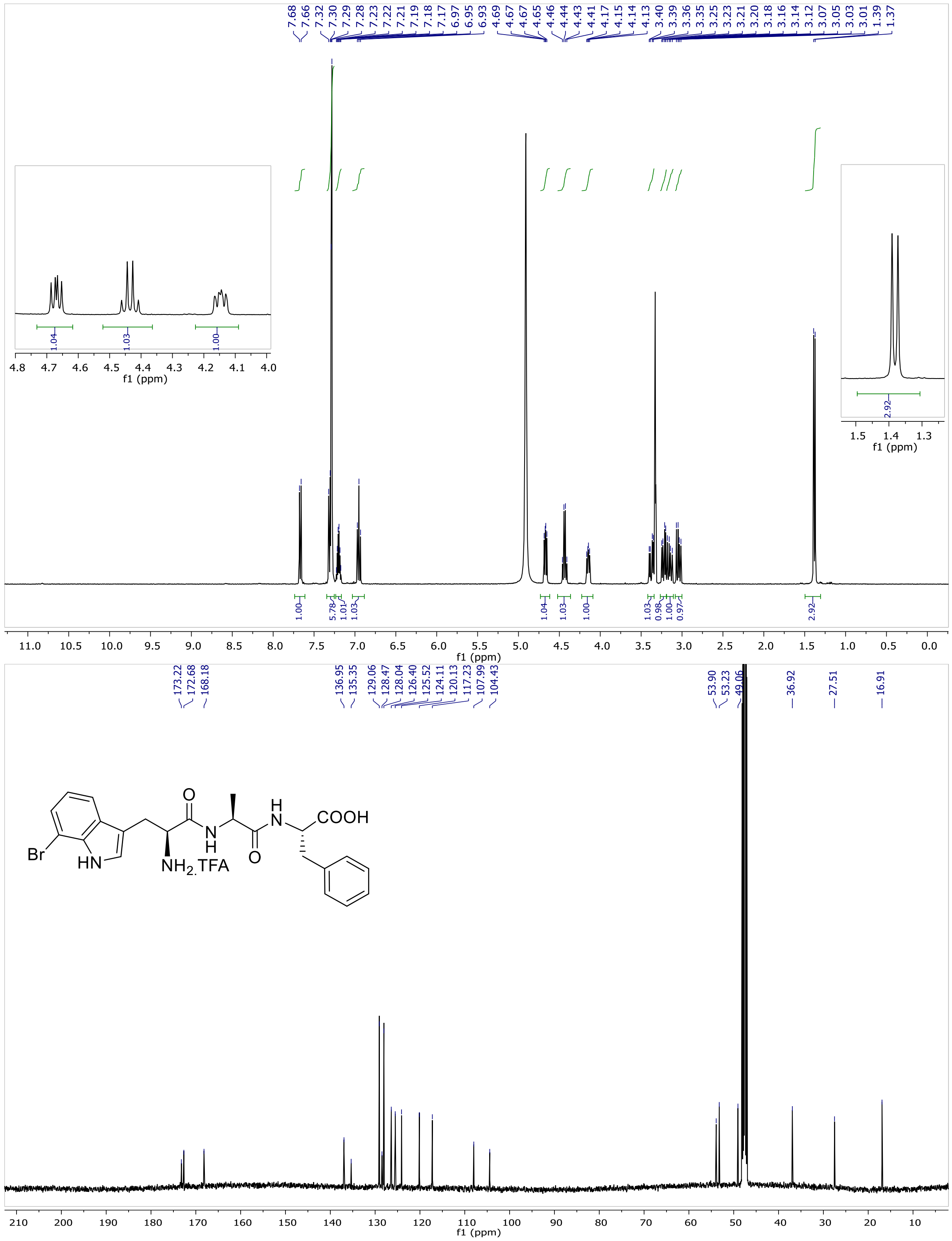

Supplementary Figure 37. ^{1}H and ^{13}C NMR of H-Trp-(7-Br)-Ala-Phe-OH 15.

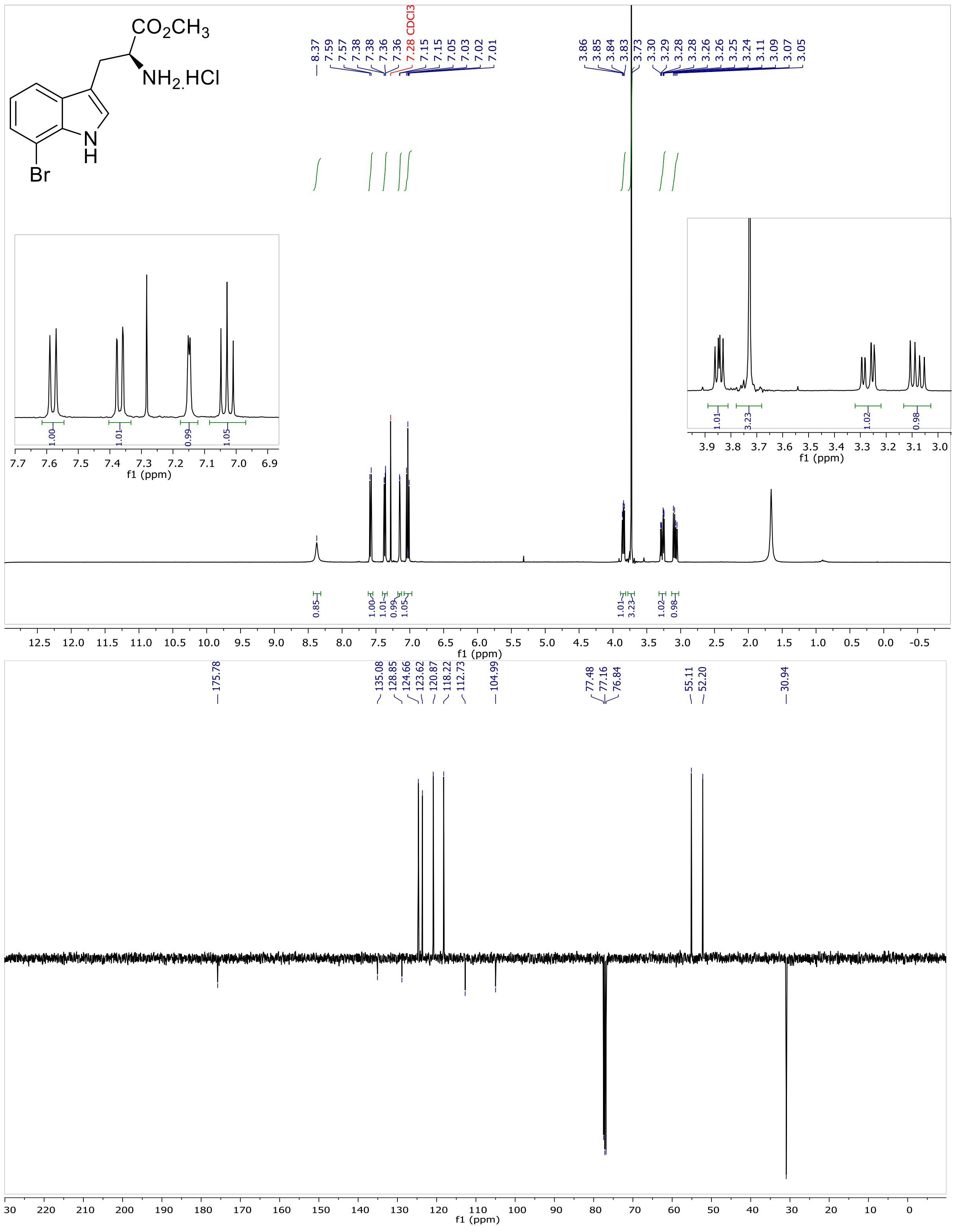

Supplementary Figure 38. ^{1}H and ^{13}C NMR of 7-Br-tryptophan methyl ester.

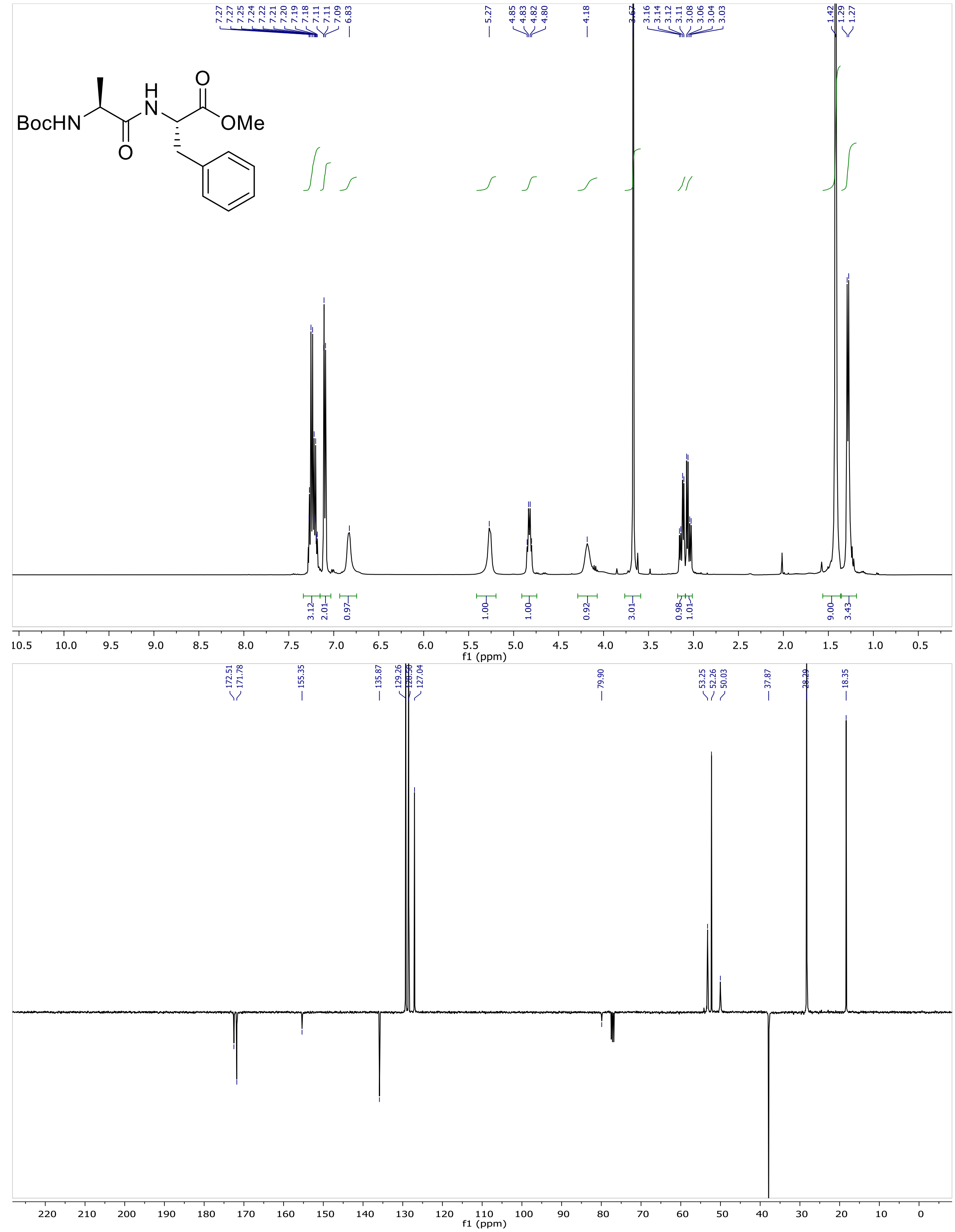

Supplementary Figure 39. ^{1}H and ^{13}C NMR of *N*-Boc-Ala-Phe-OMe

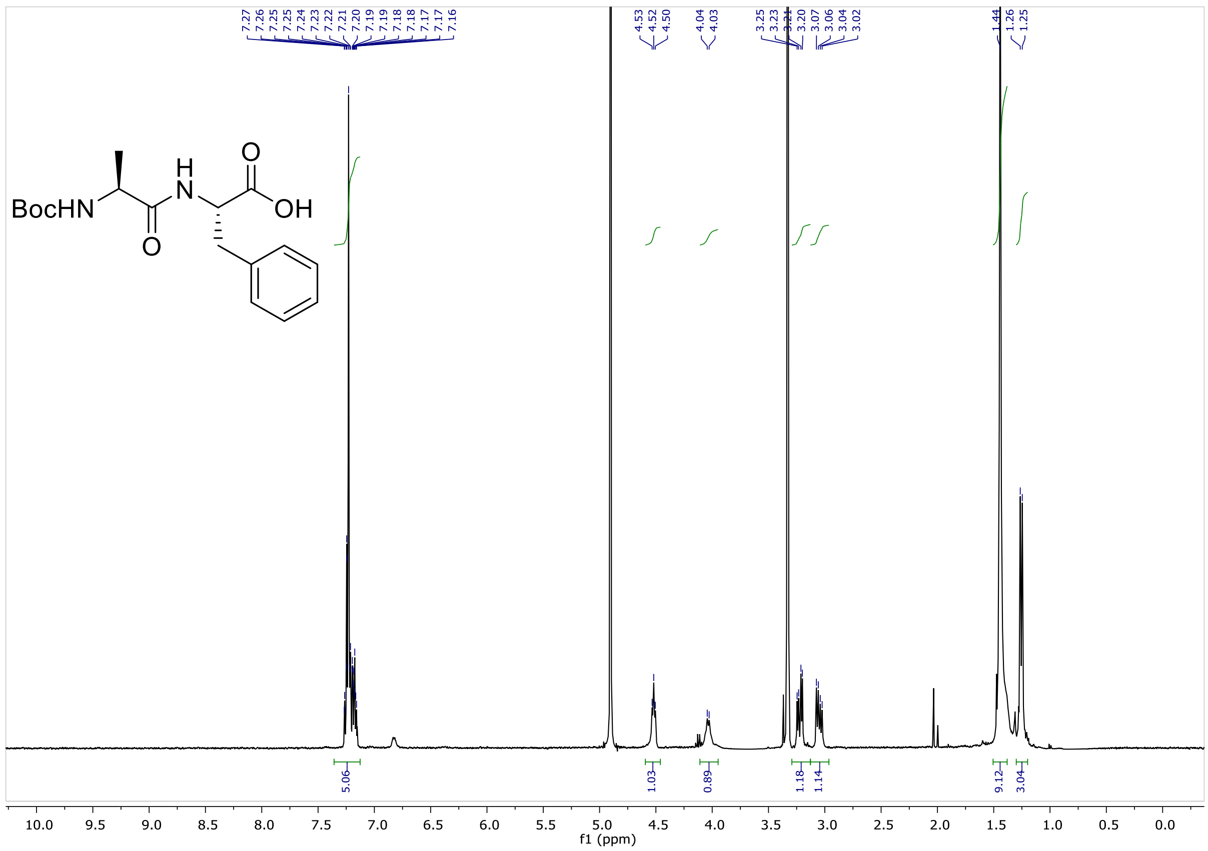

Supplementary Figure 40. ^{1}H and ^{13}C NMR of *N*-Boc-Ala-Phe-OH

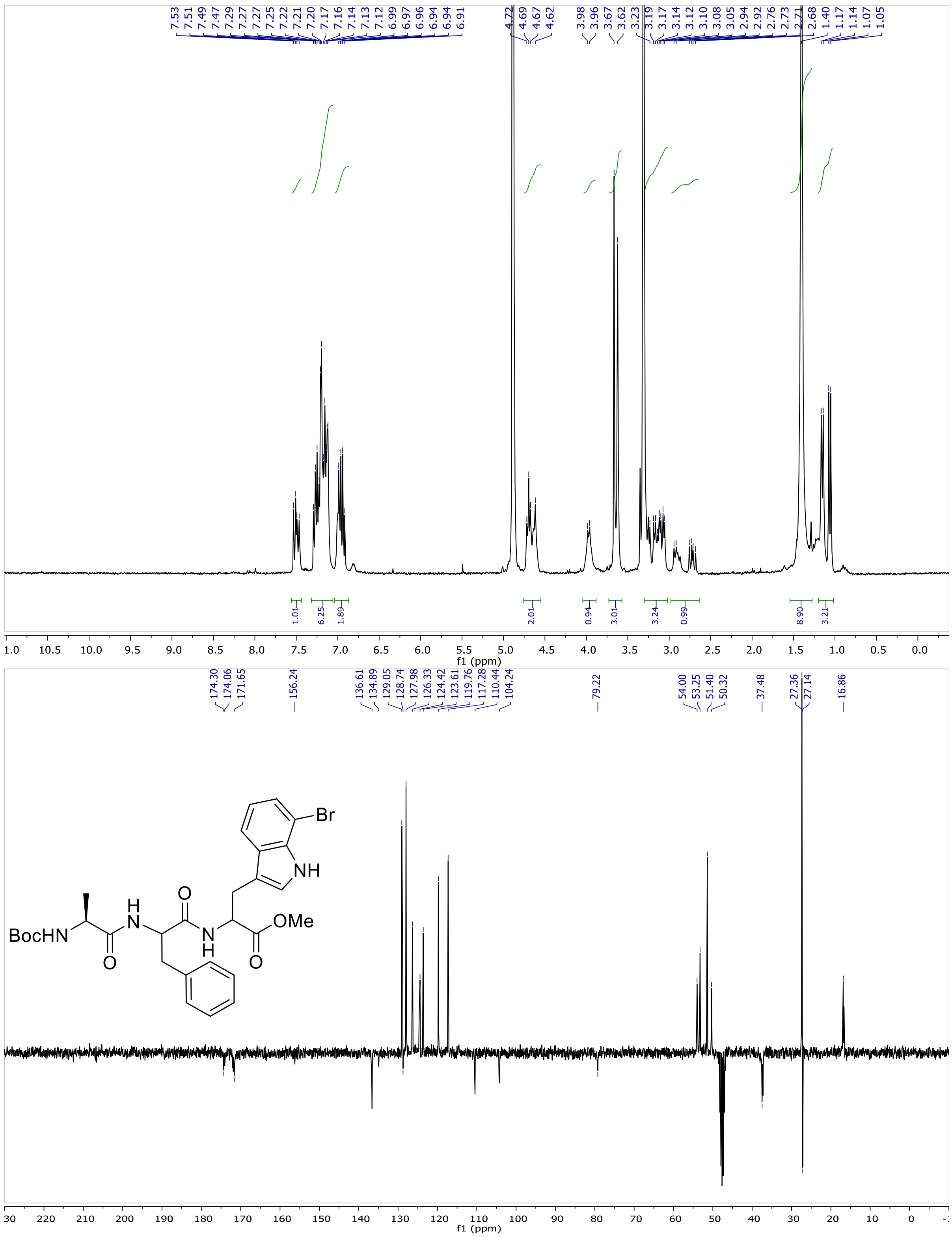

Supplementary Figure 41. ^{1}H and ^{13}C NMR of *N*-Boc-Ala-Phe-Trp-(7-Br)-OMe.

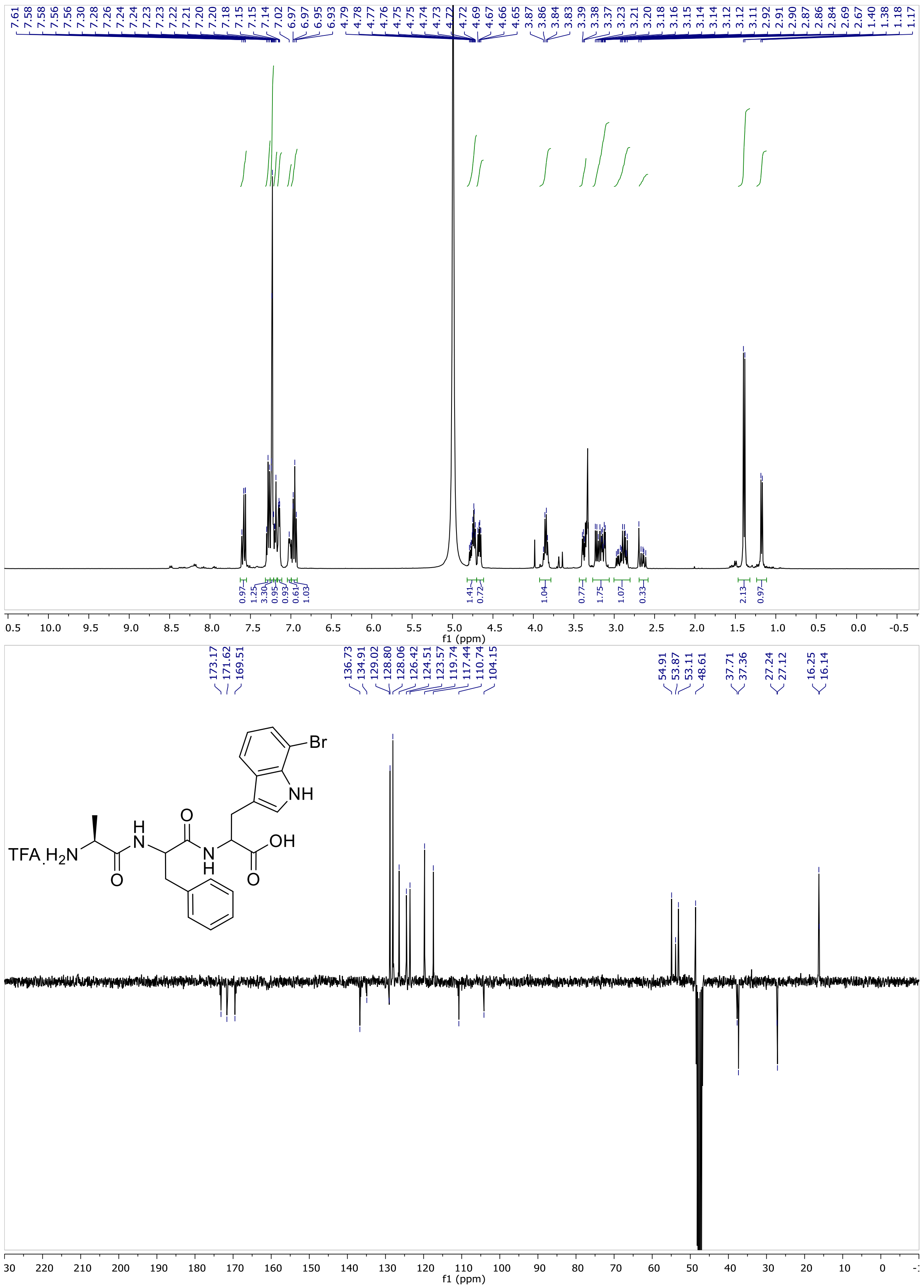

Supplementary Figure 42. ^{1}H and ^{13}C NMR of the diastereomeric mixture of H-Ala-Phe-Trp-(7-Br)-OH 16.

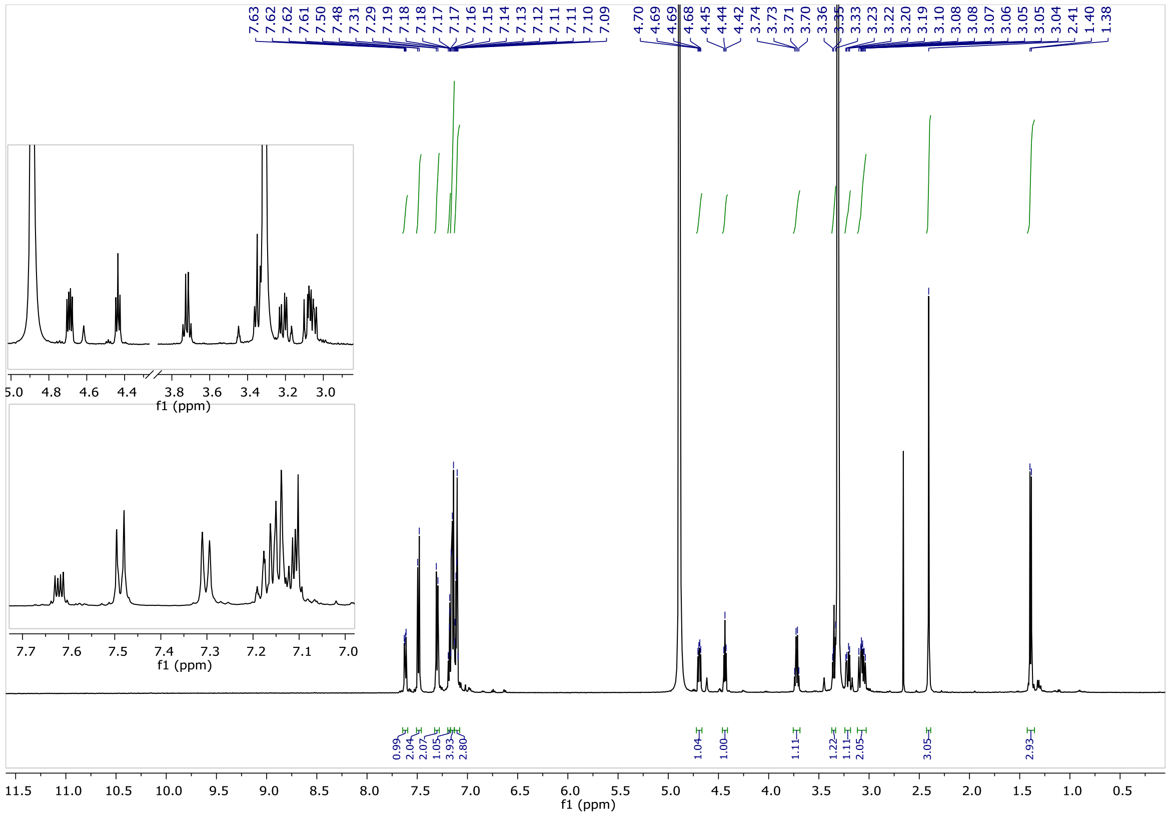

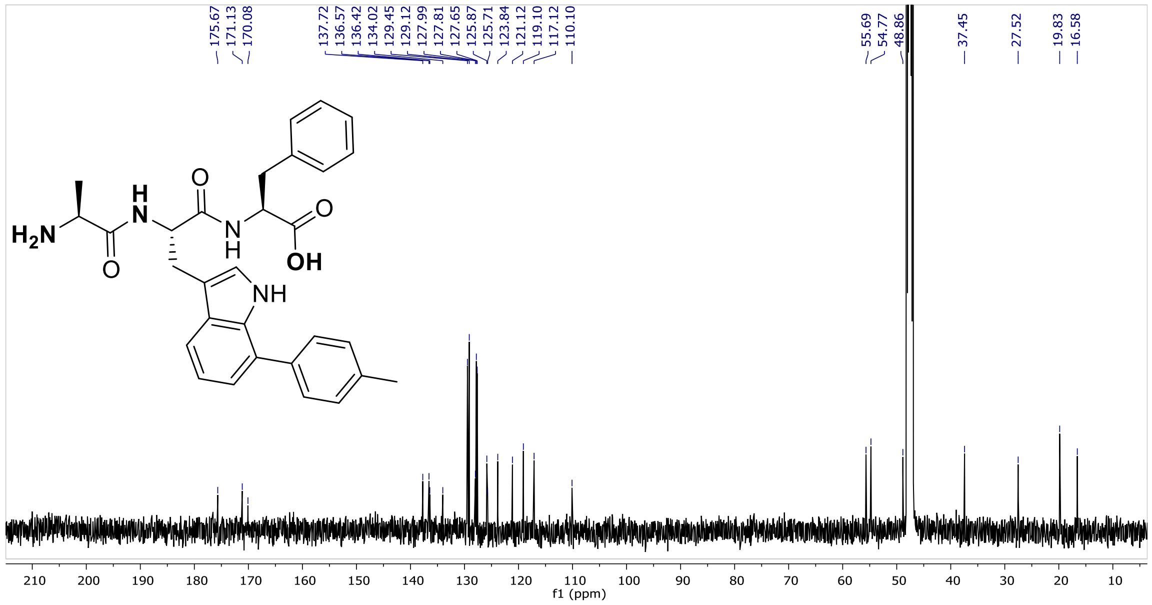

Supplementary Figure 43. ^{1}H and ^{13}C NMR of H-Ala-Trp-(7-*p*-tol)-Phe-OH 14a.

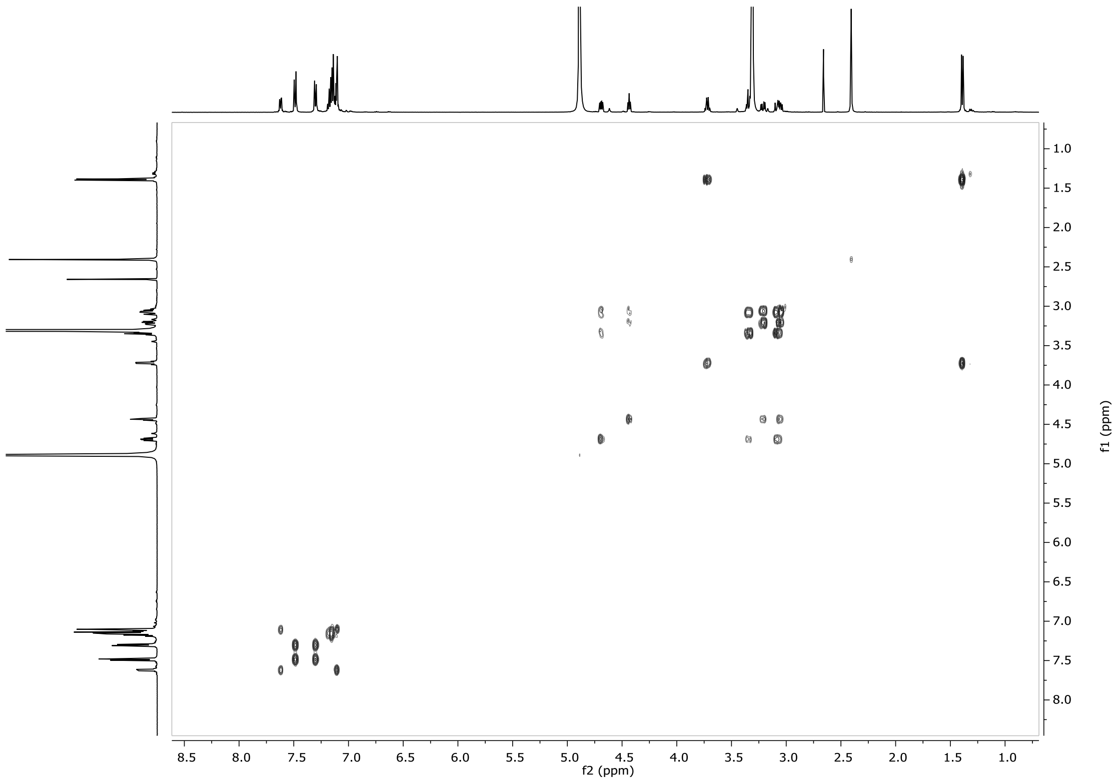

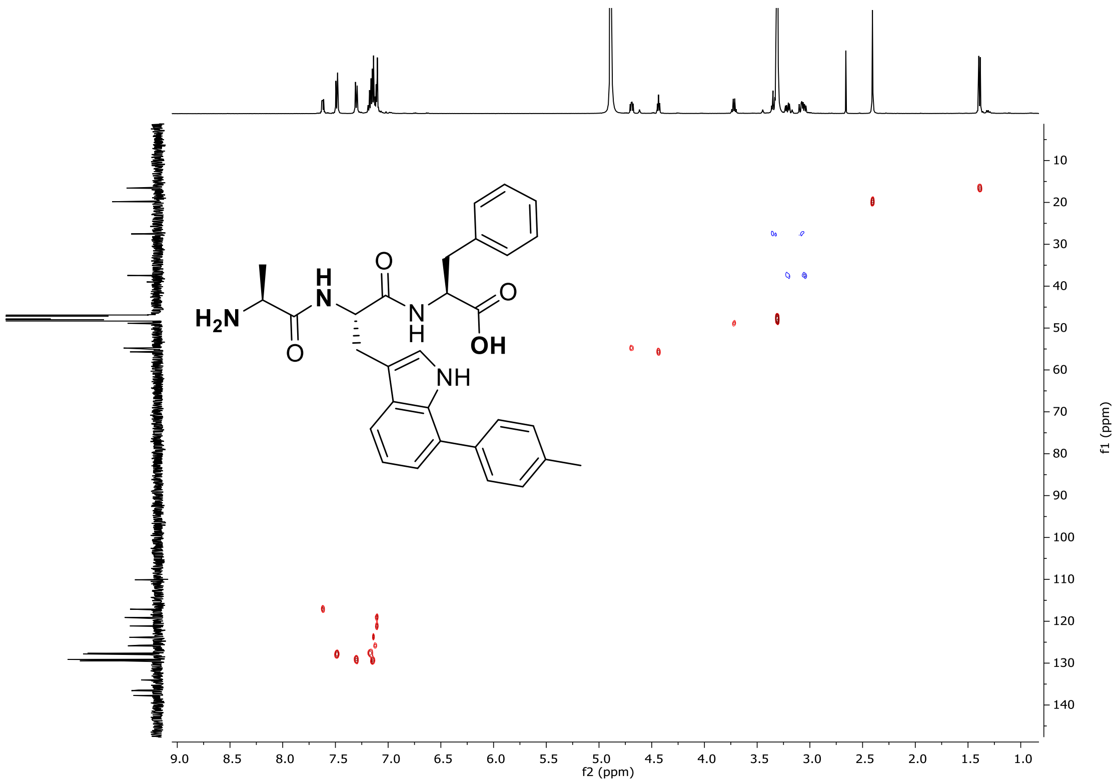

Supplementary Figure 44. COSY and HSQC NMR of H-Ala-Trp-(7-*p*-tol)-Phe-OH 14a.

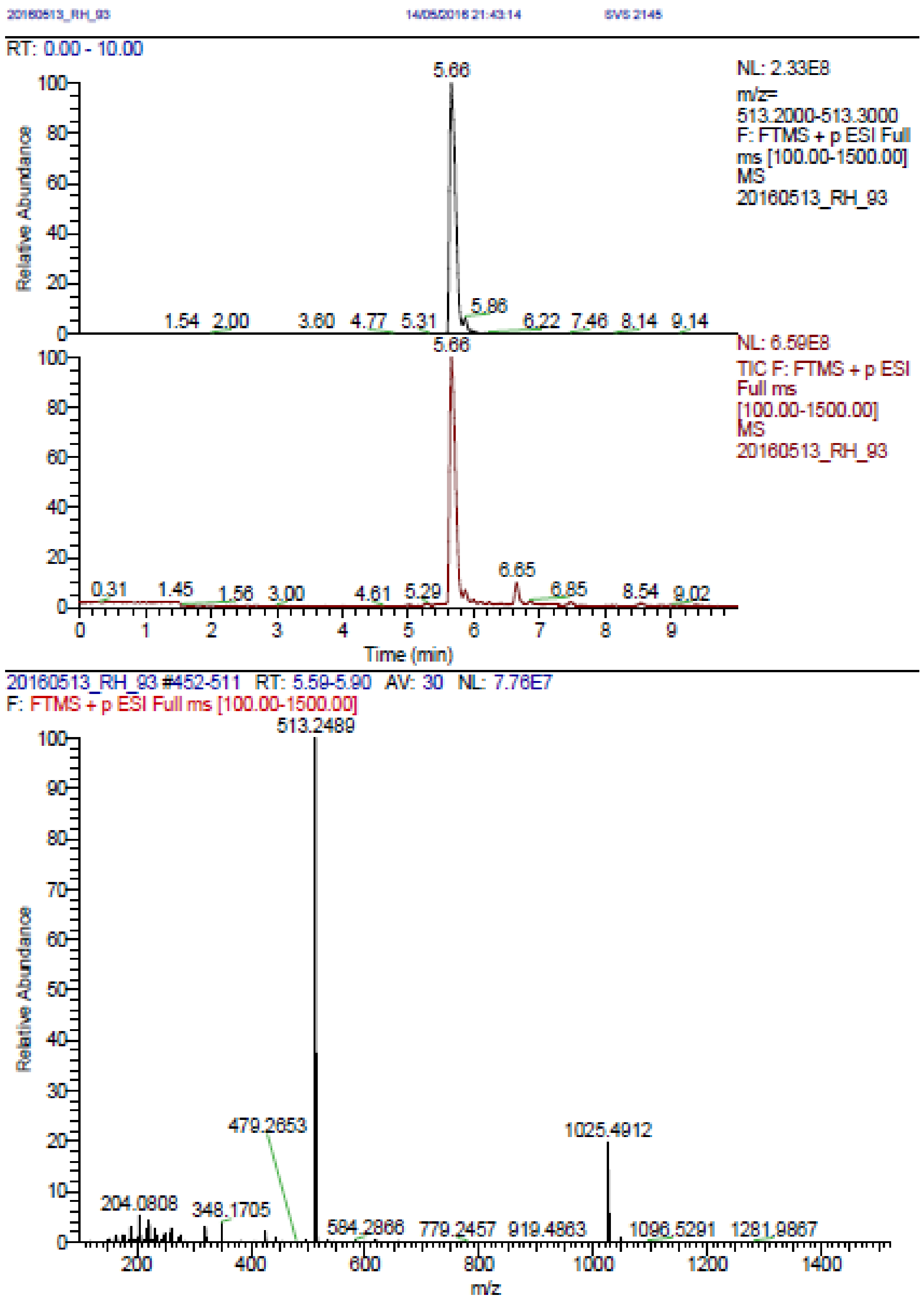

Supplementary Figure 45. LC-HRMS analysis of H-Ala-Trp-(7-*p*-tol)-Phe-OH 14a.

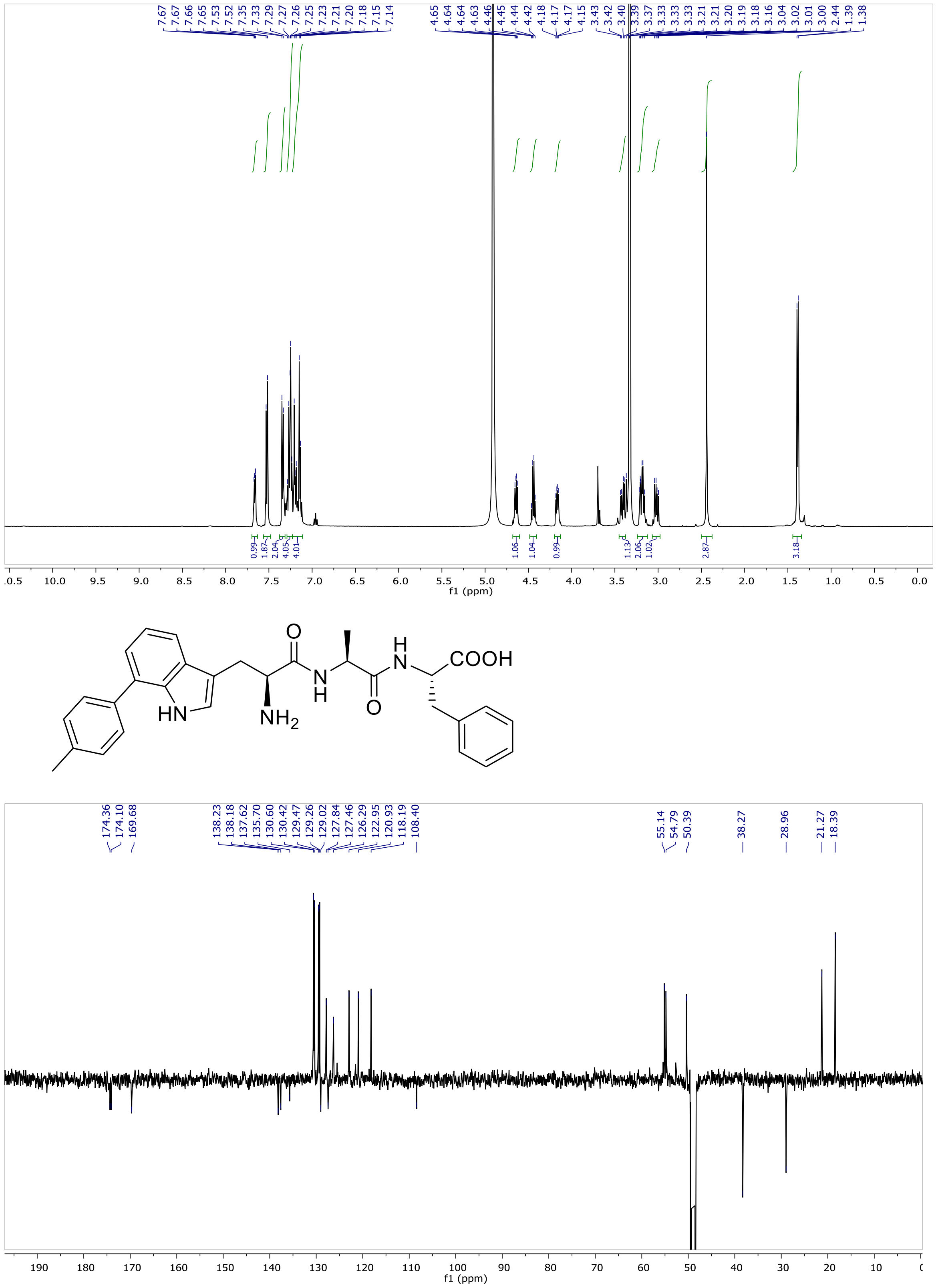

Supplementary Figure 46. ^{1}**H and** ^{13}**C NMR of H-Trp-(7-*p*-tol)-Ala-Phe-OH 15a.**

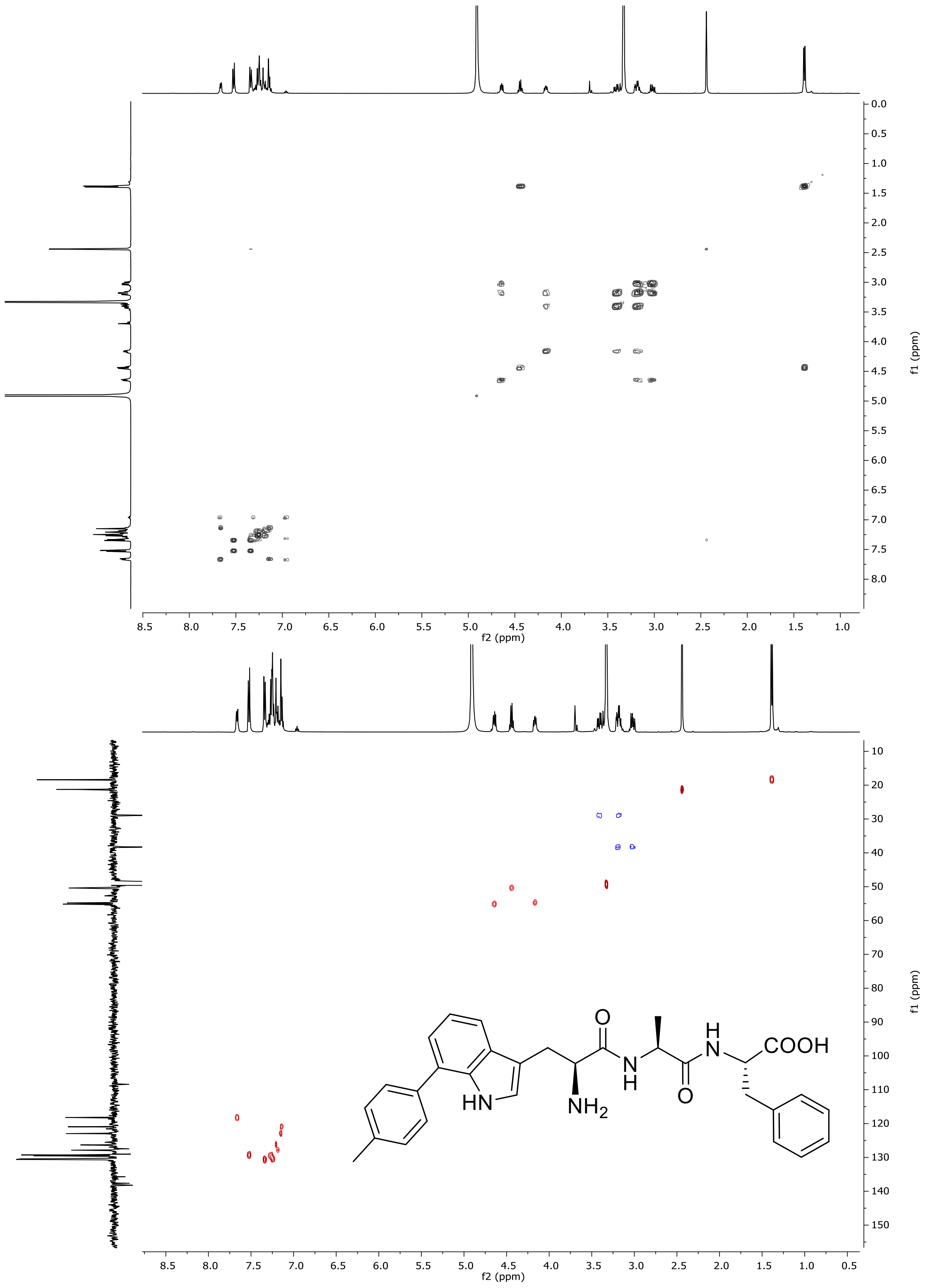

Supplementary Figure 47. **COSY and HSQC NMR of H-Trp-(7-*p*-tol)-Ala-Phe-OH 15a.**

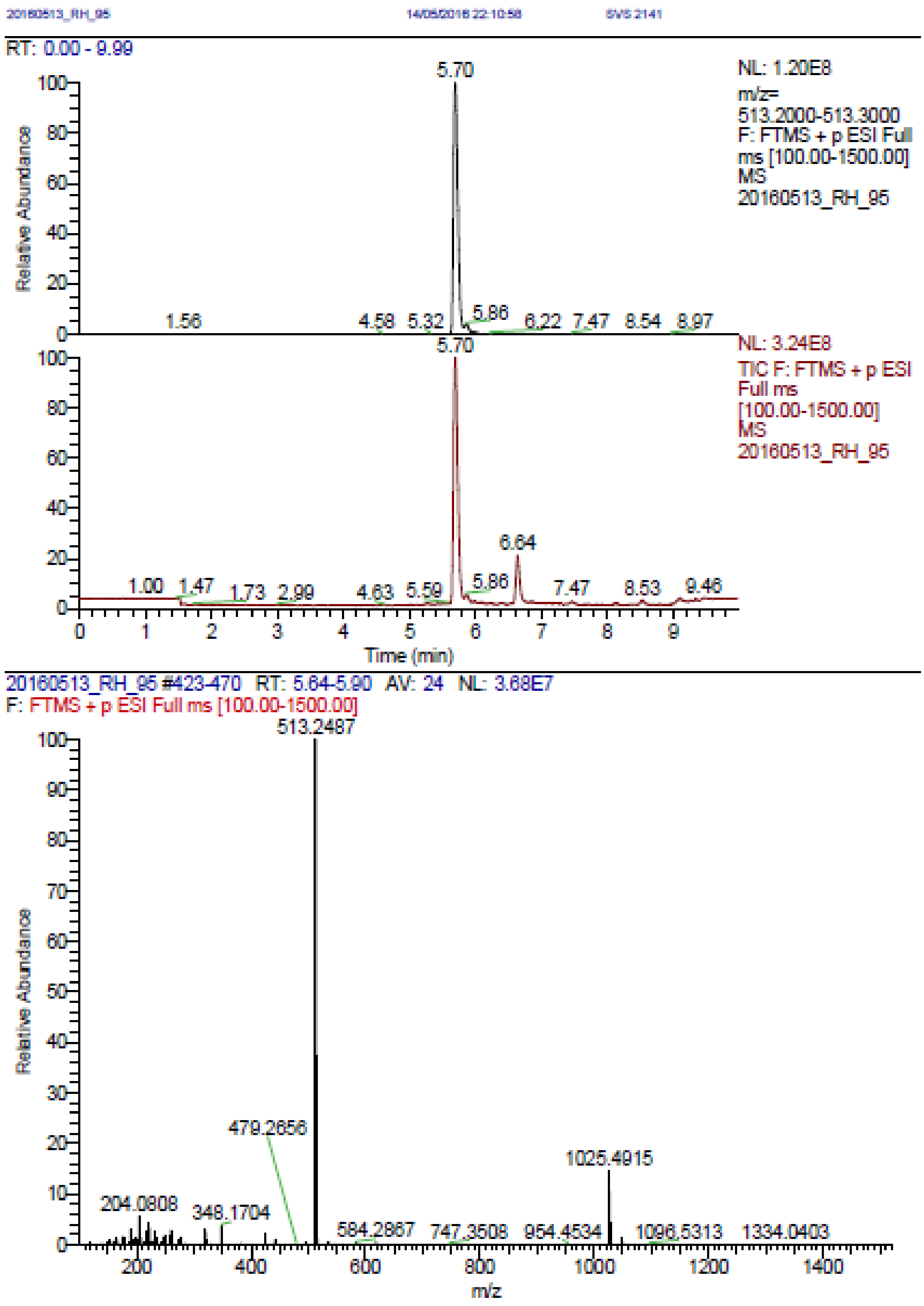

Supplementary Figure 48. LC-HRMS analysis of H-Trp-(7-*p*-tol)-Ala-Phe-OH 15a.

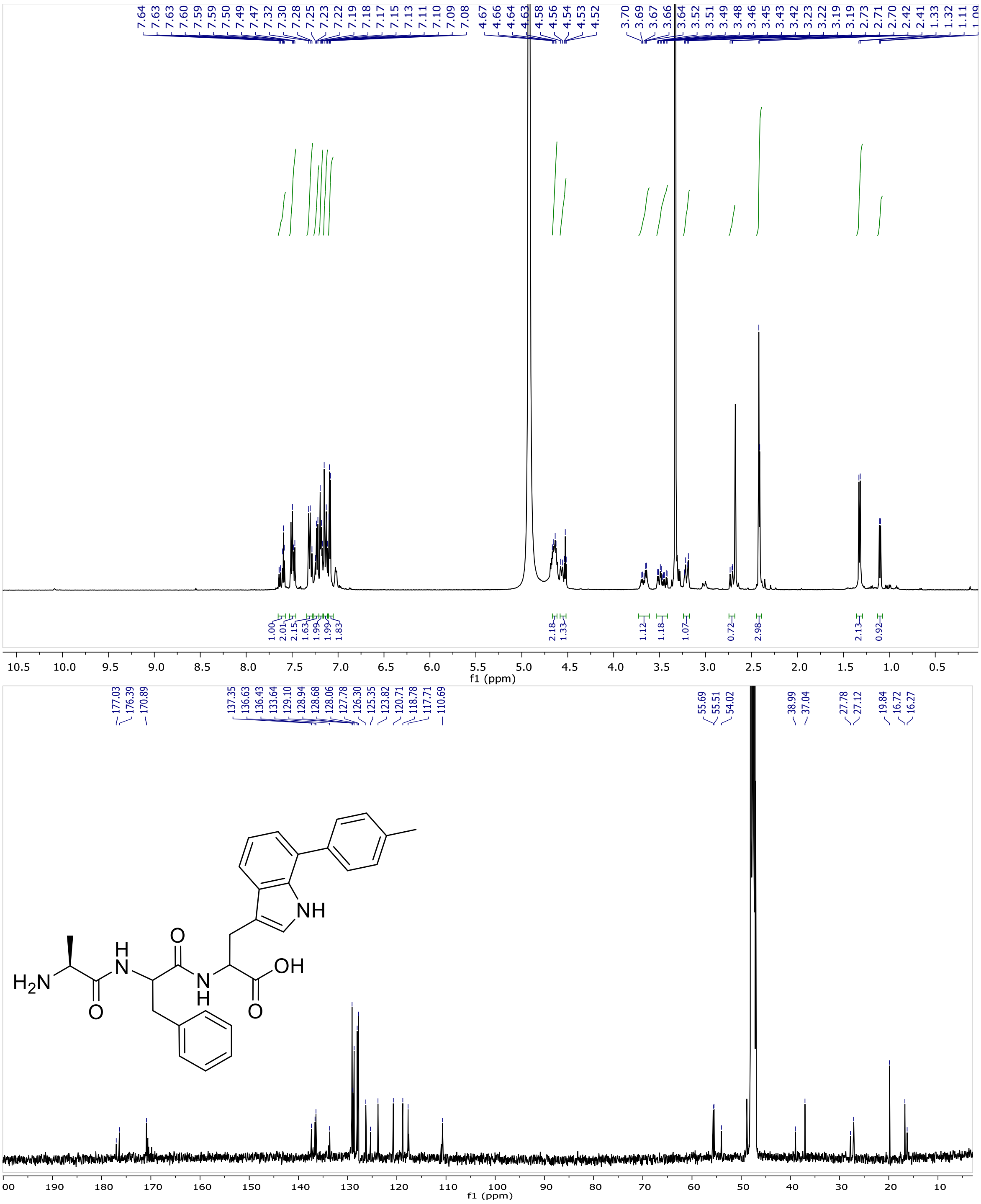

Supplementary Figure 49. ^{1}H and ^{13}C NMR of diastereomeric mixture of H-Ala-Phe-Trp-(7-*p*-tol)-OH 16a.

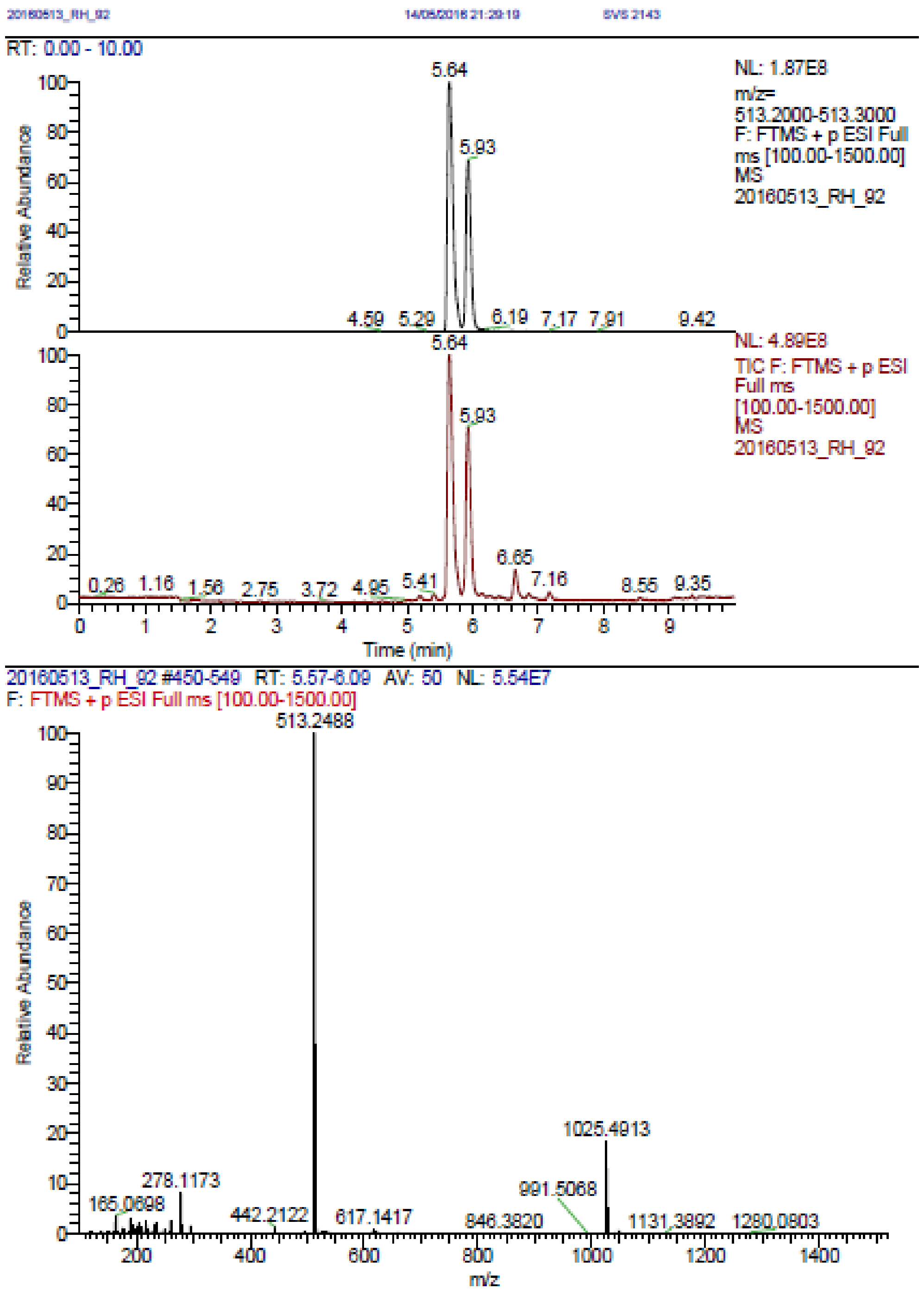

Supplementary Figure 50. LC-HRMS analysis of diastereomeric mixture of H-Ala-Phe-Trp-(7-*p*-tol)-OH 16a.

SUPPLEMENTARY TABLES

Supplementary Table 1. Initial screening of Pd catalyst, base and solvent for Suzuki-Miyaura cross-coupling for various 5-halo-indoles with *p*-Tol-B(OH)₂.

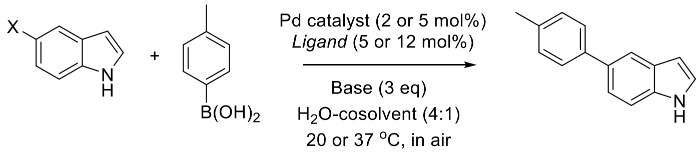

Entry	X	Pd-Ligand (1:2.5 ratio)	Base	Solvent	Time (Temp.)	Conversion (%)[a]
Solvent screen						
1	I (**4**)	Na_2PdCl_4-^{S}SPhos (2 mol%)	K_2CO_3	Water	8 h (20 °C)	72
2	I (**4**)	Na_2PdCl_4-^{S}SPhos (2 mol%)	K_2CO_3	Water-EtOH (4:1)	8 h (20 °C)	92
3	I (**4**)	Na_2PdCl_4-^{S}SPhos (2 mol%)	K_2CO_3	Water-CH_3CN (4:1)	8 h (20 °C)	90
Base screen						
4	I (**4**)	Na_2PdCl_4-^{S}SPhos (2 mol%)	K_2CO_3	Water-CH_3CN (4:1)	8 h (37 °C)	98
5	I (**4**)	Na_2PdCl_4-^{S}SPhos (2 mol%)	Na_2CO_3	Water-CH_3CN (4:1)	8 h (37 °C)	58
6	I (**4**)	Na_2PdCl_4-^{S}SPhos (2 mol%)	Cs_2CO_3	Water-CH_3CN (4:1)	8 h (37 °C)	70
7	I (**4**)	Na_2PdCl_4-^{S}SPhos (2 mol%)	Na_3PO_4	Water-CH_3CN (4:1)	8 h (37 °C)	85
Catalyst screen						
8	Br (**5**)	Na_2PdCl_4-^{S}SPhos (5 mol%)	K_2CO_3	Water-CH_3CN (4:1)	18 h (37 °C)	99
9	Br (**5**)	Na_2PdCl_4-^{S}XPhos (5 mol%)	K_2CO_3	Water-CH_3CN (4:1)	18 h (37 °C)	20
10	Br (**5**)	Na_2PdCl_4-TPPTS (5 mol%)	K_2CO_3	Water-CH_3CN (4:1)	18 h (37 °C)	48
11	Br (**5**)	(dtbpf)$PdCl_2$ (5 mol%)	K_2CO_3	Water-CH_3CN (4:1)	18 h (37 °C)	65
12	Br (**5**)	Xantphos.$PdCl_2$ (5mol%)	K_2CO_3	Water-CH_3CN (4:1)	18 h (37 °C)	25

Conditions: A mixture of 5-halo-indole (0.1 mmol), Pd-catalyst (2 or 5 mol%), ligand (5 or 12 mol%), *p*-Tol-B(OH)₂ (0.15 mM) and appropriate base (0.3 mmol) in corresponding solvent (2 ml) was stirred at specified temperature. [a]Conversion was determined by ^{1}H NMR of the crude reaction.

Supplementary Table 2. Preliminary screening of various culture media for cross-coupling of 7-Br-tryptophan 2.

Media/buffer	Conversion (%)[a,c]	Media/Buffer	Conversion (%)[b,c]
Phosphate (pH 8, 100mM)+NaCl (50mM)	40	Trisodium citrate (97 mM, 2.5% *w/v*)	50
Phosphate (pH 8, 100mM)+ Ammonium sulfate (25 mM)	trace	Trisodium citrate (19.4 mM, 0.5% *w/v*)	75
Phosphate (pH 8, 100mM)+ Ammonium sulfate (2.5 mM)	~8	Medium-B (with 180mM NaCl)	30
Phosphate (pH 8, 100mM)+ Ammonium chloride (25 mM)	trace	Medium-B (with 180mM NaBr)	30
Phosphate (pH 8, 10mM)	50	Glucose (22 mM, 0.4% *w/v*)	10
Phosphate (pH 8, 10mM)+Trace metals (1x)	37	Glycerol (217 mM, 2% *w/v*)	10
LB, 2xTY, ISP-2, INA-2, PPM, LMR, NC, M9, SPG, Yeast extract (0.4% *w/v*), Malt extract (1% *w/v*), M63			No or trace conversion (<2%) to product was detected

Conditions: A mixture of 7-Br-tryptophan **2** (350 µM), **L2-Pd** (100 µM), *p*-Tol-B(OH)$_2$ (1.2 mM) in corresponding buffer or media (2 ml, final pH 7.5-8.5 adjusted using 100 mM K_2CO_3 if necessary) was stirred at 32 °C. [a]Determined at 24 h, [b]determined at 48 h, [c]conversion was determined by LCMS analysis using the extracted ion peak areas for substrate **2** and the product **2a**.

Supplementary Table 3. Screening of selected culture media for cross-coupling of 7-Br-tryptophan 2 at 1:4 dilution in phosphate buffer at 37 °C.

Media (0.5 ml) + Phosphate buffer (1.5 ml, pH 8.5, 10 mM)	Conversion (%)[a]
Medium B-A	75
M-5	80
M-9	55
M-4	<5

Conditions: A mixture of 7-Br-tryptophan **2** (350 µM), **L2-Pd** (100 µM), *p*-Tol-B(OH)$_2$ (5 mM) in corresponding buffer or media (2 ml, final pH was adjusted to 7.5-8.5 using 500 mM K_2CO_3 if necessary) was stirred at 37 °C for 60 h. [a]Conversion was determined after the UPLC analysis (UPLC method 1) using the product **2a** fluorescence peak area.

Supplementary Table 4. Cross-coupling of 7-Br-tryptophan 2 (generated *in vivo* by bromination of L-tryptophan 1 in cultures of *E. coli*) in spent culture media at different dilutions.

Spent Media (volume, ml)	Dilution to (with phosphate buffer, pH 8.5, 10 mM)	Spent media-B-A[a] Conversion (%)[c]	Spent CCM[b] Conversion (%)[c]
1	50%	1	-
0.66	33%	3	55
0.5	25%	8	71
0.4	20%	14	82
0.2	10%	52	95

Conditions: Appropriate volume of cell free spent media containing 7-Br-tryptophan **2**, generated *in vivo*, **L2-Pd** (100 µM), *p*-Tol-B(OH)$_2$ (1.2 mM) in phosphate buffer (total volume 2 ml) was stirred at 37 °C for 60 h. [a]Concentration of **2** was 0.25 mM, [b]concentration of **2** was 0.28 mM, [c]conversion was determined after the UPLC analysis (UPLC method 1) using the product **2a** fluorescence peak area.

Supplementary Table 5: Cross-coupling of *in vivo* generated 7-Br-tryptophan 2 in living *E. coli* RG-1500 culture.

Sample Id	Fluorescence peak area @4.0 min Ex295/Em370 (after 50% dilution with methanol)	Concentration of 7-(*p*-tolyl)-tryptophan in UPLC sample (µM)[c]	Concentration of 7-(*p*-tolyl)-tryptophan in culture (µM)[c]	Average Concentration of 7-(*p*-tolyl)-tryptophan (µM)[c]
**L4-Pd**_1	85588507	6.753	13.505	
**L4-Pd** _2	83664622	6.599	13.197	
**L4-Pd** _3	76625744	6.036	12.071	12.92 ± 0.75
**L3-Pd** _1	33033086	2.549	5.097	
**L3-Pd** _2	36082637	2.793	5.585	
**L3-Pd** _3	35302882	2.730	5.460	5.38 ± 0.25

Conditions: Cultures of *E. coli* RG-1500 grown in CCM containing *in vivo* generated 7-Br-tryptophan **2** (5 ml, 212 µM), **L3-Pd** or **L4-Pd** (50 µM), *p*-Tol-B(OH)$_2$ (1.0 mM) in phosphate buffer (45 ml) was incubated at 37 °C for 4 h. Reaction was quenched using DTT and cells were lysed. Clear supernatant collected after centrifugation was diluted with equal volume of methanol and analysed by UPLC (UPLC method 1). The product **2a** formed was quantified using the fluorescence peak area. No product was identified in control reactions without either Pd catalyst or *p*-Tol-B(OH)$_2$. Reactions were conducted in triplicate.

Supplementary Table 6. Streptomyces culturing for metabolite production.

***Streptomyces* strain**	**Antibiotic**	**Starter Media**	**Main Media**
S. coeruleorubidus (wild type)	None	ISP2	ISP2
S. coeruleorubidus/pSG19 (*prnA* expression plasmid) (RG5059)	Hygromycin (100 µg ml^{-1})	ISP2	MM
S. coelicolor (RG-4242)	Apramycin (50 µg ml^{-1})	2×YT	ISP2

Supplementary Table 7. Composition of the media, buffers and stock solutions used in this study.

Media/buffer	Composition
Lysogeny Broth (LB)	Tryptone (1% *w/v*), yeast extract (0.5% *w/v*), NaCl (17mM, 1% *w/v*)
2xYT	2x Yeast & Tryptone:<br>Tryptone (1.6% *w/v*), yeast extract (1% *w/v*), NaCl (0.5% *w/v*)
ISP2	International Streptomyces Production Media 2: Yeast extract (0.4% *w/v*), malt extract (1% *w/v*), D-glucose (0.4% *w/v*). pH to 7.2 using 1M NaOH
INA-5	1.5% (*w/v*), soytone, 0.5% (*w/v*), $CaCO_3$, 3% (*v/v*), glycerol, 0.2% (*w/v*), NaCl. pH to 7.3 using 1M NaOH
Pacidamycin Production Media (PPM)	Part A: Soytone (1% *w/v*), soluble starch (1% *w/v*).<br>Part B: D-maltose (2% *w/v*), trace element solution (0.05% *v/v*).<br>Each part autoclaved separately then combined in a 9:1 ratio of part A:B
LMR	KH_2PO_4 (100mM, 1.36% *w/v*), NaBr (100mM, 1.02% *w/v*), NaOH (25mM, 0.1% *w/v*), glucose (22mM, 0.4% *w/v*), H_2SO_4 (0.12mM), $(NH_4)_2SO_4$ (25mM, 0.33% *w/v*), $MgSO_4$ (2mM, 0.05% *w/v*), $FeSO_4$ (10μM), trace metal solution (0.02% *v/v*). pH to 7.8
SPG	$(NH_4)_2SO_4$ (25mM, 0.33% *w/v*), NaBr (100mM, 1% *w/v*), KH_2PO_4 (50mM, 0.68% *w/v*), Na_2HPO_4 (50mM, 0.71% *w/v*), $MgSO_4$ (2mM, 0.05% *w/v*), glucose (22mM, 0.4% *w/v*), trace metal solution (0.02% *v/v*). pH to 7.8
M9 Media	Na_2HPO_4 (50mM, 0.71% *w/v*), KH_2PO4 (22mM, 0.29% *w/v*), NaBr (100mM, 1.02% *w/v*), NH_4Cl (20mM, 0.11% *w/v*), glucose (22mM, 0.4% *w/v*), $MgSO_4$ (2mM, 0.05% *w/v*), $CaCl_2$ (0.1mM, 0.002% *w/v*), trace metal solution (0.02% *v/v*).pH to 7.8
M5	Soluble Starch (5.8mM, 2% *w/v*), $NaNO_3$ (1.18mM, 0.1% *w/v*), K_2HPO_4 (0.29mM, 0.05% *w/v*), $MgSO_4$ (0.42mM, 0.01% *w/v*), trace element solution (0.1% *v/v*)
Medium B	KH_2PO_4 (7.3mM, 0.1 % *w/v*), Glycine (33mM, 0.25 % *w/v*), Glycerol (274mM, 2% *v/v*), $MgSO_4$ (0.42mM, 0.01% *w/v*), $CaCO_3$ (1mM, 0.01% *w/v*), $FeSO_4$ (0.6 mM, 0.018% *w/v*), NaBr (180 mM), pH adjusted to 7.8
Medium B-A	Na_2HPO_4 (50mM, 0.7% *w/v*), KH_2PO_4 (22mM, 0.3 % *w/v*), Glycine (20mM, 0.15 % *w/v*), Glycerol (68mM, 0.5% *v/v*), $MgSO_4$ (0.42mM, 0.01% *w/v*), $CaCO_3$ (1mM, 0.01% *w/v*), NaBr (50 mM), pH to 8.5
Cross-coupling media (**CCM**)	Na_2HPO_4 (50mM, 0.7% *w/v*), KH_2PO_4 (22mM, 0.3 % *w/v*), KNO_3 (20mM, 0.2 % *w/v*), Glycerol (68mM, 0.5% *v/v*), $MgSO_4$ (0.42mM, 0.01% *w/v*), $CaCO_3$ (1mM, 0.01% *w/v*), NaBr (50 mM, 0.5% *w/v*), pH adjusted to 8.5
Soyflour mannitol (SFM) agar	D-mannitol (2% *w/v*), dissolved in tap water and heated at 50°C for 2 h before combination with soy flour (2% *w/v*, Rainbow Wholefoods), autoclaved twice with complete cooling between cycles.
ISP2-TSB Agar	50% ISP2 agar, 50% tryptic soy broth agar, melted and combined.
Trace element solution 1000x	H_3BO_4 (2.86% *w/v*), $MnCl_2$ (1.81% *w/v*), $ZnSO_4$ (0.22% *w/v*), Na_2MoO_4 (0.39% *w/v*), $CuSO_4$ (0.08% *w/v*), $Co(NO_3)_2$ (0.05% *w/v*), before use solution was diluted 1000 fold.

Supplementary Table 8. Key features of strains and plasmids used in this study.

**Strains**		
**Strain**	**Relevant genotype (purpose)**	**Source**
S. coeruleorubidus AB1183F-64	Wild-type (pacidamycin producer)	American Type Culture Collection
S. coelicolor M1154	Δ*act* Δ*red* Δ*cpk* Δ*cda* rpoB(C1298T) *rpsL* (A262G) (heterologous expression host)	John Innes Centre (Norwich, UK)
E. coli ET12567	F^- *dam*-13::Tn9 *dcm*-6 *hsdM hsdR ara14* (generates unmethylated DNA)	John Innes Centre (Norwich, UK)
E. coli BW25113	$lacI^q$ $rrnB_{T14}$ $lacZ_{WJ16}$ *hsdR514* $araBAD_{AH33}$ $rhaBAD_{LD78}$ (PCR-targeting host)	John Innes Centre (Norwich, UK)
S. coelicolor M1154 RG-4242	Integrated cosmid 2H-5Δ*neo*::*aac(3)IV-oriT-intΦ31-attPΦ31*-P2 (heterologous expression of pacidamycins)	This work
S. coeruleorubidus RG5059	Contains plasmid pSG19 (*prnA*) (tryptophan 7-halogenase expression strain)	Previous work[3]
S. coelicolor RG-1104	*S. coelicolor* RG-4242 containing pSG19 (*prnA*) (tryptophan 7-halogenase expression strain)	This work
**Plasmids**		
**Plasmid**	**Features (purpose)**	**Source**
pUZ8002	*neo tra* (conjugal transfer of cosmids)	John Innes Centre (Norwich, UK)
pIJ773	*oriT-acc(3)IV* (gene replacement cassette)	John Innes Centre (Norwich, UK)
pIJ790	λ-RED (*gam bet exo*) *cat araC rep101ts* (homologous recombination of DNA)	John Innes Centre (Norwich, UK)
Supercos I	*cos neo bla carb* (construction of cosmid libraries)	Stratagene (Agilent Technologies)
S. coeruleorubidus cosmid library (named by 96-well plate and position)	*cos neo bla carb* (heterologous expression of *S. coeruleorubidus* genes)	Our laboratory[31]
pIJ10702	Supercos I Δ*neo*::*aac(3)IV-oriT-intΦC31-attPΦC31* (heterologous expression)	John Innes Centre (Norwich, UK)
Supercos I-Int-2	Supercos I Δ*neo*::*aac(3)IV-oriT-intΦC31-attPΦC31*-P2 (control for heterologous expression)	This work
2H-5-Int-2	2H-5Δ*neo*::*aac(3)IV-oriT-intΦC31-attPΦC31*-P2 (heterologous expression of pacidamycin genes)	This work
pSG19	(*prnA* expression plasmid for actinomycetes)	Our laboratory[3]
pSG36	(*prnA: ssuE* expression plasmid for *E. coli*)	This work

Supplementary Table 9. Primers used in the construction of expression plasmid pSG36.

Name	Sequence 5'-3'
prnA-1	AGATTATCCATATGAACAAGCCGATCAAGAATATCGTCATCG (*Nde*I restriction site is underlined)
prnA-2	ATGCAAGCTTCTACAGGCTTTCCTGCGCTGCGAG (*Hin*dIII restriction site is underlined)
pPro-1	ATCTCGAGGGTACCGATGAGTCGACCTGCAGGCATG (*Xho*I and *Sal*I restriction sites are underlined)
pPro-2	ATCTCGAGCCATGGAATTCCTCCTGCTAGCCATGTTAG (*Xho*I and *Nco*I restriction sites are underlined)
ssuE-3	ATGCAAGCTTGCGGATAACAATTCCCCTCTAG (*Hin*dIII restriction site is underlined)
ssuE-4	ATGCAAGCTTTCACGCATGGGCATTACCTCGC (*Hin*dIII restriction site is underlined)
prnA-4	ATCCATGGGAAACAAGCCGATCAAGAATATCGTCATC (*Nco*I restriction site is underlined)
ssuE-5	ATGTCGACTCATCGGTACCCTCGACTCACGCATGGGCATTACCTCG (*Sal*I restriction site is underlined)

Supplementary Table 10. Accession number/sequence data of *prnA*, *ssuE* and pacidamycin cassette.

Name	NCBI Accession number	Sequence
prnA	AAB97504.1 (reference 4)	MNKPIKNIVIVGGGTAGWMAASYLVRALQQQAN ITLIESAAIPRIGVGEATIPSLQKVFFDFLGIPEREW MPQVNGAFKAAIKFVNWRKSPDPSRDDHFYHLF GNVPNCDGVPLTHYWLRKREQGFQQPMEYACYP QPGALDGKLAPCLSDGTRQMSHAWHFDAHLVA DFLKRWAVERGVNRVVDEVVDVRLNNRGYISNL LTKEGRTLEADLFIDCSGMRGLLINQALKEPFIDM SDYLLCDSAVASAVPNDDARDGVEPYTSSIAMNS GWTWKIPMLGRFGSGYVFSSHFTSRDQATADFLK LWGLSDNQPLNQIKFRVGRNKRAWVNNCVSIGL SSCFLEPLESTGIYFIYAALYQLVKHFPDTSFDPRL SDAFNAEIVHMFDDCRDFVQAHYFTTSRDDTPFW LANRHDLRLSDAIKEKVQRYKAGLPLTTTSFDDS TYYETFDYEFKNFWLNGNYYCIFAGLGMLPDRSL PLLQHRPESIEKAEAMFASIRREAERLRTSLPTNY DYLRSLRDGDAGLSRGQRGPKLAAQESL
ssuE (*ssuEADCB* set of genes)	AJ237695 (reference 5)	MRVITLAGSPRFPSRSSSLLEYAREKLNGLDVEVY HWNLQNFAPEDLLYARFDSPALKTFTEQLQQAD GLIVATPVYKAAYSGALKTLLDLLPERALQGKVV LPLATGGTVAHLLAVDYALKPVLSALKAQEILHG VFADDSQVIDYHHRPQFTPNLQTRLDTALETFWQ ALHRRDVQVPDLLSLRGNAHA
Pacidamycin gene cluster	GU938463 (GenBank)	

Supplementary Table 11: Structures of various known pacidamycin analogues isolated from wild type *Streptomyces coeruleorubidus* (AB1183F-64).[6]

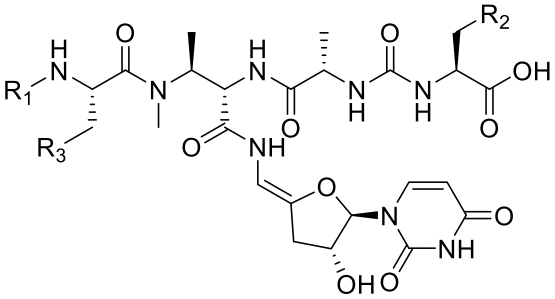

Pacidamycin	R[1]	R[2]	R[3]	MW[a]
Pacidamycin 1	Ala	Trp	*m*-Tyr	874.909
Pacidamycin 2	Ala	Phe	*m*-Tyr	835.872
Pacidamycin 3	Ala	*m*-Tyr	*m*-Tyr	851.871
Pacidamycin 4	H	Trp	*m*-Tyr	803.830
Pacidamycin 4N	CH_2	Trp	*m*-Tyr	815.841
Pacidamycin 5	H	Phe	*m*-Tyr	764.793
Pacidamycin 5T	H	*m*-Tyr	*m*-Tyr	780.792
Pacidamycin 6	Gly	Trp	*m*-Tyr	860.882
Pacidamycin 7	Gly	Phe	*m*-Tyr	821.845
Pacidamycin D	H	Trp	H	711.298
Pacidamycin S	H	Phe	H	672.287
Pacidamycin T	H	*m*-Tyr	H	688.282

[a]Calculated from structures generated in ChemBioDraw Ultra 14.0.

SUPPLEMENTARY DISCUSSION

Iterative development of media to sustain bacterial growth, halo-metabolite production and *in vivo* cross-coupling conditions.

In order to identify conditions suitable for cross-coupling of 7-Br-tryptophan **2** in living cultures, we set out to screen a wide range of media and/or it's components. Reciprocally, the potential toxicity of cross-coupling reagents was considered in selecting the appropriate concentration of aryl boronic acid and Pd catalysts. The reported MIC of phenyl boronic acid (Ph-B$(OH)_2$) against *E. coli* is 4 mM;[7] however 1 h exposure of *E. coli* cells to 0.5 mM **L2-Pd** catalyst was reported not to cause cell death.[8] Our *in vitro* model reaction comprised of utilizing synthetic 7-Br-tryptophan **2** (0.35 mM), *p*-Tol-B$(OH)_2$ (1.2 mM, 3.4 equiv.) and **L2-Pd** (100 µM) in various culture media or buffers. The final pH was adjusted to 7.5-8.5 using 100 mM K_2CO_3 if necessary. Product formation **2a** was monitored using either LCMS or UPLC analysis, results are summarized in Supplementary Table 2.

The initial screen revealed that cross-coupling conditions were tolerant to phosphate or citrate buffer (100 mM, pH 8), however, cross-coupling was completely inhibited by presence of ammonium chloride or ammonium sulfate (25 mM) in phosphate buffer. Similarly, poor conversions (~10%) were observed in the presence of glucose (22 mM, 0.4% *w/v*) or glycerol (217 mM, 2% *w/v*) which will both play a role in chelating to the boronic acid. No reaction or trace conversion (<2%) was observed using standard culture media (*e.g.* LB, 2×TY) or yeast/malt extracts. Moderate conversions (~30%) were observed in media-B (containing 180 mM NaCl/Br and 274 mM glycerol).

In the light of these results we iteratively screened and developed a number of modified media-B for compatibility with the cross-coupling as well as for the ability to promote the growth of *E. coli* RG-1500 and the cultures' production of Br-tryptophan. Using a modified media B-A (supplemented with 50 mM NaBr), a screen for the cross-coupling using 7-Br-tryptophan **2** (350 µM) and **L2-Pd** (100 µM) revealed an encouraging 75% conversion for the reactions with a 1:4 dilution in phosphate buffer, albeit at a high 5 mM concentration of boronic acid (Supplementary Table 3). Under identical conditions, reactions using media M5 worked remarkably well (80% conversion). A key feature of this starch rich media was the use of nitrate salt as a nitrogen source, in contrast to the glycine utilized in media-B or its variants. For full details of media composition, please refer to Supplementary Table 7.

Exploring the cross-coupling reaction in the spent culture media, and determining an efficient, cell compatible catalytic system.

We also considered the possibility that various soluble metabolites (*e.g.* proteins, amino acids, co-factors, bio-thiols, and free-radical species) that may be generated during the culturing of *E. coli* RG-1500, in parallel with the 7-Br-tryptophan **2**, could potentially affect the cross-coupling reaction. Hence, in order to determine the impact of any such components present within culture broth, we screened the cell free culture media (spent media, the supernatant remaining after centrifuging a mature culture of *E. coli* RG-1500 used for *in vivo* generation of 7-Br-Trytpophan **2**) for its potential inhibition of the cross-coupling reaction (Supplementary Table 4). Significantly higher conversions of cross-coupling product were achieved using spent cross-coupling media (CCM) (containing 0.28 mM 7-Br-tryptophan **2** generated *in vivo*) in comparison with spent media B-A.

Although the reactions with spent CCM worked well, the reaction time was very long (60 h) when *p*-Tol-B$(OH)_2$ (1.2 mM) and **L2-Pd** (100 µM) were used. In order to shorten the time, we then looked toward more active catalyst systems that might be suitable. Pd-catalysts based on [disodium-2-(dimethylamino)-pyrimidine-4,6-diol]$_2$-Pd$(OAc)_2$ (**L3-Pd**)[8] and (1,1,3,3-tetramethylguanidine)$_2$-Pd$(OAc)_2$ (**L4-Pd**)[9] were

recently reported to work well for the cross-coupling of activated aryl iodides. Importantly, both ligands are water-soluble and their lack of toxicity was attractive to us for *in vivo* applications. We were pleased to find both ligand variants (**L3-Pd** and **L4-Pd**) afforded increased catalytic activity with ~2 fold conversion over **L2-Pd** catalyst using the spent CCM. Amongst the two media, spent CCM again performed significantly better than media B-A for the cross-coupling reaction. Moreover, these conversions were obtained on 7-Br-tryptophan **2** substrate after 18 h using 100 µM Pd-catalyst and 1.2 mM *p*-Tol-B$(OH)_2$ (Supplementary Fig. 3). A time course experiment revealed a high UPLC yield of the cross-coupling product for both **L3-Pd** (76%) and **L4-Pd** (64%) within just 4 h using the spent CCM at 1:8 dilution (Supplementary Fig. 4). These conditions were considered suitable to screen cross-coupling in the presence of living cells.

SUPPLEMENTARY METHODS

General Information

All reagents were purchased from commercial suppliers and were used without further purification unless otherwise stated. Proton NMR (^1H), and carbon NMR (^{13}C) were recorded on either a Bruker Ascend 500 (500 MHz), Bruker 500 UltraShield (500 MHz), Bruker 400 UltraShield (400 MHz) or a Bruker UltraShield (300 MHz) spectrometer. The NMR experiments were carried out in deuterated chloroform ($CDCl_3$) deuterated water (D_2O), deuterated DMSO (d_6-DMSO) or deuterated methanol (d_4-MeOH). The chemical shifts (δ) are quoted in parts per million (ppm). Using a DEPTQ sequence or an HSQC experiment with multiplicity editing, the ^{13}C NMR signals were identified to CH_3, CH_2, CH and C. Coupling constants are reported in Hertz (Hz).

High and low resolution mass spectra that were recorded at the University of St Andrews were aquired on a either a Waters Micromass LCT time of flight mass spectrometer coupled to a Waters 2975 HPLC system or on an Orbitrap ELOS pro. Other spectra were acquired by the EPSRC National Mass Spectrometry Service, Swansea. Microwave reactions were effected in sealed vials using a Biotage Initiator+ microwave reactor. UPLC analysis was performed on a Waters Acquity H-Class UPLC system fitted with a Waters Acquity UPLC BEH C18 column (1.7 µm, 2.1×50 mm) or Phenomenex Kinetex Phenyl-hexyl column (2.1 µm, 2.1×75 mm).

Flash chromatography was performed using Davisil silica gel LC60A (40-63 micron). Thin layer chromatography (TLC) was executed using aluminium sheets of silica gel 60 F254 and was visualised under a Mineralight model UVGL-58 lamp (254 nm). The plates were developed with ninhydrin in acetone or basic potassium permanganate solutions. Purification of unprotected tryptophan derivatives and peptides was carried out on a Biotage Isolera Four using reverse-phase SNAP C18 12 g column cartridges. The purification was carried out using water (solvent A) and methanol/acetonitrile (solvent B) using the following gradient: 0-1.5 min (5% B), 1.5-3.0 min (5% to 15% B), 3.0-5.0 min (15% B), 5.0-15.0 min (15% to 95% B), 15.0-18.0 min (95% B), 18.0-20.0 min (95%-5% B), 20.0-25.0 min (5% B) at a flow rate of 12-15 ml min^{-1}.

5-Br-, 7-Cl- and 7-Br-tryptophan (**13**, **12** and **2**)[10] and tripeptides containing 7-Br-tryptophan (H-Ala-Trp-(7-Br)-Phe-OH **14**, H-Trp-(7-Br)-Ala-Phe-OH **15**) were prepared as described previously.[11] A 10 mM stock solution of palladium catalyst using **L2**, **L3** and **L4** ligand was prepared as reported previously.[8,9,12]

Experiemental and characterisation data of synthetic products.

Cross-coupling of halo-indoles using L1-Pd catalyst.

In a screw cap glass vial, appropriate halo-indole (0.1 mmol), *p*-Tol-B(OH)$_2$ (20 mg, 0.15 mmol), potassium carbonate (42 mg, 0.3 mmol) were suspended in water-CH_3CN mixture (4:1, 1.8 ml). A solution of **L1-Pd** prepared using Na_2PdCl_4 (5 mol%, 1.4 mg) and ^{S}SPhos (12 mol%, 6 mg) in water (0.2 ml) was added. The vial was closed and stirred at 37 °C until completion. The reaction was diluted with brine (2 ml) and extracted with ethyl acetate (3 × 3-4 ml). The combined organic extract was dried ($MgSO_4$), filtered and solvent removed under reduced pressure. The desired product was purified by flash column chromatography using hexane-DCM solvent system (10-90% gradient).

5-(*p*-Tolyl)-1H-indole (5a)[13]

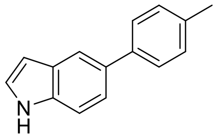

The above procedure afforded 19 mg (92% from 5-Br-indole **5**) of the desired product **5a** as a white solid. **^{1}H NMR (300 MHz, CDCl$_3$)** δ 8.12 (bs, 1H), 7.95 – 7.88 (m, 1H), 7.63 (d, *J* = 8.1 Hz, 2H), 7.53 – 7.42 (m, 2H), 7.32 (d, *J* = 8.1 Hz, 2H), 7.27 – 7.22 (m, 1H), 6.70 – 6.62 (m, 1H), 2.47 (s, 3H) ppm; **^{13}C NMR (75 MHz, CDCl$_3$)** δ 139.78, 136.07, 135.26, 133.46, 129.50, 128.47, 127.35, 124.91, 121.93, 119.09, 111.30, 103.05, 21.19 ppm; **MS (ESI)** *m/z* 208.09 $[M+H]^+$; **HRMS (FTMS +p ESI)** *m/z* $C_{15}H_{14}N$ $[M+H]^+$ calculated 208.1121, found 208.1120.

7-(*p*-Tolyl)-1H-indole (7a)[14]

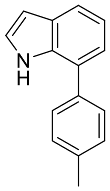

The above procedure afforded 20 mg (96% from 7-Br-indole **7**) of the desired product **7a** as an off-white solid. **^{1}H NMR (400 MHz, CDCl$_3$)** δ 8.44 (bs, 1H), 7.66 (ddd, *J* = 5.9, 3.2, 0.7 Hz, 1H), 7.57 (dt, *J* = 8.1, 1.8 Hz, 2H), 7.36 (d, *J* = 8.1 Hz, 2H), 7.26 – 7.21 (m, 3H), 6.65 (dd, *J* = 3.2, 2.1 Hz, 1H), 2.47 (s, 3H) ppm; **^{13}C NMR (100 MHz, CDCl$_3$)** δ 137.3, 136.4, 133.9, 129.9, 128.3, 128.2, 125.7, 124.4, 121.9, 120.4, 119.9, 103.2, 21.4 ppm; **MS (ESI)** *m/z* 208.25 $[M+H]^+$; **HRMS (FTMS +p ESI)** *m/z* $C_{15}H_{14}N$ $[M+H]^+$ calculated 208.1121, found 208.1121.

Preparative scale cross-coupling of unprotected halo-tryptophan using L2-Pd catalyst.

In a screw cap glass vial, appropriate 5- or 7-Br-tryptophan (**13** or **2**, 14 mg, 0.05 mmol), *p*-Tol-B(OH)$_2$ (20 mg, 0.15 mmol), potassium carbonate (41 mg, 0.3 mmol) were suspended in water-EtOH mixture (4:1, 1.0 ml). A solution of **L2-Pd** (5 mol%) in water (0.2 ml) was added. The vial was closed and stirred at 45 °C for 48 h. The reaction was diluted with water (2 ml) and extracted with diethyl ether (3 × 2 ml). The aqueous layer was acidified (pH ~2-3) using 0.1 M HCl. Solvent was removed under reduced pressure. The desired product was obtained by purification using gradient reversed phase chromatography (C-18, 12 g) eluting with water-MeOH (5-95% gradient).

7-(*p*-Tolyl)-tryptophan (2a)

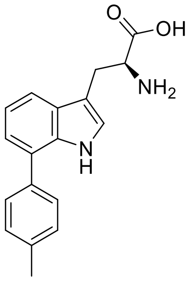

The above procedure procedure afforded 12.5 mg (85% yield) of the desired product **2a** as a white solid. **^{1}H NMR (400 MHz, CD_3OD)** δ 7.67 – 7.59 (m, 1H), 7.52 (d, *J* = 8.1 Hz, 2H), 7.34 (d, *J* = 7.9 Hz, 2H), 7.26 (s, 1H), 7.21 – 7.14 (m, 2H), 4.24 (dd, *J* = 8.2, 4.7 Hz, 1H), 3.56 (dd, *J* = 15.1, 4.8 Hz, 1H), 3.42 – 3.34 (m, 1H), 2.43 (s, 3H) ppm; **^{13}C NMR (100 MHz, CD_3OD)** δ 172.17, 138.15, 137.62, 135.73, 130.58, 129.22, 129.08, 127.45, 126.11, 122.93, 120.92, 118.16, 108.58, 54.92, 27.80, 21.25 ppm; **MS (ESI)** *m/z* 295.17 $[M+H]^+$; **HRMS (FTMS +p ESI)** *m/z* $C_{18}H_{19}N_2O_2$ $[M+H]^+$ calculated 295.1441, found 295.1436.

5-(*p*-Tolyl)-tryptophan (13a)[15]

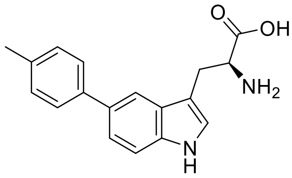

The above procedure afforded 8 mg (54% yield) of the desired product **13a** as a white solid. **^{1}H NMR (500 MHz, CD_3OD)** δ 7.99 (t, *J* = 1.1 Hz, 1H), 7.60 (d, *J* = 8.1 Hz, 2H), 7.46 - 7.41 (m, 2H), 7.26 (d, *J* = 7.9 Hz, 2H), 7.24 (s, 1H), 3.91 (dd, *J* = 9.6, 4.0 Hz, 1H), 3.58 (dd, *J* = 15.2, 3.5 Hz, 1H), 3.19 (dd, *J* = 15.0, 9.7 Hz, 1H), 2.39 (s, 3H) ppm; ^{13}C NMR **(125 MHz, CD_3OD)** δ 174.58, 141.06, 137.81, 136.82, 133.84, 130.26, 129.01, 128.04, 125.85, 122.30, 117.50, 112.67, 110.08, 56.75, 28.64, 21.08 ppm; **MS (ESI)** *m/z* 295.21 $[M+H]^+$, 589.31 $[2M+H]^+$; **HRMS (FTMS +p ESI)** *m/z* $C_{18}H_{19}N_2O_2$ $[M+H]^+$ calculated 295.1441, found 295.1437.

H-Ala-Trp-(7-Br)-Phe-OH (14)[11]

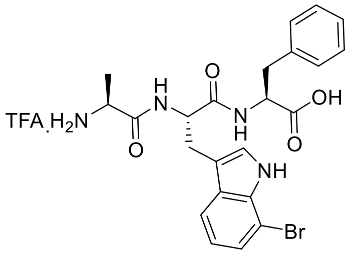

This compound was synthesised as reported previously.[11] **^{1}H NMR (400 MHz, CD_3OD)** δ 7.64 (dd, *J* = 7.9, 0.6 Hz, 1H), 7.29 – 7.25 (dd, *J* = 9.5, 7.4 Hz, 2H), 7.24 – 7.14 (m, 5H), 6.96 (t, *J* = 7.8 Hz, 1H), 4.75 (dd, *J* = 8.5, 5.7 Hz, 1H), 4.63 (dd, *J* = 8.0, 5.2 Hz, 1H), 3.84 (q, *J* = 7.0 Hz, 1H), 3.27 (dd, *J* = 14.8, 5.6 Hz, 1H), 3.19 (dd, *J* = 13.9, 5.2 Hz, 1H), 3.08 (dd, *J* = 14.8, 8.6 Hz, 1H), 3.01 (dd, *J* = 13.9, 8.1 Hz, 1H), 1.42 (d, *J* = 7.1 Hz, 3H) ppm; **^{13}C NMR (100 MHz, CD_3OD)** δ 174.32, 173.10, 170.75, 138.22, 136.41, 130.38, 130.25, 129.39, 127.74, 125.93, 125.02, 121.08, 118.84, 112.15, 105.60, 55.37, 55.22, 50.05, 38.33, 29.11, 17.70 ppm; **MS (ESI)** *m/z* 501.8 $[M+H]^+$; **HRMS (FTMS +p ESI)** *m/z* $C_{23}H_{26}BrN_4O_4$ $[M+H]^+$ calculated 501.1132, found 501.1128.

H-Trp-(7-Br)-Ala-Phe-OH (15)[11]

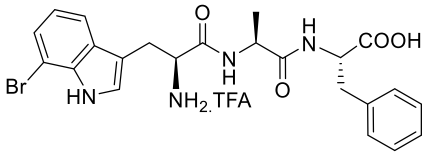

This compound was synthesised as reported previously.[11] **^{1}H NMR (400 MHz, CD$_3$OD)** δ 7.67 (d, *J* = 7.9 Hz, 1H), 7.34 – 7.25 (m, 6H), 7.20 (dq, *J* = 8.7, 4.1 Hz, 1H), 6.95 (t, *J* = 7.8 Hz, 1H), 4.67 (dd, *J* = 8.2, 5.3 Hz, 1H), 4.44 (q, *J* = 7.1 Hz, 1H), 4.15 (dd, *J* = 8.7, 5.4 Hz, 1H), 3.38 (dd, *J* = 15.1, 5.3 Hz, 1H), 3.22 (dd, *J* = 14.0, 5.3 Hz, 1H), 3.15 (dd, *J* = 15.1, 8.9 Hz, 1H), 3.04 (dd, *J* = 14.0, 8.2 Hz, 1H), 1.38 (d, *J* = 7.1 Hz, 3H) ppm; **^{13}C NMR (125 MHz, CD$_3$OD)** δ 173.22, 172.68, 168.18, 136.95, 135.35, 129.06, 128.47, 128.04, 126.40, 125.52, 124.11, 120.13, 117.23, 107.99, 104.43, 53.90, 53.23, 49.06, 36.92, 27.51, 16.91 ppm; **MS (ESI)** *m/z* 501.3 [M+H]$^+$; **HRMS (FTMS +p ESI)** *m/z* $C_{23}H_{26}BrN_4O_4$ [M+H]$^+$ calculated 501.1132, found 501.1132.

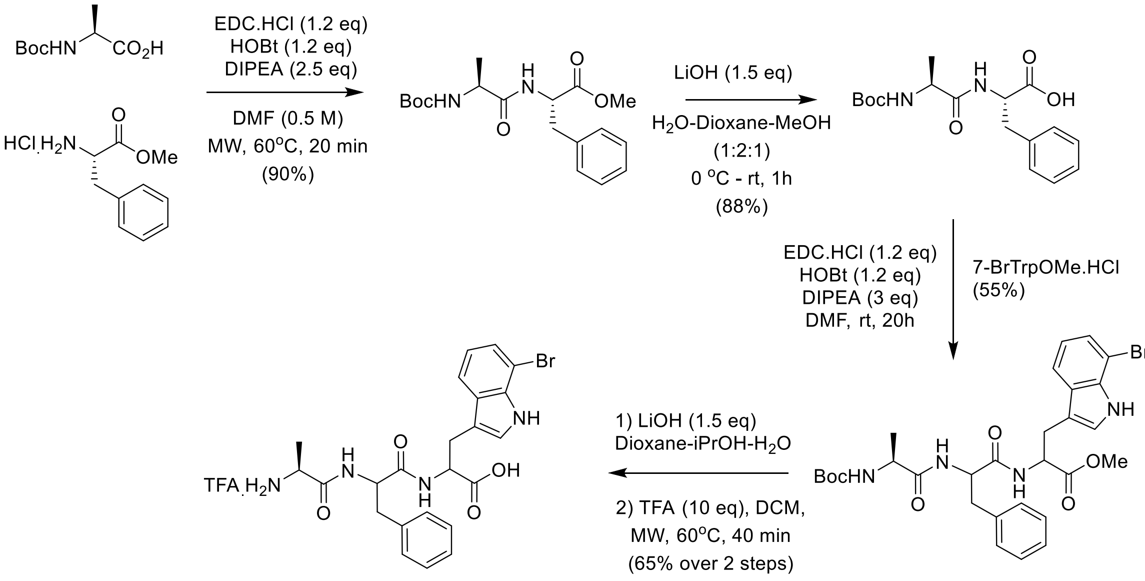

Supplementary Scheme 1. Synthesis of H-Ala-Phe-Trp-(7-Br)-OH (16)

N-Boc-Ala-Phe-OMe[16]

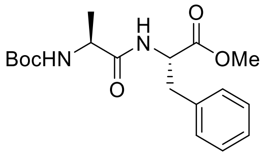

In a microwave vial, to a solution of *N*-Boc-Ala-OH (440 mg, 2.32 mmol) in DMF (4.6 ml), EDC.HCl (536 mg, 2.79 mmol) and HOBt (375mg, 2.79 mmol) were added. The mixture was stirred for 5 min, DIPEA (1 ml, 5.8 mmol) was added and stirred for 10 min. Solid H-Phe-OMe.HCl (500 mg, 2.33 mmol) was added in one portion and reaction vial was sealed with an aluminium crimp cap. Reaction was heated in a microwave reactor at 60 °C for 20 min. After cooling to ambient temperature, the reaction was diluted with EtOAc (25 ml) and washed successively with water (3 × 10 ml), dilute sodium bicarbonate (10 ml) and brine (10 ml). The organic extract was dried ($MgSO_4$), filtered and the solvent evaporated under reduced pressure on a rotary evaporator. The product was obtained as white waxy solid (750 mg, 90% yield) and was used without further purification. **^{1}H NMR (400 MHz, CDCl$_3$)** δ 7.37 – 7.16 (m, 3H), 7.15 – 7.03 (m, 2H), 6.83 (bs, 1H), 5.27 (bs, 1H), 4.83 (q, *J* = 6.2 Hz, 1H), 4.18 (bs, 1H), 3.67 (s, 3H), 3.13 (dd, *J* = 13.8, 5.9 Hz, 1H), 3.05 (dd, *J* = 13.8, 6.3 Hz, 1H), 1.42 (s, 9H), 1.28 (d, *J* = 7.0 Hz, 3H)

ppm; **^{13}C NMR (100 MHz, $CDCl_3$)** δ 172.51, 171.78, 155.35, 135.87, 129.26, 128.50, 127.04, 79.90, 53.25, 52.26, 50.03, 37.87, 28.29, 18.35 ppm; **MS (ESI)** *m/z* $C_{18}H_{26}N_2O_5Na$ $[M+Na]^+$ calculated 373.17, found 373.21.

***N*-Boc-Ala-Phe-OH**

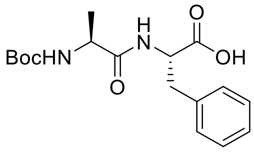

N-Boc-Ala-Phe-OMe (300 mg, 0.86 mmol) was dissolved in a mixture of 1,4-dioxane-MeOH-water (4:2:1, 7 ml) and cooled in an ice-bath. A solution of LiOH (1.3 mmol, 1.5 equiv. in 1 ml water) was added dropwise. The mixture was stirred vigorously until all the starting material has been consumed (TLC, hexanes-EtOAc 1:1). Subsequently, the reaction was diluted with water (5 ml) and neutralised (pH ~ 6-7) using aqueous 1M HCl. The organic solvents were removed under reduced pressure, followed by acidifying the solution (pH ~1-2, using 1M HCl). The qqueous layer was extracted with EtOAc (3 × 10 ml). The organic extract was dried ($MgSO_4$), filtered and solvent evaporated under reduced pressure, affording the dipeptide acid (*N*-Boc-Ala-Phe-OH, 260 mg, 88% yield), which was used without further purification. **^{1}H NMR (400 MHz, CD_3OD)** δ 7.36 – 7.13 (m, 5H), 4.52 (t, *J* = 5.7 Hz, 1H), 4.04 (d, *J* = 6.7 Hz, 1H), 3.22 (dd, *J* = 13.6, 5.3 Hz, 1H), 3.05 (dd, *J* = 13.7, 6.6 Hz, 1H), 1.44 (s, 9H), 1.26 (d, *J* = 7.2 Hz, 3H) ppm; **MS (ESI)** *m/z* 359.17 $[M+Na]^+$, 695.35 $[2M+Na]^+$; **HRMS (FTMS +p ESI)** *m/z* $C_{17}H_{24}N_2O_5Na$ $[M+Na]^+$ calculated 359.1577, found 359.1573.

7-Br-tryptophan methyl ester hydrochloride[11]

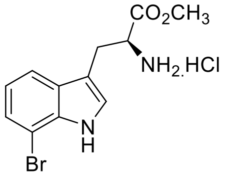

At 0 °C, in a dry flask, thionyl chloride (290 µl, 4 mmol) was added to dry methanol (10 ml) under inert atmosphere. After 10 min, 7-Br-tryptophan **2** (285 mg, 1 mmol) was added at 0 °C. The reaction was stirred overnight while allowing it to warm to room temperature. Evaporation of the solvents and drying under high vacuum afforded the title compound as an off white hydrochloride salt (330 mg, quantitative). This compound was used without further purification.

NMR on HCl salt (DMSO-d_6) revealed broad signals. Hence, an analytical sample was obtained by desalting with dilute $NaHCO_3$ and extraction with EtOAc. Drying ($MgSO_4$) and evaporation of the solvent afforded the free base suitable for NMR in $CDCl_3$. **^{1}H NMR (400 MHz, $CDCl_3$)** δ 8.37 (s, 1H), 7.58 (d, *J* = 7.9 Hz, 1H), 7.37 (dd, *J* = 7.7, 0.7 Hz, 1H), 7.15 (d, *J* = 2.2 Hz, 1H), 7.03 (t, *J* = 7.8 Hz, 1H), 3.85 (dd, *J* = 7.4, 5.0 Hz, 1H), 3.73 (s, 3H), 3.27 (ddd, *J* = 14.4, 5.0, 0.7 Hz, 1H), 3.08 (dd, *J* = 14.4, 7.4 Hz, 1H) ppm; **^{13}C NMR (100 MHz, $CDCl_3$)** δ 175.78, 135.08, 128.85, 124.66, 123.62, 120.87, 118.22, 112.73, 104.99, 55.11, 52.20, 30.94 ppm; **MS (ESI)** *m/z* 297.02 $[M+H]^+$; **HRMS (FTMS +p ESI)** *m/z* $C_{12}H_{14}BrN_2O_2$ $[M+H]^+$ calculated 297.0233, found 297.0228.

N-Boc-Ala-Phe-Trp-(7-Br)-OMe

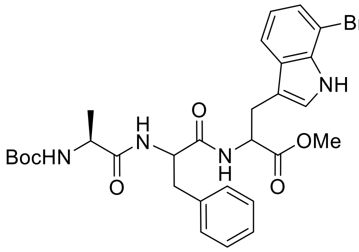

To a solution of *N*-Boc-Ala-Phe-OH (130 mg, 0.38 mmol) in DMF (2 ml), EDC.HCl (82 mg, 0.44 mmol) and HOBt (58 mg, 0.43 mmol) were added. The mixture was stirred for 5 min, DIPEA (0.21 ml, 1.2 mmol) was added dropwise. Reaction mixture was stirred at ambient temperature for 10 min and solid H-Trp-(7-Br)-OMe.HCl (130 mg, 0.39 mmol) was added in one portion. Reaction was stirred at ambient temperature for 20 h. DMF was evaporated under reduced pressure. The residue was suspended in EtOAc (20 ml) and washed successively with water (3 × 10 ml) and brine (10 ml). The organic extract was dried ($MgSO_4$), filtered and solvent evaporated under reduced pressure. Final protected tripeptide was obtained as yellow waxy solid (130 mg, 55% yield) after flash column chromatography (SiO_2, hexanes- EtOAc, 10-90% gradient). This product was obtained as an inseparable diastereomeric mixture due to epimerisation during peptide coupling reaction. **^{1}H NMR (300 MHz, CD_3OD)** δ 7.50 (dd, *J* = 12.5, 8.0 Hz, 1H), 7.32 – 7.06 (m, 6H), 7.00 – 6.91 (m, 2H), 4.75 – 4.55 (m, 2H), 3.97 (d, *J* = 7.2 Hz, 1H), 3.67 & 3.62 (2 × s, 3H), 3.30 – 3.02 (m, 3H), 2.98 – 2.64 (m, 1H), 1.40 (s, 9H), 1.15 & 1.06 (2 × d, *J* = 7.2 Hz, 3H) ppm; **^{13}C NMR (75 MHz, CD_3OD)** δ 174.30, 174.06, 171.65, 156.24, 136.61, 134.89, 129.05, 128.74, 127.98, 126.33, 124.42, 123.61, 119.76, 117.28, 110.44, 104.24, 79.22, 54.00, 53.25, 51.40, 50.32, 37.48, 27.36, 27.14, 16.86 ppm (major disastereomeric peaks are reported); **MS (ESI)** *m/z* 615.18 $[M+H]^+$, 637.18 $[M+Na]^+$; **HRMS (FTMS +p ESI)** *m/z* $C_{29}H_{35}BrN_4O_6Na$ $[M+Na]^+$ calculated 637.1632, found 637.1614.

H-Ala-Phe-Trp-(7-Br)-OH (16)

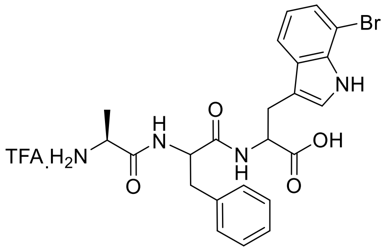

N-Boc-Ala-Phe-Trp-(7-Br)-OMe (124 mg, 0.21 mmol) was dissolved in a mixture of 1,4-dioxane-^{i}PrOH-water (1:1:0.5, 4 ml) and cooled in an ice-bath. A solution of LiOH (1.5 equiv. in 1 ml) was added dropwise. The mixture was stirred vigorously until all the starting material has been consumed (TLC, Hexane-EtOAc 1:1). Subsequently, the reaction was diluted with water (2 ml) and quenched by addition of Amberlyst-120-H^+ (to pH ~5-6). Resin was filtered and the solvent was evaporated under reduced pressure to provide the crude free acid (*N*-Boc-Ala-Phe-Trp-(7-Br)-OH). This intermediate was dissolved in CH_2Cl_2 (3 ml) and transferred to a microwave vial. TFA (65 µl, 2 mmol) was added and the reaction vial was sealed with an aluminium crimp cap. Reaction mixture was heated in a microwave reactor at 60 °C for 40 min. After cooling, the solvent was evaporated under reduced pressure. The residue was purified using gradient reversed phase chromatography (C-18, 12 g, water-MeOH, 5-95% gradient) affording a diastereomeric mixture of H-Ala-Phe-Trp-(7-Br)-OH **16** as a TFA salt (84 mg, 65% over two steps). **^{1}H NMR (400 MHz, CD_3OD)** δ 7.63 – 7.55 (m, 1H), 7.28 (t, *J* = 7.0 Hz, 1H), 7.26 – 7.21 (m, 3H), 7.21 – 7.17 (m, 1H), 7.16 – 7.11 (m, 1H), 7.04 – 6.99 (m, 1H), 7.00 – 6.92 (m, 1H), 4.82 – 4.70 (m, 1H), 4.67 (dd, *J* = 9.2, 5.4 Hz, 1.3 H), 3.85 (q, *J* = 6.3, 5.6 Hz, 0.7 H), 3.43 – 3.35 (m, 1H), 3.27 – 3.06 (m, 1.7 H), 3.00 – 2.80 (m, 1H), 2.64 (dd, *J* = 13.9, 9.5 Hz, 0.3 H), 1.39 (d, *J* = 7.1 Hz, 2H), 1.18 (d, *J* = 7.0 Hz, 1H) ppm; **^{13}C NMR (75 MHz, CD_3OD)** δ 173.17, 171.62, 169.51, 136.73, 134.91, 129.02,

128.80, 128.06, 126.42, 124.51, 123.57, 119.74, 117.44, 110.74, 104.15, 54.91, 53.11, 48.61, 37.36, 27.12, 16.25 ppm (major disastereomeric peaks are reported); **MS (ESI)** *m/z* 501.1 $[M+H]^+$; **HRMS (FTMS +p ESI)** *m/z* $C_{23}H_{26}BrN_4O_4$ $[M+H]^+$ calculated 501.1132, found 501.1123.

Cross-coupling of unprotected tripeptides (14-16) containing 7-Br-tryptophan using L2-Pd catalyst.

In a screw cap glass vial, appropriate tripeptide (10 mg, 0.02 mmol), *p*-tol-B(OH)$_2$ (8.2 mg, 0.06 mmol), potassium carbonate (16 mg, 0.12 mmol) were suspended in water (1.0 ml). A solution of **L2-Pd** (5 mol%) in water (0.04 ml, 10 mM stock) was added. The vial was closed and stirred at 45 °C for 48 h. The reaction was diluted with water (2 ml) and extracted with diethyl ether (3 × 2 ml). Aqueous layer was acidified (pH ~2) using 0.1 M HCl. The solvent was removed under reduced pressure. The desired product was obtained by purification using gradient reversed phase chromatography (C-18, 12 g) eluting with water-MeOH (5-95% gradient) affording **14a-16a**.

H-Ala-Trp-(7-*p*-tol)-Phe-OH (14a)

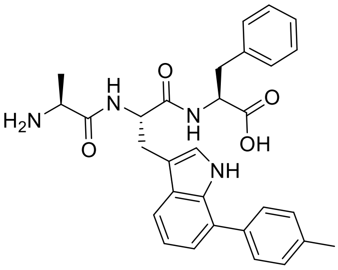

The above procedure afforded 6.8 mg (66% yield) of the desired product **14a** as a white solid. **^{1}H NMR (500 MHz, CD$_3$OD)** δ 7.62 (dd, *J* = 6.0, 3.0 Hz, 1H), 7.49 (d, *J* = 8.1 Hz, 2H), 7.30 (d, *J* = 7.8 Hz, 2H), 7.20 – 7.17 (m, 1H), 7.17 – 7.13 (m, 4H), 7.11 (m, 3H), 4.69 (dd, *J* = 9.3, 4.8 Hz, 1H), 4.44 (t, *J* = 5.6 Hz, 1H), 3.72 (q, *J* = 7.0 Hz, 1H), 3.38 – 3.35 (m, 1H), 3.21 (dd, *J* = 13.6, 5.2 Hz, 1H), 3.08 (dd, *J* = 14.9, 9.4 Hz, 1H), 3.06 (dd, *J* = 13.5, 5.9 Hz, 1H), 2.41 (s, 3H), 1.39 (d, *J* = 7.1 Hz, 3H) ppm; **^{13}C NMR (125 MHz, CD$_3$OD)** δ 175.67, 171.13, 170.08, 137.72, 136.57, 136.42, 134.02, 129.45, 129.12, 127.99, 127.81, 127.65, 125.87, 125.71, 123.84, 121.12, 119.10, 117.12, 110.10, 55.69, 54.77, 48.86, 37.45, 27.52, 19.83, 16.58 ppm; **MS (ESI)** *m/z* 513.3 $[M+H]^+$; **LC-HRMS (FTMS +p ESI)** *m/z* $C_{30}H_{33}N_4O_4$ $[M+H]^+$ calculated 513.2496, found 513.2489. Only one peak was observed on HPLC (LCMS) indicating the absence of epimerisation during cross-coupling (retention time 5.66 min.)

H-Trp-(7-*p*-tol)-Ala-Phe-OH (15a)

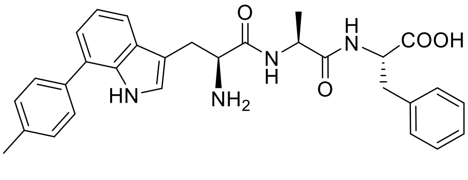

The above procedure afforded 6.4 mg (62% yield) of the desired product **15a** as a white solid. **^{1}H NMR (500 MHz, CD$_3$OD)** δ 7.66 (dd, *J* = 6.7, 2.2 Hz, 1H), 7.52 (d, *J* = 8.0 Hz, 2H), 7.34 (d, *J* = 7.9 Hz, 2H), 7.29 – 7.23 (m, 4H), 7.23 – 7.11 (m, 4H), 4.64 (dd, *J* = 8.1, 5.5 Hz, 1H), 4.44 (q, *J* = 7.0 Hz, 1H), 4.17 (dd, *J* = 8.6, 5.7 Hz, 1H), 3.41 (dd, *J* = 15.0, 5.6 Hz, 1H), 3.24 – 3.12 (m, 2H), 3.02 (dd, *J* = 13.9, 8.2 Hz, 1H), 2.44 (s, 3H), 1.39 (d, *J* = 7.1 Hz, 3H) ppm; **^{13}C NMR (125 MHz, CD$_3$OD)** δ 174.36, 174.10, 169.68, 138.23, 138.18, 137.62, 135.70, 130.60, 130.42, 129.47, 129.26, 129.02, 127.84, 127.46, 126.29, 122.95, 120.93, 118.19, 108.40, 55.14, 54.79, 50.39, 38.27, 28.96, 21.27, 18.39 ppm; **MS (ESI)** *m/z* 513.5

$[M+H]^+$; **LC-HRMS (FTMS +p ESI)** *m/z* $C_{30}H_{33}N_4O_4$ $[M+H]^+$ calculated 513.2496, found 513.2487. Only one peak was observed on HPLC (LCMS) indicating the absence of epimerisation during cross-coupling (retention time 5.70 min).

H-Ala-Phe-Trp-(7-*p*-tol)-OH (16a)

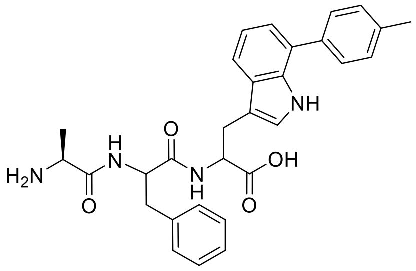

The general procedure afforded 7.3 mg (71% yield) of the desired product **16a** as a white solid. **^{1}H NMR (500 MHz, CD_3OD)** δ 7.65 – 7.57 (m, 1H), 7.50 & 7.48 (2 × d, *J* = 8.0 Hz, 2H), 7.31 (d, *J* = 8.2 Hz, 2H), 7.27 – 7.20 (m, 2H), 7.21 – 7.16 (m, 2H), 7.15 – 7.13 (m, 2H), 7.12 – 7.06 (m, 2H), 4.67 – 4.62 (m, 2H), 4.57 – 4.49 (m, 1H), 3.75 – 3.61 (m, 1H), 3.56 – 3.39 (m, 1H), 3.26 – 3.17 (m, 1H), 2.73 – 3.71 (m, 1H), 2.42 & 2.41 (2 × s, 3H), 1.32 & 1.10 (2 × d, *J* = 7.0 Hz, 3H) ppm (disastereomeric mixture); **^{13}C NMR (125 MHz, CD_3OD)** δ 177.03, 176.39, 170.89, 137.35, 136.63, 136.43, 133.64, 129.10, 128.94, 128.68, 128.06, 127.78, 126.30, 125.35, 123.82, 120.71, 118.78, 117.71, 110.69, 55.69, 55.51, 54.02, 37.04, 27.12, 19.84, 16.72 ppm (major diastereomeric peaks are reported); **MS (ESI)** *m/z* 513.2 $[M+H]^+$; **LC-HRMS (FTMS +p ESI)** *m/z* $C_{30}H_{33}N_4O_4$ $[M+H]^+$ calculated 513.2496, found 513.2488; Two peaks were observed on HPLC (LCMS) indicating the presence of diastereomeric mixture (retention times: 5.64 and 5.93 min) which arise from the diastereomeric mixture of substrate **16**.

Enantiopurity analysis of tryptophan derivatives using Marfey's reagent.

The enantiopurity of the cross-coupled 7-(*p*-tolyl)-tryptophan was analysed using Marfey's Reagent (1-fluoro-2,4-dinitrophenyl-5-L-alanine amide, FDAA).[17] A stock solution of 10.0 mM FDAA in acetone was prepared by dissolving 2.7 mg FDAA in 1 ml acetone. The sample was dissolved in 1 M sodium hydrogen carbonate (0.1-1 mg ml^{-1}). FDAA stock solution were (50 µl) added to 100 µl of sample solution, and the mixture incubated at 40 °C for 1h. The reaction was quenched with 100 µl of 1 M hydrochloric acid. The reaction mixture was diluted (10 µl in 190 µl water) and centrifuged (13,000 rpm, 5 min). Clear supernatant was analysed by UPLC with UV detection at 340 nm. A mixture of L-tryptophan to D-tryptophan was used as a standard to confirm the separation of enantimers. The FDAA derivative of 7-(*p*-tolyl)-tryptophan revealed only peak, indicating presence of single enantiomer (Supplementary Fig. 5). UPLC analysis was performed on Waters Acquity UPLC BEH C18 (1.7µm 2.1× 50 mm) column eluting with 0.1% TFA in water (solvent A) and acetonitrile (solvent B). Following gradient was used: 0-0.2 min (20% B), 0.2-4.0 min (20% to 70% B), 4.0-4.2 (70% to 90% B), 4.2-5.2 min (90% B), 5.2-5.5 (90% to 20%B), 5.5-6.0 (20% B). The flow rate was set to 600 µl min^{-1} and the column temperature was maintained at 50°C. Detection was by UV (PDA 200-400nm, UV 340 nm).

General biological procedures.

All chemical and biological components and materials used for media, buffers and stock solutions were purchased from commercial sources and used as received. Materials were stored according to supplier's guidelines. For long-term storage of microorganisms, cell stocks were stored at -80 °C in 20% (*v/v*) aqueous glycerol. Frequently used microorganisms were stored at -20 °C under the same conditions. *Streptomyces* species were stored at -20 °C as spore suspensions prepared from 10-14 day old SFM agar plates containing appropriate antibiotics. Spore suspensions were prepared by gently washing the aerial mycelium (containing spores) with sterile water, collection of spores by centrifugation (30 min, 4000 × g at 4 °C) and resuspension in sterile 20% glycerol (1 ml) before storage at -20 °C. Heat sterilisation of water, media and culturing equipment was carried out using a Boxer Benchtop Denley autoclave (121 °C for 20 min at 1.3 bar) or disposable pre-sterilised apparatus was used. Filter sterilisation of all other heat labile media, buffer and stock solutions was performed by passing through a 0.2 μm membrane. 18 MΩ water was obtained from water purified in-house using an Elga PURELAB flex 2 polisher. Microorganisms were cultured under sterile conditions using either a lit bench top Bunsen or within a Faster BH-EN class II vertical laminar airflow cabinet. DNA manipulation, microbial culturing, and natural product extraction were carried out following standard protocols unless otherwise stated.[18-21] Primers for cloning were purchased from Invitrogen. For selective cultures, kanamycin was used at 50 μg ml^{-1}, ampicillin at 100 μg ml^{-1}, nalidixic acid at 25 μg ml^{-1}, apramycin at 50 μg ml^{-1} and chloramphenicol at 25 μg ml^{-1}.

Microbial cultures were incubated using a New Brunswick Scientific (Innova 4300 or I26) incubator shaker, or a static Genlab incubator. Freeze-drying was carried out using a SCAnVAC cool safe freeze dryer with a vacuubrand hybrid pump RC6. Cell lysis for the release of proteins was performed in lysis buffer using either sonication with a Phillip Harris Scientific Status US200 ultra-sonicator fitted with a Bandelin Titanteller TT13 titanium 13 mm flat tip or by cell disruption using a Constant Systems cell disruptor. A Fisherbrand Hydrus 300 pH meter was used to perform pH measurements (with a two point calibration at pH 4 and 7). PCR reactions were performed using a Techne TC-512 thermocycler. A Fisherbrand horizontal electrophoresis unit with a connected Power 300 electrophoresis power supply was used for running agarose gels. An Uvitec uvivue transilluminator with CCTV screen was used for visualisation of agarose gels. A Hoefer SE 260-min-vertical gel electrophoresis unit was used for running SDS-PAGE gels. Centrifugation, on a small scale, was performed using a Fisher Scientific accuSpin microcentrifuge or a Thermo Scientific IEC CL30R centrifuge. Large-scale centrifugation was performed using a Beckman Coulter Avanti JXN-26 centrifuge. High and low resolution mass spectra that were recorded at the University of St Andrews were aquired on a either a Waters Micromass LCT time of flight mass spectrometer coupled to a Waters 2975 HPLC system or on an Orbitrap ELOS pro HPLC purification was performed using a Gilson 800 HPLC system equipped with a 322 pump and a UV/Vis-151 detector using UniPoint software. UPLC analysis was performed using a Waters Acquity UPLC equipped with fluorescence and photodiiode array detectors**.** Ion exchange chromatography was performed using an Äkta Pure chromatography system equipped with a 5 ml HiTra SP-FF column from GE Healthcare Life Sciences. DNA sequencing was performed by either the DNA Sequencing Facility at the University of Cambridge using an Applied Biosystems 3730xl DNA analyser or by the DNA sequencing company GATC Biotech using an equivalent sequencing system. DNA was quantified by UV-vis using a BMG Labtech Omega Star plate reader.

General media, buffers and stock solutions used in this study.

Chemicals were purchased from Alfa Aesar, BD Biosciences, Fisher Scientific, ForMedium, Melford or Sigma Aldrich unless otherwise indicated. All measurements are taken as weight per volume (*w/v*) unless otherwise stated. All media were prepared by bringing to volume with sterile water (18 MΩ or autoclaved) followed by sterilisation in an autoclave for 20 min at 121 °C and 1.3 bar unless otherwise stated. For details of the media and buffers solutions utilized in this study see Supplementary Table 7.

Construction of *Escherichia coli* RG-1500 capable of producing 7-Br-tryptophan 2.

We created an *E. coli* strain capable of generating 7-Br-tryptophan **2** by transforming the recombinant plasmid of pSG36 containing a two-component halogenase system into *E. coli* K-12 PHL644 (MC4100 *malA-kan ompR234*),[22] a robust strain extensively used by Goss group for biosynthesis of L-halo-tryptophans in previous studies.[23-25] The two component halogenase system is composed of *prnA*, a halogenase gene from *Pseudomonas fluorescens* BL915 encoding for the tryptophan 7-halogenase *prnA* involved in pyrrolnitrin biosynthesis,[4,26,27] and an *ssuE* gene coding for the mesophilic flavin reductase *ssuE* from *E. coli* DH10B.[5] The construction of the recombinant plasmid of pSG36 is summarized in Supplementary Fig. 6. First, the *P. fluorescens* BL915 tryptophan 7-halogenase gene *prnA* was amplified from plasmid pCIB7805[4] (obtained from Prof. van Pée) using the primers of prnA-1 and prnA-2 as listed in Supplementary Table 9. The PCR product containing *prnA* was digested with *Nde*I and *Hin*dIII and then ligated to the large fragment of pET21a (Addgene, Cambridge, MA, USA) resulting from digestion with the same restriction enzymes, creating pSG15. Next, a DNA fragment containing a ribosome binding site (RBS) and *ssuE* was amplified by PCR from DH10B genomic DNA using the primers of ssuE-3 and ssuE-4 (listed in Supplementary Table 9). The PCR product was inserted into the *Hin*dIII site of pSG15 to give pSG32. A DNA fragment containing *prnA*, RBS sequence derived from *E. coli* DH10B and *ssuE* was amplified from pSG32 using primers of prnA-4 and ssuE-5. Restriction digest with *Nco*I and *Sal*I gave a DNA fragment containing *prnA*, RBS and *ssuE* that was subsequently ligated into the *Nco*I/*Sal*I-digested pSG29, resulting in the expression plasmid of pSG36. pSG29 was prepared by PCR amplification of pPro24(s)-gfp using primers of pPro-1 and pPro-2 to remove the *gfpuv* gene encoding the UV-excitable green fluorescent protein (GFP) and introduce three restriction sites (XhoI, NcoI and SalI) in the multiple cloning site. The PCR product was then digested with XhoI and the cut ends were ligated together to generate circular pSG29.

The recombinant plasmid of pSG36 containing the reengineered expression cassette composed of the propionate-inducible *prpBCDE* promoter (P*prpB*),[28] the *prnA/ssuE* tandem and the T1 transcriptional terminator,[29] was purified from the *E. coli* XL1-Blue culture and then transformed into the chemically competent cell of *E. coli* PHL644 for expression of the inserted halogenase system as described above. Positive clones were selected on LB agar plate containing 100 μg ml^{-1} ampicillin and 50 μg ml^{-1} kanamycin.

Analysis of the level of 7-Br-tryptophan 2 generated *in vivo* by *E. coli* RG-1500 culture after L-tryptophan 1 feeding.

The amount of 7-Br-tryptophan **2** produced by *E. coli* RG-1500 culture was determined as follows. Cells were lysed by freeze-thaw method and centrifuged (13,000 rpm, 16,060 × g, 10 min). Clear supernatant was collected, diluted with equal volume of methanol and analysed by LC-HRMS and UPLC. LC-HRMS analysis (summarized in Supplementary Fig. 7) revealed peaks of the expected mass (*m/z* 283.0073 and 285.0052), confirming the *in vivo* generation of 7-Br-tryptophan **2** by *E. coli* RG-1500. For UPLC analysis, the PDA was observed at 280 nm. The calibration curve for 7-Br-tryptophan **2** was linear in 15.6 - 500 μM concentration range (as shown in Supplementary Fig. 9) and was used for quantification. After

24 h of reaction, 212 µM of 7-Br-tryptophan **2** could be seen to have been produced within the induced culture. In parallel, for the *E. coli* RG-1500 culture grown without the inducer, UPLC analysis revealed absence of any bromination product (overlaid UPLC chromatograms for samples from bromination reaction are shown in Supplementary Fig. 8).

For LC-HRMS analysis, the HPLC separation was conducted on a Thermo Scientific Dionex Ultimate 3000 Rapid Separation LC system using XBridge BEH C18 column (130Å, 3.5 µm, 2.1 × 100 mm). The flow rate was set to 0.20 ml min^{-1} and the column temperature was maintained at 40 °C. A generic binary gradient elution was carried out using different ratios of eluents A (water containing 0.1% formic acid) and B (acetonitrile). Following gradient was used: 0-0.5 min (5% B), 0.5-1.5 min (5% to 15%B), 1.5-7.5 min (15%B), 7.5-15.5 min (15% to 95% B), 15.5-17.5 min (95%B), 17.5-18.0 (95%-5%B) and 18.0-20.0 (5% B). The total LC run time is 20 min. The mass spectrometric analysis was performed on an Orbitrap Velos Pro mass spectrometer system equipped with a Thermo Scientific Ion MAX API source housing. The MS conditions were as follows: heated electrospray ionization (HESI-II) probe, positive ionization mode, spray voltage 3.5 kV, capillary temperature 350 °C, normalized collision energy 35% for collision induced dissociation (CID), and sheath gas and auxiliary gas flow rates of 35 and 10 arbitrary units, respectively. Survey full scan MS spectra (from *m/z* 100-1000) were acquired in the Orbitrap with resolution R=60K.

Culturing *E coli* RG-1500 and cross-coupling the 7-Br-tryptophan 2 produced by the culture *in vivo*: Full details.

E. coli RG-1500 culture containing *in vivo* generated 7-Br-tryptophan **2** (5 ml) was mixed with phosphate buffer (45 ml, 10 mM, pH 8.5) in a sterile 250 ml flask. Filter sterilized solutions of *p*-Tol-$B(OH)_2$ (final concentration 1 mM, 0.5 ml, 100 mM stock in 90% aqueous ethanol) and appropriate Pd-catalyst (final concentration 50 µM, 0.25 ml, **L3-Pd** or **L4-Pd** 10 mM stock in water). Culture reaction flasks were incubated for 4 h (37 °C, 200 rpm, incubator throw 19 mm). Control reactions with only Pd-catalyst, only *p*-Tol-$B(OH)_2$ and without any reagents were also conducted. Cross-coupling was quenched by addition of DTT (final concentration 1 mM, 0.05 ml, 1 M stock in water). An aliquot (1 ml) was collected, cells were lysed by five freeze-thaw-vortex cycles (5 min of cells freezing with liquid nitrogen, 2 min of thaw at 42 °C in a water bath, followed by 2 min of a vigorous vortex) and centrifugation (13,000 rpm, 16,060 × g, 5 min) to remove insoluble particulates. Clear supernatant was collected diluted with equal volume of methanol and analysed by LC-HRMS and UPLC. LC-HRMS analysis revealed peaks representing 7-(*p*-tolyl)-tryptophan **2a** (*m/z* 295.1436) and deaminated 7-(*p*-tolyl)-tryptophan (*m/z* 278.1171) (as shown in Supplementary Fig. 10). For UPLC analysis, fluorescence emission was detected using excitation wavelength of 295 nm and emission wavelength of 370 nm (UPLC method 1). The calibration curve for 7-(*p*-tolyl)-tryptophan was linear in 0.78-12.5 µM concentration range, as shown in Supplementary Fig. 2, and was used for quantification. The concentration of 7-(*p*-tolyl)-tryptophan **2a** in the reaction samples measured by UPLC was 12.92 µM with **L4-Pd** treatment and 5.38 µM with **L3-Pd** treatment (Supplementary Table 5). In parallel, for the control reactions, UPLC analysis revealed absence of any cross-coupling product. Overlaid UPLC chromatograms for samples from cross-coupling reaction are shown in Supplementary Fig. 11.

Construction of *Streptomyces coelicolor* RG-1104 capable of producing Br-pacidamycin 3.

The pacidamycins are naturally produced by *Streptomyces coeruleorubidus*.[6] We had previously engineered this wild type strain introducing the gene encoding tryptophan 7-halogenase, *prnA*. The resultant strain RG-5059 was capable of generating Cl-pacidamycin.[3] Culturing was performed as for

metabolite production, however the cultures supplemented with 50, 100, 150, 200 mM NaBr, or NH_4Br grew poorly; and pacidamycin production was low under these conditions while bromopacidamycin production was not observed. *Streptomyces coelicolor* M1154 cultures grew well under the same conditions. In order to access brominated analogues of pacidamycin, we set to create a synthetic biological strain in which the genes encoding pacidamycin biosynthesis were introduced to *Streptomyces coelicolor* M1154 as it would be tolerant to supplementation with bromide salts.

Integration of the pacidamycin biosynthetic genes (*pac1- pac21*) into the heterologous expression host *Streptomyces coelicolor* M1154. Generation of strain RG-4242.

The genetically-characterised *S. coelicolor* M1154 host strain has been optimised by ribosomal engineering and removal of native natural product pathways.[30] Its growth was not impaired by the addition of bromide salts, and it was therefore an excellent candidate for heterologous expression of the pacidamycin pathway. Introduction of the minimal pacidamycin biosynthetic genes, previously located on *S. coeruleorubidus* cosmid 2H-5[31] into the genome of the *S. coelicolor* M1154 heterologous host strain was pursued. Since Supercos 1-derived cosmids are unable to replicate in *Streptomyces*, cosmid 2H-5 was first modified for conjugal transfer and site-specific integration into the target genome.

Introduction of a PCR-targeted integration cassette onto *S. coeruleorubidus* cosmid 2H-5.

Generation of the PCR-targeted integration cassette: A PCR-targeting system for *Streptomyces* was available[31] and employs the λ red recombinase functions to facilitate homologous recombination between PCR-amplified linear DNA with terminal homology to a targeted region on a plasmid. This system was used to replace the *neo* (kanamycin resistance) gene on the cosmid 2H-5 backbone with the integration cassette located on cosmid pIJ10702, this will allow transfer and integration into the target heterologous host. The *aac*(3)*IV*-*ori*T-*att*PΦC31-*Int*ΦC31 integration cassette from plasmid pIJ10702 was purified from an agarose gel as a ~ 5.2 Kb *Ssp* I-fragment. The integration cassette was then PCR-amplified from the purified template using the specific in-frame primers ScospIJ10702DvF2/R2 which contain 5'-DNA sequence identical to the regions flanking the Supercos I *neo* gene. PCRs were performed as follows: 50 ng template, 1X *Pfu* Buffer, 0.2 μM dNTPs (A, C, G, T, Sigma), 5% DMSO, 2.5 U *PfuTurbo* polymerase (Stratagene), 1 μM forward and reverse primer in a 50 μl reaction. Cycling: Initial denaturation; 3 minutes at 94 °C, (10X) Denaturation; 1 minute at 94 °C, Annealing; 1 minute at 60 °C, Extension; 16 minutes at 72 °C, (15X) Denaturation; 1 minute at 94 °C; Annealing; 1 minute at 65 °C, Extension; 16 minutes at 72 °C, (1X) Extension; 15 minutes at 72 °C, hold at 4 °C. The amplified DNA was then assessed by gel electrophoresis and purified from an agarose gel.

PCR-targeted replacement of the *neo* gene with the integration cassette: *E. coli* BW25113/pIJ790 containing cosmid 2H-5 was grown to mid-log phase at 30 °C (to maintain the temperature sensitive pIJ790 plasmid) in selective media and the λ red recombinase genes on pIJ790 induced with 10 mM L-arabinose before preparation of electrocompetent cells. The PCR-targeted integration cassette (100 ng) (*aac*(3)*IV*-*oriT*-*att*PΦC31-*Int*ΦC31) was then introduced by electroporation into *E. coli* BW25113/pIJ790/cosmid 2H-5 for λ-red mediated recombination between the homologous regions on the PCR-amplified resistance cassette and cosmid 2H-5. Transformants were plated on LB agar containing apramycin and ampicillin to select for the mutated cosmid with incubation at 37 °C to promote loss of the heat sensitive plasmid pIJ790. The acquired apramycin-resistant *E. coli* phenotype indicates that correct recombination has occurred with the cassette. The new cosmid was designated Cosmid 2H-5-Int. The integrity of the *S. coeruleorubidus* DNA on the cosmid was assessed by BamHI and XhoI restriction digestion. BamHI cuts once near the terminus of the cosmid backbone and at known sites in the *S. coeruleorubidus* DNA insert. The introduction of the integration cassette (lacking BamHI restriction sites) onto the cosmid should not alter the BamHI restriction digestion pattern as verified by restriction

digestion. However, the integration cassette contains an XhoI restriction site and an altered XhoI restriction pattern was expected, and confirmed, for cosmid 2H-5-Int (Supplementary Fig. 14).

Conjugal transfer of cosmid 2H-5-Int DNA to *S. coelicolor* M1154 to generate strain RG-4242.

Cosmid 2H-5-Int was introduced into the *S. coelicolor* M1154 host strain by conjugation from methylation-deficient *E. coli* ET12567/pUZ8002 (to reduce restriction of incoming methylated DNA). The *E. coli* ET12567/pUZ8002/cosmid 2H-5-Int cultures were grown to the mid log phase in selective medium, washed twice in 1 volume LB medium and resuspended cells mixed with heat-shocked *S. coelicolor* spores on SFM-agar supplemented with 10 mM $MgCl_2$. After 20 h at 28-30 °C, conjugation plates were overlaid with 3 ml soft TSB agar containing 0.5 mg nalidixic acid and apramycin before further incubation at 28 °C for selection of *S. coelicolor* M1154 *ex*-conjugants. The conjugations were monitored against a negative *E. coli* ET12567/pUZ8002 control and a positive *E. coli* ET12567/pUZ8002/cosmid 2H-5-Int growth control overlaid only with nalidixic acid. Putative *S. coelicolor* M1154 exconjugants were patch-plated onto selective ISP2-TSB media containing nalidixic acid and apramycin for initial growth and then patched onto selective ISP2-TSB agar containing either apramycin or kanamycin. Strains with the correct $apr^{R}kan^{S}$ phenotype were then purified and spore suspensions prepared. These are expected to have the biosynthetic genes located in the heterologous host genome and the strain taken forwards was designated as RG-4242.

Pacidamycin production by *S. coelicolor* RG-4242.

The production of pacidamycins in the heterologous expression strain *S. coelicolor* RG-4242 was established by analysis of the crude extract from small-scale ISP2 cultures by LC-MS. As expected, pacidamycins were not detected in extracts from parent *S. coelicolor* M1154 strains, included as a negative control, but those containing the pacidamycin genes acquired the ability to produce a new compound of the same mass and retention time as pacidamycin D (*m/z* 712). To establish the identity of the new compound produced by the *S. coelicolor* strain RG-4242 as pacidamycin D, LC-MS/MS analysis was performed. Fragmentation analysis of this new compound confirmed that this was identical in retention time and fragmentation pattern to pacidamycin D produced by the natural *S. coeruleorubidus* host (Supplementary Fig. 17). This confirms that the core pacidamycin biosynthetic genes have been heterologously expressed in the alternative host strain. The wild type producer, *S. coeruleorubidus*, naturally generates a complex suite containing at least 8 naturally occurring analogues of pacidamycin, differing in the amino acids encorporated. Usefully significant simplification is observed within the synthetic biological strain RG-4242. The integrated genes produce a subset of the pseudo-tetrapeptide members of the pacidamycin suite since the genes encoding enzymes necessary for installation of an additional amino acid are located elsewhere.[32] This offers the advantage of an increasingly simplified pacidamycin profile for analysis.

Assessment of *S. coelicolor* RG-4242's tolerance to bromide salts, and impact on pacidamycin production.

The impact of bromide and chloride salts on growth of *S. coelicolor* RG-4242 was assessed. Duplicate cultures supplemented with either 0, 50, 100, 150 or 200 mM sodium chloride or bromide were grown. Unlike the natural producer, *S. coeruleorubidis* the heterologous expression strain *S. coelicolor* RG-4242 was capable of producing good levels of both pacidamycin D and S in the presence of both bromide and chloride salts. The samples were analysed by LC-MS and the EICs were integrated to obtain the peak area. RG-4242 produced pacidamycin D and S with concentrations of up to 200 mM sodium chloride and sodium bromide (Supplementary Fig. 19) within the culture medium. This screen revealed that good levels of pacidamycin production were observed with concentrations of salts ranging between 50-100

mM. Production of pacidamycin D was higher than pacidamycin S in RG-4242. Notably, increasing salt concentration, up to 200 mM, did not have a negative effect on pacidamycin production as was observed for the WT producer *S. coeruleorubidus*. Results also indicated that production of both pacidamycin D and S in RG-4242 was slightly higher in the presence of the NaBr rather than NaCl. As with many actinomycetes natural products the production of the pacidamycins is very variable and consistency is challenging to achieve, and considerable differences can be seen across the duplicates. The factors governing the variability of secondary metabolite production within *Streptomyces* are still not well understood.[2] These experiments clearly demonstrate that in contrast to the natural pacidamycin producing strain, the synthetic biological strain tolerates NaBr well and the pacidamycin production is not impeded.

Chromosomal integration of *prnA* into the heterologous production host *S. coelicolor* RG-4242, generation of RG-1104.

Once it was established that the engineered *S. coelicolor* M1154 RG-4242 could tolerate both chloride and bromide salt enriched media, the next step was to introduce *prnA*, the tryptophan 7-halogenase gene, into *S. coelicolor* RG-4242 on plasmid pSG19. Plasmid pSG19, containing the halogenase gene, was introduced into *E. coli* ET12567 carrying pUZ8002 before transfer to RG-4242 by conjugation, as previously described for *S. coeruleorubidus*.[3] The resulting strain was then designated as RG-1104.

Production of Br-pacidamycin D 3 from RG-1104.

Cultures of RG-1104 were grown using the starter culture media (2×YT) and the main culture media (ISP2) were supplemented with 50 mM NaBr instead of NaCl. After the 8 day incubation period, extraction with XAD16 followed by elution with methanol was performed. After concentration of the samples under reduced pressure, fractionation of the sample using a diaion column was carried out (eluting with 0, 25, 50, 75, 100% methanol:water) collecting 250 ml fractions. The fractions were analyzed *via* LC-MS and Br-pacidamycin D **3** was detected in fractions eluted with 75% MeOH (Supplementary Fig. 20, 21).

Culturing *S. coelicolor* RG-1104 in cross-coupling media and *in vivo* cross-coupling the Br-pacidamycin D 3 produced by the culture.

We first established whether CCM for pacidamycin production, in place of ISP2 which we had been using as the main media. After incubation of the main culture in CCM for 5 days LC-MS analysis confirmed the presence of Br-pacidamycin D **3** (Supplementary Fig. 22).

Cross-coupling was performed with cultures of *S. coelicolor* RG-1104 grown in CCM on day 5-6 when LCMS analysis of an aliquot of the culture had confirmed good levels of Br-pacidamycin-D **3**. An appropriate *S. coelicolor* culture containing Br-pacidamycin D **3** (5 ml) was diluted with phosphate buffer (45 ml, 10 mM, pH 8.5) in a sterile 250 ml flask. Filter sterilized solutions of *p*-Tol-B$(OH)_2$ (final concentration 1 mM, 0.5 ml of 100 mM stock in 90% aqueous ethanol) and appropriate Pd-catalyst (**L3-Pd** or **L4-Pd**, final concentration 25 µM, 125 µl of 10 mM stock in water) were added. The reaction culture flasks were incubated at 37 °C (200 rpm, incubator throw 19 mm) for 4 h. The cross-coupling was quenched by addition of DTT (25 µl of 1 M stock). An aliquot (1 ml) was collected, cells were lysed by 5 freeze-thaw cycles and centrifuged (13000 rpm, 16060 × g, 5 min). Clear supernatant was collected and analysed by LC-MS. Analysis of the supernatant revealed complete consumption of Br-pacidamycin-D **3** (Supplementary Fig. 25) and the presence of a peak of the expected mass 802.3523 *m/z*. The identity of the cross-coupled product was confirmed *via* MS/MS2 analysis (Supplementary Fig. 23-24).

SUPPLEMENTARY REFERENCES

1. Dong, X, Stothard, P., Forsythe, I. J. & Wishart, D. S. PlasMapper: A web server for drawing and auto annotating plasmid maps. *Nucleic Acids Res*. **32**, 660-664 (2004).
2. Gust, G., Challis, G., Fowler, K., Kieser, T. & Chater, K. F. PCR-targeted *Streptomyces* gene replacement identifies a protein domain needed for the biosynthesis of sesquiterpene soil odor geosmin. *Proc. Natl. Acad. Sci. USA.* **100**(4), 1541-1546 (2003).
3. Roy, A. D., Grüschow, S., Cairns, N. & Goss, R. J. M. Gene expression enabling synthetic diversification of natural products: Chemogenetic generation of pacidamycin analogs. *J. Am. Chem. Soc.* **132**, 12243-12245 (2010).
4. Hammer, P. E., Hill, D. S., Lam, S. T., van Pée, K. H. & Ligon, J. M. Four genes from *Pseudomonas fluorescens* that encode the biosynthesis of pyrrolnitrin. *Appl. Environ. Microbiol.* **63**, 2147-2154 (1997).
5. Van der Ploeg, J. R., Iwanicka-Nowicka, R., Bykowski, T., Hryniewicz, M. M. & Leisinger, T. The *Escherichia coli* ssuEADCB gene cluster is required for the utilization of sulfur from aliphatic sulfonates and is regulated by the transcriptional activator CbI. *J. Biol. Chem.* **274**, 29358-29365 (1999).
6. Winn, M., Goss, R. J. M., Kimura, K & Bugg, T. D. Antimicrobial nucleoside antibiotics targeting cell wall assembly: Recent advances in structure-function studies and nucleoside biosynthesis. *Nat. Prod. Rep.* **27**, 279-304 (2010).
7. Adamczyk-Woźniak, A., Komarovska-Porokhnyavets, O., Misterkiewicz, B., Novikov, V. P. & Sporzyński, A. Biological activity of selected boronic acids and their derivatives. *Appl. Organometal. Chem.* **26**, 390–393 (2012).
8. Gao, Z., Gouverneur, V. & Davis, B. G. Enhanced aqueous Suzuki-Miyuara coupling allows site-specific polypeptide ^{18}F-labeling. *J. Am. Chem. Soc*. **135**, 13612−13615 (2013).

9. Li, N., Lim, R. K. V., Edwardraja, S. & Lin, Q. Copper-free Sonogashira cross-coupling for functionalization of alkyne-encoded proteins in aqueous medium and in bacterial cells. *J. Am. Chem. Soc*. **133**, 15316–15319 (2011).

10. Smith, D. R. M. *et al.* The first one-pot synthesis of L-7-iodotryptophan from 7-iodoindole and serine, and an improved synthesis of other L-7-halotryptophans. *Org. Lett*. **16**, 2622-2625 (2014).

11. Corr, M. J. *et al*. Sonogashira diversification of unprotected halotryptophans, halotryptophan containing tripeptides; and generation of a new to nature bromo-natural product and its diversification in water. *Chem. Sci.* **8**, 2039-2046 (2017).

12. Spicer, C. D. & Davis, B. G. Rewriting the bacterial glycocalyx *via* Suzuki-Miyaura cross-coupling. *Chem. Commun.* **49**, 2747-2749 (2013).

13. Qiu, P., Zhao, J. Y., Shi, X. & Duan, X. H. An efficient water-soluble surfactant-type palladium catalyst for Suzuki cross-coupling reactions in pure water at room temperature. *New J. Chem*. **40**, 6568-6572 (2016).

14. Kanchupalli, V., Joseph, D. & Katukojvala S. Pyridazines N-oxides as precursors of metallocarbenes: rhodium catalyzed tranannulation with pyrroles. *Org. Lett*. **17**, 5878−5881 (2015).

15. Roy, A. D., Goss, R. J. M., Wagner, G. K. & Winn, M. Development of fluorescent aryltryptophans by Pd mediated cross-coupling of unprotected halotryptophans in water. *Chem. Commun.*, 4831–4833 (2008).

16. Hardee, D. J., Kovalchuke, L. & Lambert, T. H. Nucleophillic acyl substitution *via* aromatic cation activation of carboxylic acids: rapid generation of acid chlorides under mild conditions. *J. Am. Chem. Soc.* **132**, 5002-5003 (2010).

17. Marfey, P. Determination of D-amino acids II. Use of a bifunctional reagent, 1,5-difluoro-2,4-dinitrobenzene. *Carlsberg Res. Commun.* **49**, 591-596 (1984).

18. Kieser, T., Bibb, M. J., Buttner, M. J., Chater, K. F. & Hopwood, D. A. *Practical Streptomyces Genetics 2nd Ed* (John Innes Foundation, Norwich, 2000).

19. Sambrook, J. & Russell, D. W. *Molecular Cloning: A Laboratory Manual 3rd Ed* (Spring Habour Laboratory Pressn New York, 2001).

20. Sano, T. & Cantor, C. R. Expression of a cloned streptavidin gene in *Escherichia coli. Proc. Natl. Acad. Sci.* USA. **87**,142-146 (1990).

21. Grüschow, S., Rackham, E. J., Elkins, B., Newill, P. L. A. & Goss, R. J. M. New pacidamycin antibiotics through precursor-directed biosynthesis. *ChemBioChem.* **10**, 355-360 (2008).

22. Vidal, O., Longin, R., Prigent-Combaret, C., Dorel, C., Hooreman, M. & Lejeune, P. Isolation of *Escherichia coli* K-12 mutant strain able to form biofilms on inert surfaces: involvement of a new ompR allele that increases curli expression. *J. Bacteriol*. **180**, 2442-2449 (1998).

23. Tsoligkas, A. N. *et al.* Engineering biofilms for biocatalysis. *ChemBioChem*. **12**, 1391-1395 (2011).

24. Perni, S., Hackett, L., Goss, R. J. M., Simmons, M. J. & Overton, T. W. Optimisation of engineered *Escherichia coli* biofilms for enzymatic biosynthesis of L-halotryptophans. *AMB Express*. **3**, 66 (2013).

25. Tong, X. *et al.* Rapid enzyme regeneration results in the striking catalytic longevity of an engineered, single species, biocatalytic biofilms. *Microb. Cell Fact*. **15**, 180 (2016).

26. Keller, S., Wage, T., Hohaus, K., Hölzer, M., Eichhorn, E. & van Pée, K. H. Purification and partial characterization of tryptophan-7-halogenase (*PrnA*) from *Psudeomonas fluorescens*. *Angew. Chem. Int. Ed. Engl.* **39**, 2300-2302 (2000).

27. Dong, C., Kotzsch, A., Dorward, M., van Pée, K. H. & Naismith, J. H., Crystallization and X-ray diffraction of a halogenating enzyme, trptophan 7-halogenase, from *Pseudomonas fluorescens. Acta Crystallogr. D Biol. Crystallogr.* **60**, 1438-1440 (2004).

28. Lee, S. K. & Keasling, J. D. Propionate-regulated high-yield protein purification in *Escherichia coli*. *Biotechnol. Bioeng*. **93**, 912-918 (2006).

29. Orosz, A., Boros, I. & Venetianer, P. Analysis of the complex transcription termination region of the *Escherichia coli* rrnB gene. *Eur. J. Biochem*. 201, 653-659 (1991).

30. Gomez-Escribano, J. P. & Bibb, M. J. Engineering *Streptmyces ceolicolor* for heterologous expression of secondary metabolite gene clusters. *Microb Biotechnol.* **4**, 207-215 (2011).

31. Rackham, E. J., Grüschow, S., Ragab, A. E., Dickens, S. & Goss, R. J. M. Pacidamycin biosynthesis: Identification and heterologous expression of the first uridyl peptide antibiotic gene cluster. *ChemBioChem.* **11**, 1700-1709 (2010).

32. Grüschow, S., Rackham, E. J. & Goss, R. J. M. Diversity in natural product families is governed by more than enzyme promiscuity alone: establishing control of pacidamyin portfolio. *Chem. Sci*. **2**, 2182-2186 (2011).
